# Design, Synthesis, and Structure-Activity Relationships of Novel Piperidine-Fused Imidazolone ClpP Activators as Potential Anti-Cancer Agents

**DOI:** 10.3390/molecules31162916

**Published:** 2026-08-20

**Authors:** Shanshan Chen, Xinyi Lin, Min Guo, Ruqi Lei, Beijing Chen, Yubo Zhou, Aijun Qiao, Qi Huang, Mingliang Wang

**Affiliations:** 1 School of Chinese Materia Medica, Nanjing University of Chinese Medicine, Nanjing 210023, China; chenshanshan1064@zidd.ac.cn (S.C.); guomin1613@zidd.ac.cn (M.G.); 2 School of Pharmaceutical Sciences, Southern Medical University, Guangzhou 510515, China; linxinyi310@zidd.ac.cn; 3 Guangzhou University of Chinese Medicine, Guangzhou 510006, China; leiruqi1309@zidd.ac.cn; 4 Zhongshan Institute for Drug Discovery, Zhongshan 528400, China; chenbeijng@zidd.ac.cn; 5 Shanghai Institute of Materia Medica, Chinese Academy of Sciences, Shanghai 201203, China

**Keywords:** ClpP, structure-guided drug design, small molecule activator, anti-tumor

## Abstract

Caseinolytic protease P (ClpP) is essential for maintaining mitochondrial protein homeostasis, and its activation has emerged as an attractive cancer therapeutic strategy. However, **ONC201** is currently the only approved ClpP activator, and its low potency leads to high clinical doses, driving an urgent demand for highly potent agents. In this study, utilizing a ring-opening-based molecular simplification strategy, we identified a class of activators based on a novel piperidine-fused imidazolone scaffold. Through side-chain optimization, we ultimately obtained **CLPP-3036** as a highly potent compound. **CLPP-3036** exhibits nanomolar enzymatic potency (EC_50_ = 6.02 nM, 146-fold more potent than **ONC201**) and excellent antiproliferative activity (MV4-11 IC_50_ = 1.13 nM). Importantly, **CLPP-3036** demonstrates significantly superior inhibitory activity compared to **ONC201** across multiple hematologic and solid tumor cell lines, such as Raji cells (IC_50_ = 9.8 nM, nearly 1260-fold more potent). Furthermore, **CLPP-3036** promotes the degradation of ClpP substrate proteins and effectively triggers apoptosis in MV4-11 cells. Collectively, **CLPP-3036** is a potent piperidine-fused imidazolone ClpP activator and represents a promising lead compound worthy of further study.

## 1. Introduction

Caseinolytic protease P (ClpP) is a serine protease localized within the mitochondrial matrix [1]. It plays a pivotal role in maintaining mitochondrial proteostasis [2,3] and respiratory chain integrity by degrading damaged or misfolded proteins [4,5]. Dysregulated ClpP expression leads to mitochondrial dysfunction, which is closely associated with multiple pathological processes, including oncogenesis [6,7]. Recent studies have revealed that ClpP is overexpressed in lymphomas, acute myeloid leukemia (AML), and several solid tumors [7,8,9,10,11]. Therefore, ClpP has emerged as a novel anticancer target in recent years and has attracted widespread attention [12,13].

**Figure 1:**
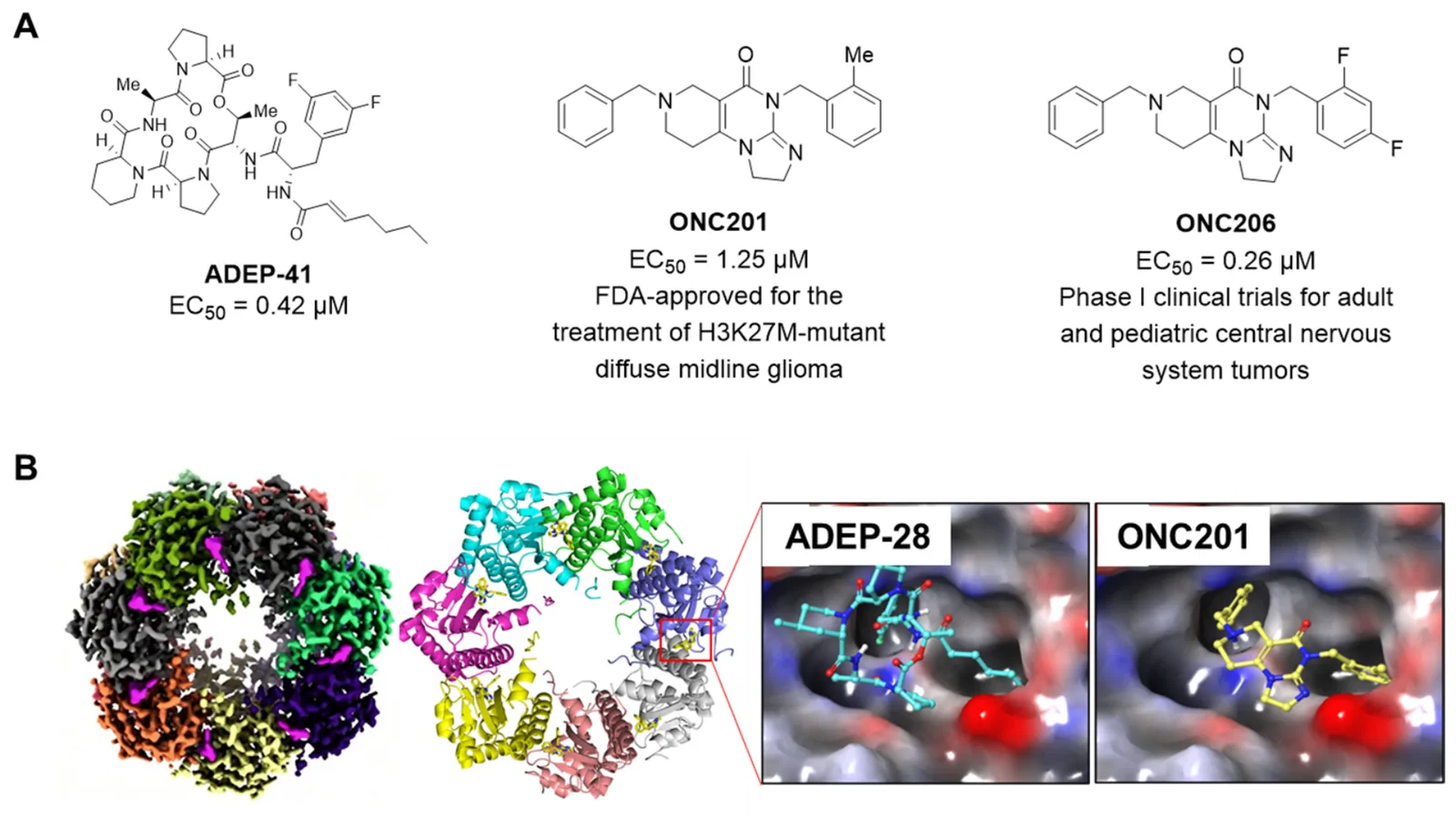
(**A**) Representative small-molecule activators of ClpP. (**B**) Cocrystal structures of the activators **ONC201** and **ADEP-28** with ClpP.

ClpP is composed of two adjacent cylindrical heptamers, with the active sites typically located between two subunits. ClpP requires association with its chaperone ClpX and ATP hydrolysis to execute its protein degradation function [14,15]. While ClpP expression in tumor cells is 2~10-fold higher than that in normal cells, its proteolytic activity is paradoxically suppressed owing to the limited availability of ClpX and ATP. Small molecule activators occupy the active sites between subunits of ClpP, thereby inducing uncontrolled degradation of mitochondrial proteins, triggering mitochondrial collapse, energy depletion and oxidative stress, and ultimately inducing cell cycle arrest and apoptosis in tumor cells (Figure 1) [16,17]. Consequently, the activation of ClpP by small-molecule activators has emerged as a promising tumor therapeutic strategy [18].

Research on ClpP activators began with the discovery of acyldepsipeptides and their derivatives (**ADEP-41**, **ADEP-28**), which were the first compounds demonstrated to exert antitumor activity through targeted regulation of ClpP function (Figure 1A) [19]. With the deepening of research on the functional roles of ClpP, a various of small molecules modulating mitochondrial ClpP function have also been discovered. In 2019, **ONC201**, an imipridone-based antitumor agent, was identified as a ClpP activator, representing a major breakthrough in this field (Figure 1A) [20,21,22]. Building upon its unique core scaffold, several structurally optimized derivatives—including **ONC206** [23], **TR-57** [24], 1**6z** [25], **CLPP-1071** [26], **CLPP-2068** [27], **MS6076** [28], and **LZL25** [29]—have been successively reported, many of which have demonstrated potent antitumor efficacy in multiple cancer models. In terms of chemical structural scaffolds, both **ADEP-28** (cyclic peptide structure) and **ONC201** (tricyclic structure) share similar binding mechanisms, binding to the hydrophobic pocket of ClpP (Figure 1B). Currently, **ONC201** is the only FDA-approved ClpP activator and is indicated for adult and paediatric patients aged 1 year and older with relapsed or progressive diffuse midline glioma (DMG) harbouring the H3K27M mutation, as confirmed by tumor sample testing [30]. However, **ONC201** exhibits relatively weak agonistic potency with an EC_50_ value of 1.3 μM. Furthermore, in Phase III clinical trials (NTC05580562), **ONC201** was tolerated at various dose levels (125–750 mg), resulting in a 12-month survival rate of 57% and a 24-month survival rate of 35%. At a dosage of 625 mg taken twice weekly in adults, 51.4% of patients experienced adverse events such as fatigue, nausea, and vomiting. **ONC206**, another imipridone-based ClpP activator, has advanced into Phase II clinical trials for advanced pheochromocytoma/paraganglioma (NCT07282587), recurrent grade 2 or 3 Meningioma (NCT07533942), whereas other reported ClpP activators remain in the preclinical stage [31]. Consequently, the discovery of novel small-molecule ClpP activators with higher efficiency remains a critical imperative.

**Figure 2:**
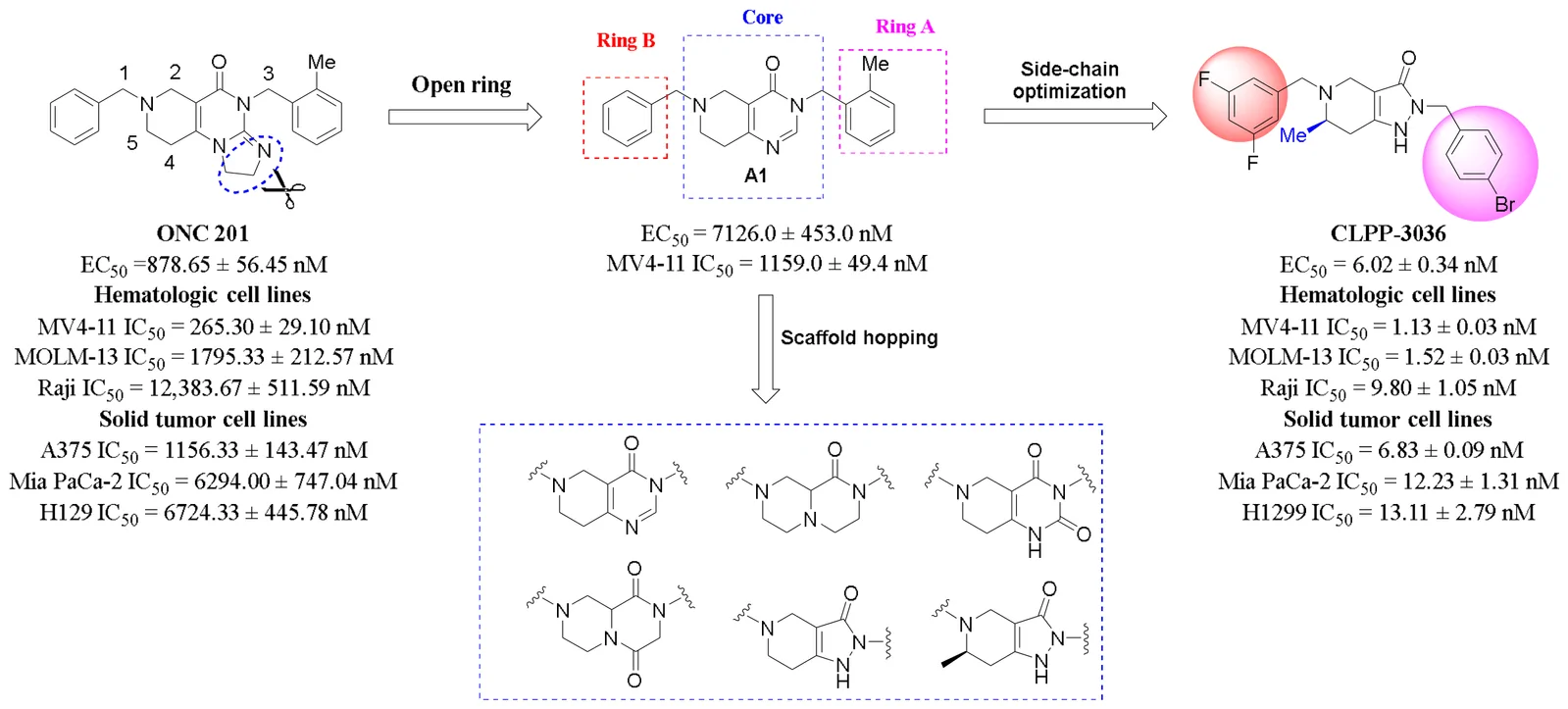
This work: Structure-guided design and optimization of ClpP activators.

In this work, we developed novel ClpP activators through a two-stage structure optimization based on structure-guided drug design. Stage 1: Optimizing the core through ring-opening-based molecular simplification strategy; Stage 2: modification of groups on the side arms to optimize ClpP activators. In the core optimization process, we first performed ring-opening of the imipridone scaffold of **ONC201**, obtaining compound **A1** with micromolar potency. Further scaffold-hopping optimization of the core of **A1** led to the discovery of compound **A8**, which features a piperidine-fused imidazolone scaffold. Subsequently, we modified the two side-chains of **A8** to improve potency, with a particular focus on exploring the structure-activity relationship (SAR) of different substitutions. Finally, a novel piperidine-fused imidazolone ClpP activator **CLPP-3036** was obtained with excellent potency. The selected compound **CLPP-3036** possesses nanomolar level ClpP activation potency (EC_50_ = 6.02 nM), it is 146-fold more potent than the approved molecule **ONC201**. Furthermore, **CLPP-3036** exhibited excellent anti-proliferative activity in multiple hematologic and solid tumor cell lines. For example, it exhibited an IC_50_ value of 1.13 nM against MV4-11 cells, far superior (240-fold) to **ONC201**, establishing it as a highly potent small-molecule ClpP activator. **CLPP-3036** also promotes the degradation of ClpP downstream proteins and triggers tumor cell apoptosis. Collectively, **CLPP-3036** could serve as a lead compound for the further development of ClpP-targeted therapeutics (Figure 2).

## 2. Results and Discussion

### 2.1. SAR Study

**Figure 3:**
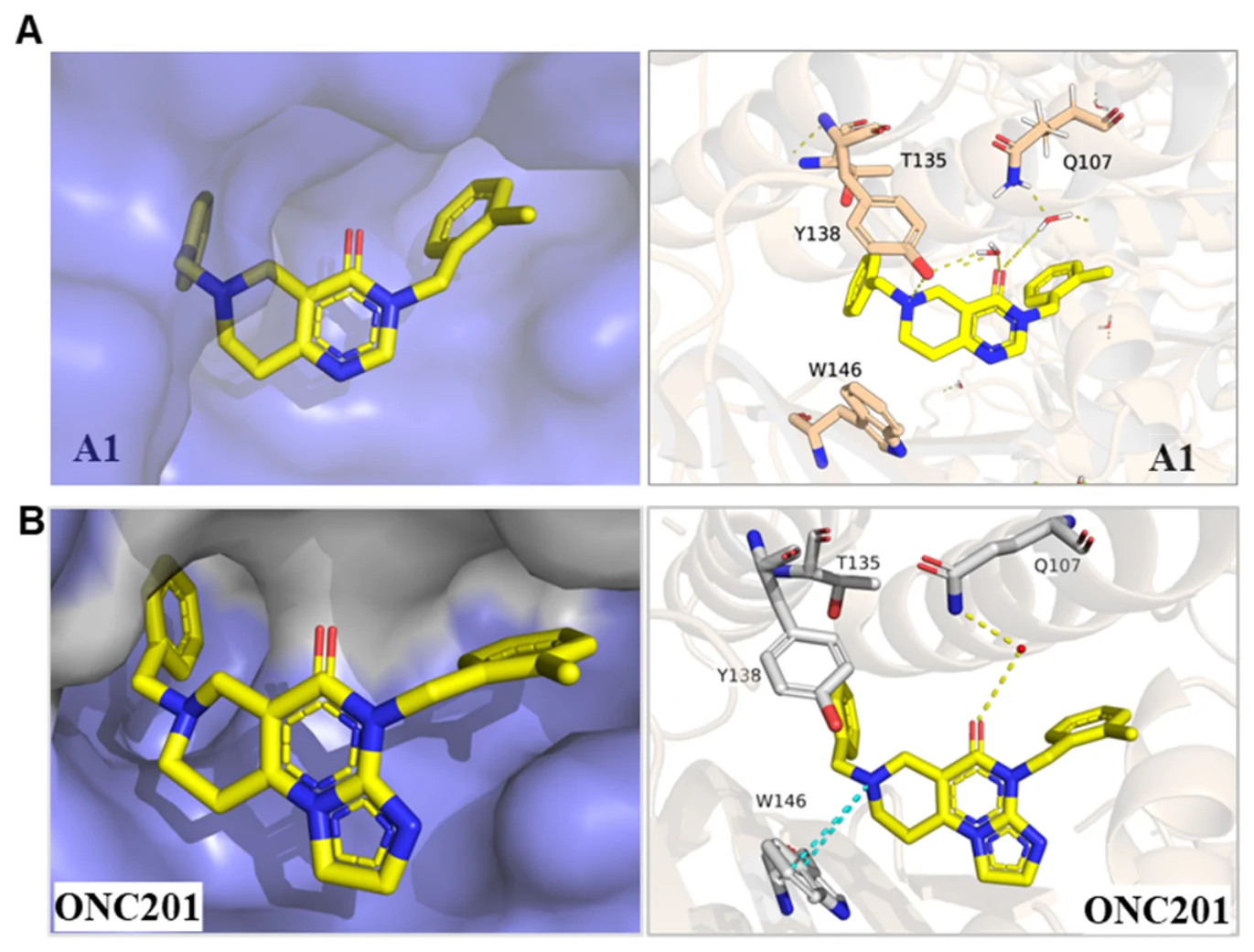
Structural comparison of **A1** and **ONC201** in the ClpP activator-binding pocket. (**A**) Docking-predicted binding pose of **A1** (Docking score = 9.14). (**B**) Co-crystal binding mode of **ONC201**. Key interacting residues are shown as sticks, and hydrogen bonds are indicated by dashed lines.

Using **ONC201** as the lead compound and retaining its side arms, we first employed a ring-opening strategy to remove the five-membered ring in the blue-highlighted region, thereby converting the original tricyclic core into a bicyclic scaffold and affording compound **A1** (Figure 1). **A1** was assessed for its ability to activate ClpP and suppress the proliferation of MV4-11 cells. Compared with **ONC201**, **A1** showed markedly reduced potency, with a ClpP activation EC_50_ of 7126.0 nM and a cellular proliferation IC_50_ of 1159.0 nM. To rationalize this activity loss, we examined the predicted binding mode of **A1** in the ClpP activator-binding pocket (Figure 3). Molecular docking suggested that **A1** adopt a binding mode distinct from that of **ONC201**. Removal of the nitrogen-containing five-membered ring reduced the asymmetry and conformational constraint of the tricyclic core, which may increase pose degeneracy. As a result, **A1** failed to maintain the W146-associated hydrogen-bonding network and the aromatic contacts involving the Y118/Y138 region that stabilize the **ONC201**-like binding pose. These observations indicated that preserving the orientational bias, three-dimensional shape, and key interaction geometry of the core scaffold is important for maintaining ClpP activation.

Although the nitrogen-containing five-membered ring is partially solvent-exposed, the substantial activity loss of **A1** suggests that this motif contributes to the conformational preorganization and directional binding of the **ONC201** core. Therefore, subsequent modifications at this region should preserve the asymmetric fused-core architecture rather than simply disrupt the ring system. In parallel, substituent optimization on the two aryl side arms may further improve shape complementarity, hydrophobic packing, and, where geometrically feasible, additional polar interactions with residues surrounding the pocket. Consistent with our previous findings, introducing an appropriately oriented methyl group at the defined position of the piperidine ring may occupy a hydrophobic subpocket associated with W146 conformational flipping, thereby strengthening ligand binding [27].

Based on the above analysis, we further carried out structural modification based on compound **A1**. As shown in Table 1, adjusting the nitrogen atom position on the ring of **A1** produced compound **A2** (EC_50_ = 5559.5 nM, IC_50_ = 349.2 nM). Although its cellular activity was 3.3-fold higher than that of **A1**, its EC_50_ value did not improve compared to **ONC201**. Further modifications, including the introduction of a carbonyl group to **A1** (yielding **A3**) and the inversion of the amide bond (yielding **A4**), similarly failed to enhance the biological activity. To further improve potency, we applied a ring contraction strategy to **A1**, transforming the right-side pyrimidinone into a pyrazolidinone scaffold. The resulting compound **A5** (EC_50_ = 2960.5 nM, IC_50_ = 634.9 nM) exhibited a 4.4-fold increase in cellular activity relative to **A1**. We next investigated the influence of substituents in the blue region by attaching methyl and ethyl groups to the exposed nitrogen atom of **A5**, yielding **A6** and **A7**, respectively. However, neither compound displayed appreciable ClpP activator activity. Our group previously demonstrated that introducing an *R*-methyl at the 5-position of the imipridone enhanced both activity and pharmacokinetic properties, exhibiting a “magic methyl” effect [28]. According to this pattern, we modified the corresponding position on **A5** to afford compound **A8** (EC_50_ = 3839.5 nM, IC_50_ = 938.5 nM). While **A8** exhibited reduced ClpP-activating and cellular antiproliferative potency relative to **A5**, both compounds fell within comparable micromolar potency ranges. In addition, the introduced R-methyl substituent may confer potential favorable pharmacokinetic advantages, as observed in our previous work, which could counterbalance this minor potency penalty. Balancing these trade-offs against our group’s sustained research interest in chiral moieties at this position, we selected the piperidine-fused imidazolone derivative of **A8** as the optimal scaffold for subsequent side-arm optimization.

With the optimal scaffold identified, we optimized the right side-arm (Table 2). Replacing the phenyl ring with a cyclohexane (**A9**, EC_50_ = 4092 nM) resulted in a marked reduction in enzymatic activity, indicating that the aromaticity of the right side-arm is crucial. The methyl substituent position on the phenyl ring significantly affected activity: the *para*-substituted compound **A11** (EC_50_ = 423.9 nM, IC_50_ = 229.4 nM) was more effective than the *meta-*substituted **A10** (EC_50_ = 544.6 nM, IC_50_ = 321.2 nM), demonstrating that *para* substitution is preferred over *ortho* or *meta*. Introducing a methoxy group at this position (**A12**, EC_50_ = 463.7 nM, IC_50_ = 327.0 nM) maintained comparable potency; however, incorporating a bulky *tert*-butyl group (**A13**) caused a dramatic loss in activity, with both EC_50_ and IC_50_ values exceeding 2000 nM. This shows that the binding pocket has limited space at the *para*-position. Furthermore, compound **A14**, bearing a flat aromatic fused-ring system, exhibited an excellent IC_50_ of 135.1 nM, which was superior to that of **ONC201**. We then tested different halogen-substituted derivatives at the *para-*position, including **A15** (EC_50_ = 1096.5 nM, IC_50_ = 129.1 nM), **A16** (EC_50_ = 32.7 nM, IC_50_ = 13.3 nM), and **A17** (EC_50_ = 126.5 nM, IC_50_ = 38.6 nM). Notably, **A16** displayed exceptional ClpP activation and cellular inhibitory efficacy, achieving an approximately 26.8-fold improvement in enzymatic activity and a 20-fold enhancement in cellular potency compared to **ONC201**. Replacing the bromo substituent with a -CF_3_ group afforded **A18** (EC_50_ = 59.7 nM, IC_50_ = 41.0 nM), which similarly demonstrated outstanding potency. Introduction of 4-OCF_3_ and 4-OCHF_2_ groups maintained moderate activity, though slightly diminished relative to the 4-CF_3_ analogue. Meanwhile, exploring multi-fluoro substitutions showed that the 3,4-difluoro derivative **A24** (EC_50_ = 334.6 nM) was superior to other difluoro isomers, but still less active than **A16**. Furthermore, **A16** exhibited the expected magic methyl effect, showing a 33-fold increase in enzymatic activity and a 16-fold increase in antiproliferative activity compared to compound **S1**, which lacks the corresponding chiral methyl group (Table S1, Figure S2). Based on these SAR results, we selected the right side-arm of **A16** for further structural optimization.

After completing the structural optimization on the right-side moieties, we proceeded to optimize the left-side moieties. Subsequently, we explored monosubstituted phenyl derivatives (**A27**–**A34**). The introduction of a fluorine atom at the *ortho*-, *meta*-, and *para*-positions of the left phenyl ring revealed that *meta*-substitution is more favorable than *ortho*- or *para*-substitution. Subsequent introduction of larger halogens at the *meta*-position gave **A30** (EC_50_ = 11.7 nM, IC_50_ = 4.6 nM) and **A31** (EC_50_ = 13.8 nM, IC_50_ = 3.9 nM); both compounds exhibited remarkable tumor cell inhibitory activities. Furthermore, the introduction of a weakly electron-donating methyl group (**A32**, EC_50_ = 5.6 nM, IC_50_ = 4.8 nM) maintained a strong activity. Substituting the position with *meta*-trifluoromethyl group afforded **A33** (EC_50_ = 2.8 nM, IC_50_ = 1.7 nM), which retained excellent enzymatic and cellular potency; however, introducing a *meta*-trifluoromethoxy (**A34**, EC_50_ = 10.1 nM, IC_50_ = 82.1 nM) substituent led to reduced cellular efficacy. Introducing two substituents onto the phenyl ring led to notable improvements. The 3,5-difluoro substituted compound **A36** (EC_50_ = 6.02 nM, IC_50_ = 1.13 nM) demonstrated exceptional potency in both assays. Compared to **ONC201**, the enzymatic and cellular activities of **A36** were dramatically improved by 146-fold and 240-fold, respectively. By contrast, retaining the *meta*-fluoro substituent while introducing a *para*-cyano group (**A37**, EC_50_ = 1111.0 nM, IC_50_ = 392.3 nM) resulted in a dramatic loss of activity (Table 3). Overall, **A33** and **A36** exhibited comparable activity, but **A36** slightly outperformed **A33** in cell inhibition. Considering the advantage in cell proliferation inhibition activity, **A36** was selected as the optimal compound for further investigation and named it **CLPP-3036**.

### 2.2. Chemistry

**Scheme 1:**
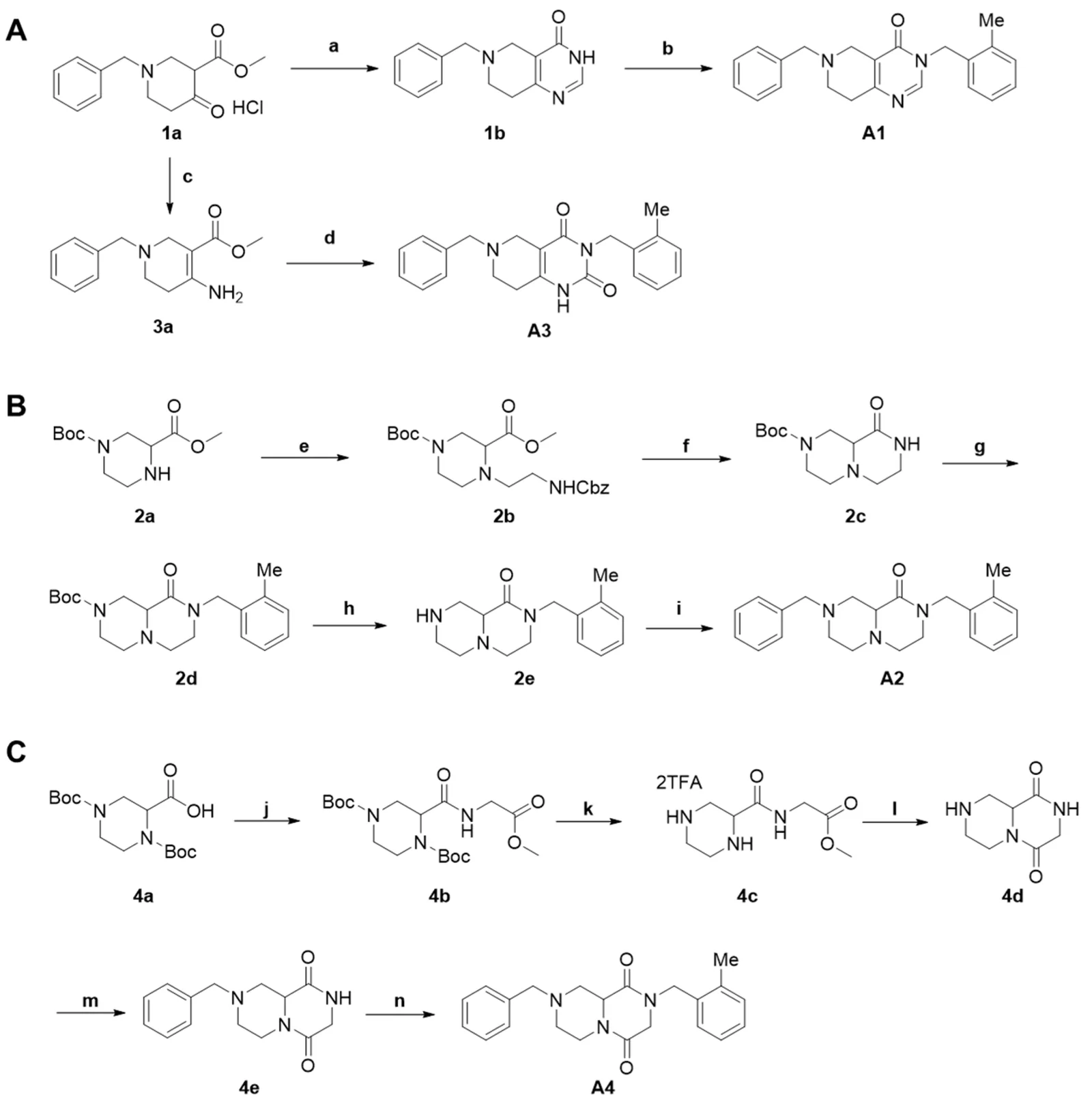
Synthesis of compounds **A1**~**A4**. Reagents and conditions. (**A**): (a) formamidamide hydrochloride, MeONa, MeOH, reflux, overnight, 35% yield; (b) 2-methylbenzyl chloride, Cs_2_CO_3_, THF, 80 °C, 16 h, 7% yield; (c) NH_4_OAc, MeOH, 40 °C, 8 h, 69% yield; (d) (i) 1-(isocyanatomethyl)-2-methylbenzene, TEA, Toluene, 80 °C, 16 h; (ii) MeONa, MeOH, reflux, 16 h, 13% yield; (**B**): Synthesis of compound **A2**. Reagents and conditions: (e) benzyl (2-bromoethyl)carbamate, K_2_CO_3_, DMF, 4 h, 31% yield; (f) (i) Pd/C, H_2_, 2 h. (ii) DIPEA, ACN, 60 °C, 6 h, 75% yield; (g) 2-methylbenzyl chloride, NaH, DMF, 8 h, 56% yield; (h) TFA, DCM, r.t., 2 h, 89% yield; (i) benzaldehyde, NaBH(OAc)_3_, CH_3_COOH, DCM, r.t., overnight, 17% yield; (**C**): (j) glycine methyl ester hydrochloride, HOBT, EDCI, DIPEA, r.t. 4 h, 61% yield; (k) TFA, DCM, r.t., 2 h, 100% yield; (l) DBU, MeOH, r.t., 4 h, 40% yield; (m) benzaldehyde, NaBH(OAc)_3_, TEA, DCM, r.t., overnight, 35% yield; (n) 2-methylbenzyl chloride, NaH, DMF, r.t., 8 h, 14% yield.

Synthesis of compounds **A1** and **A3** is illustrated in Part A of Scheme 1. Commercially available methyl 1-benzyl-4-oxopiperidine-3-carboxylate hydrochloride **1a** reacted with formamidine hydrochloride to afford intermediate **1b**. Subsequent alkylation of **1b** with 2-methylbenzyl chloride using cesium carbonate as the base yielded **A1**. Meanwhile, **1a** underwent a substitution reaction with ammonium acetate, followed by a cyclization reaction with 1-(isocyanatomethyl)-2-methylbenzene to give **A3**. Synthesis of compound **A2** is outlined in Part B of Scheme 1. First, the intermediate **2b** was obtained by reacting commercially available 1-tert-butyl 3-methyl piperazine-1,3-dicarboxylate **2a** with benzyl (2-bromoethyl)carbamate in the presence of K_2_CO_3_. Then, **2b** underwent hydrogenation with Pd/C under H_2_, followed by an intramolecular amine-ester exchange reaction in the presence of DIPEA to give **2c**. Compound **2d** was then obtained through further reaction of **2c** with 2-methylbenzyl chloride in the presence of NaH. In the presence of trifluoroacetic acid, **2d** was deprotected to give **2e**. Subsequently, **2e** underwent reductive amination reaction with benzaldehyde in the presence of NaBH(OAc)_3_ and DIPEA to obtain **A2**. Synthesis of compound **A4** is illustrated in Part C of Scheme 1. First, commercially available 1,4-Di-Boc-piperazine-2-carboxylic acid **4a** underwent amide condensation with glycine methyl ester hydrochloride to afford intermediate **4b**. In the presence of trifluoroacetic acid, **4b** was deprotected to give **4c**. Subsequently, in the presence of DBU, compound **4c** underwent intramolecular cyclization to obtain **4d**. Then, **4d** reacted with benzaldehyde via reductive amination catalyzed by triethylamine, to afford **4e**. Finally, **4e** reacted with 2-methylbenzyl chloride in the presence of NaH to give **A4**.

**Scheme 2:**
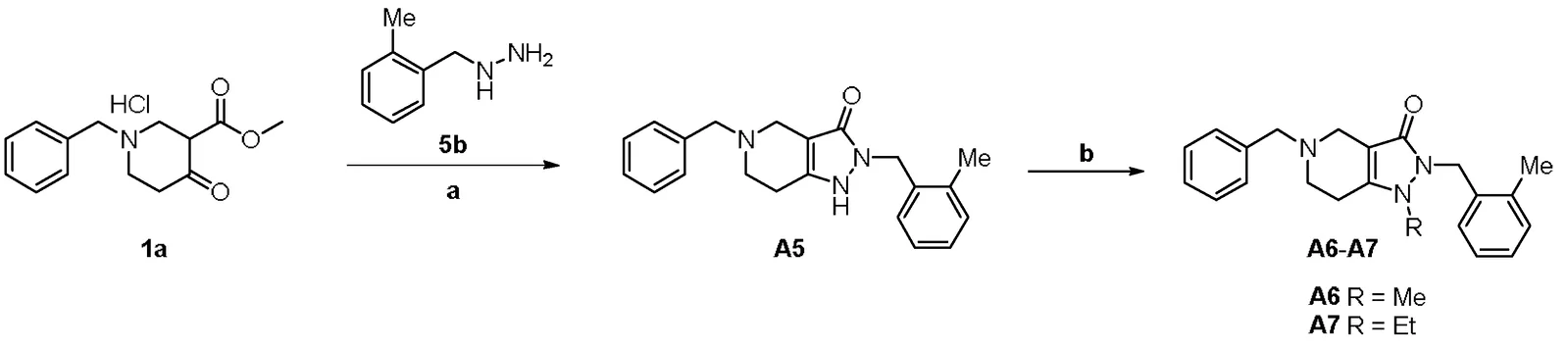
Synthesis of compounds **A5**–**A7**. Reagents and conditions: (a) CH_3_COONa, EtOH, r.t., 6 h, 54% yield; (b) RI, Cs_2_CO_3_, DMF, 80 °C, 16 h, 6–10% yield.

Synthesis of compounds **A5**–**A7** is shown in Scheme 2. First, commercially available methyl 1-benzyl-4-oxopiperidine-3-carboxylate hydrochloride **1a** cyclized with (2-methylbenzyl)hydrazine **5b** in the presence of CH_3_COONa to obtain **A5**. Subsequently, **A5** under a substitution reaction with alkyl iodide in the presence of Cs_2_CO_3_ to give **A6**–**A7**.

**Scheme 3:**
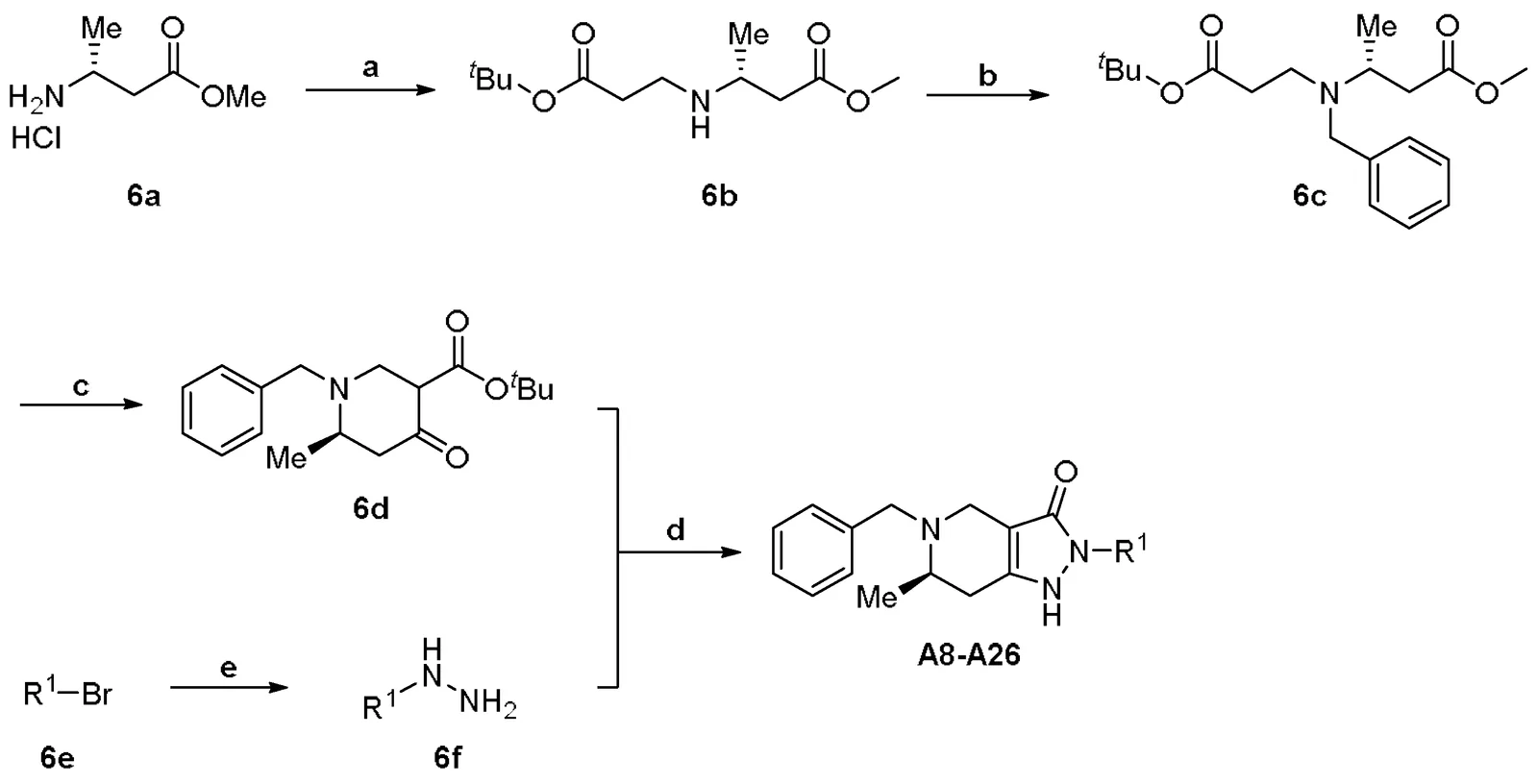
Synthesis of compounds **A8**–**A26**. (a) tert-Butyl acrylate, DBU, TEA, DCM, 40 °C, 24 h, 89% yield; (b) BnBr, TEA, DCM, r.t., 24 h, 72% yield; (c) *t-*BuOK, THF, −70 °C, 4 h, 46% yield; (d) CH_3_COONa, EtOH, r.t., overnight; MeONa, 6 h, 15–20% yield; (e) N_2_H_4_·H_2_O, MeOH, r.t., 6 h, 60–75% yield.

Synthesis of compounds **A8**–**A26** is outlined in Scheme 3. The synthesis of **6d** can be referred to our previous work [28]. Meanwhile, hydrazine hydrate reacted with different substituted benzyl bromides **6e** to obtain the corresponding benzylhydrazines **6f**. Subsequently, **6d** reacted with substituted benzylhydrazines **6f** in the presence of MeONa to give **A8**–**A26**.

**Scheme 4:**
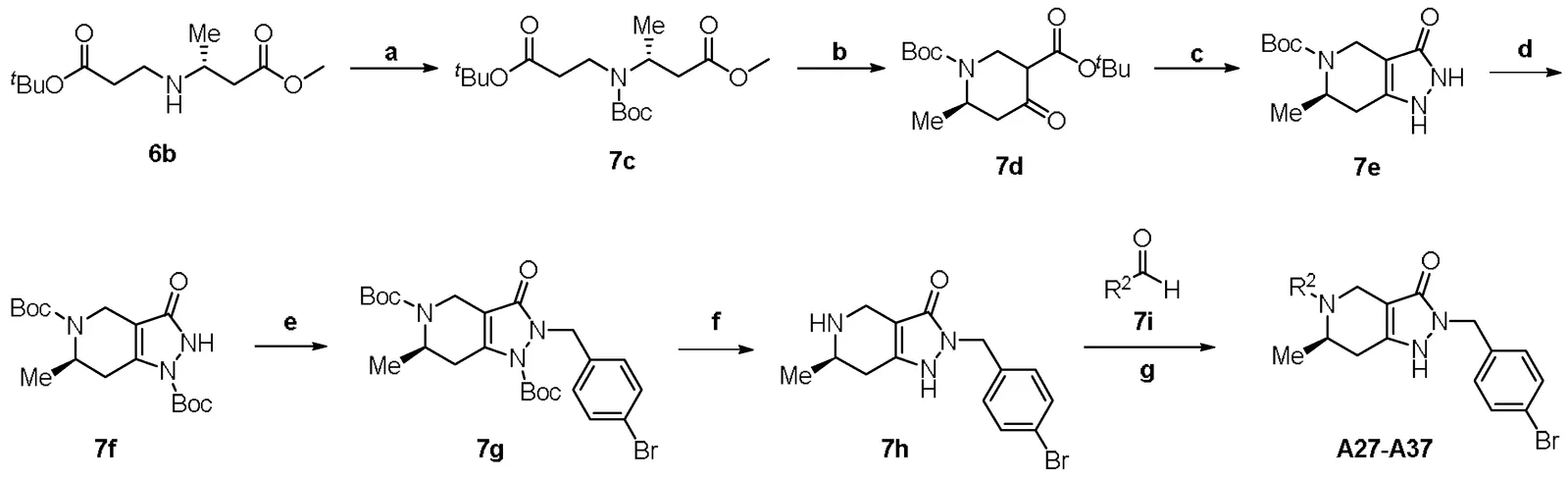
Synthesis of compounds **A27**–**A37**. Reagents and conditions: (a) Boc_2_O, TEA, DCM, r.t., 8 h, 93% yield; (b) *t-*BuOK, THF, −70 °C, 4 h, 36% yield; (c) N_2_H_4_·H_2_O, EtOH, reflux, 2 h, 90% yield; (d) Boc_2_O, TEA, DMAP, DCM, r.t., 8 h, 88% yield; (e) NaH, DMF, 4-bromobenzyl bromide, r.t. 6 h, 83% yield; (f) TFA, DCM, 40 °C, 2 h, 80% yield; (g) NaBH(OAc)_3_, DIPEA, DCM, r.t., 12 h, 5–10% yield.

Synthesis of compounds **A27**–**A37** is summarized in Scheme 4. The synthesis of **7d** can be referred to our previous work [28]. Compound **7e** was obtained by reacting **7d** with hydrazine hydrate. Then, **7e** reacted with (Boc)_2_O, followed by a nucleophilic substitution reaction with 3,4-difluorobenzyl bromide to give **7g**. In the presence of trifluoroacetic acid, **7g** was deprotected to give **7h**. Subsequently, **7h** reacted with different substituted benzaldehydes **7i** via reductive amination to obtain **A27**–**A37**.

### 2.3. Biological Activity

**Figure 4:**
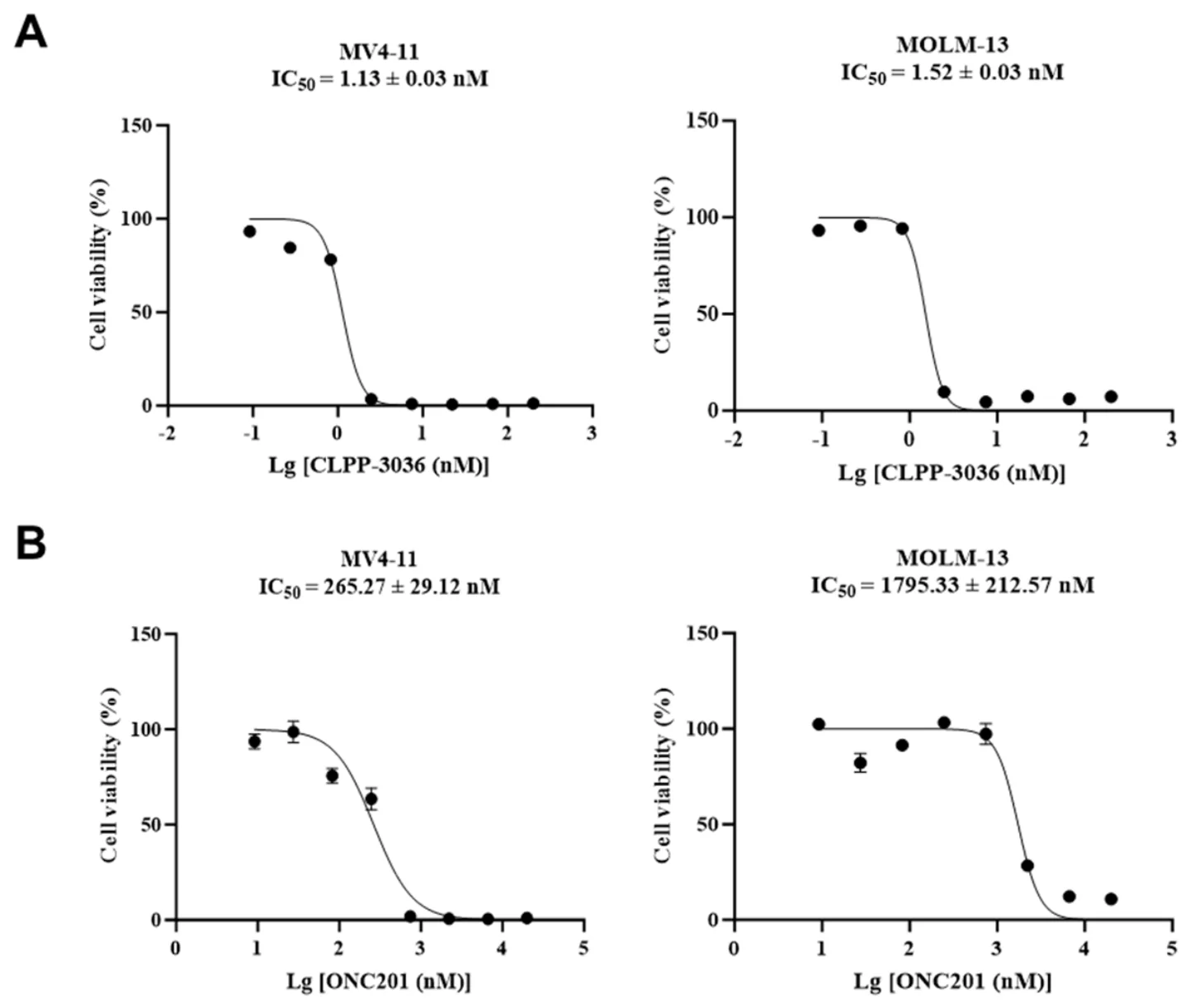
(**A**) Viability curves of MV4-11 and MOLM-13 cells treated with **CLPP-3036** at indicated concentrations for 72 h. (**B**) Viability curves of MV4-11 and MOLM-13 cells treated with **ONC201** at indicated concentrations for 72 h.

To further investigate compound **CLPP-3036**, we subsequently evaluated its anti-proliferative activity against multiple tumor cell lines, together with **ONC201** (Table 4). The results showed that the IC_50_ values of **CLPP-3036** against both hematological malignant tumor cells and solid tumor cells reached or approached the single-digit nanomolar level. In the MV4-11 and MOLM-13 AML tumor cell lines, which were previously used in studies to evaluate the cellular activity of ClpP activators, the IC_50_ values of **CLPP-3036** were 1.13 nM and 1.52 nM, respectively, representing 235-fold and 1180-fold improvements compared to **ONC201** (Figure 4). In the B-cell lymphoma Raji cell line, the IC_50_ of **CLPP-3036** was 9.8 nM, with a 1260-fold improvement compared to **ONC201**. **CLPP-3036** also demonstrated clear advantages in various solid tumor models (Table 4). In the human melanoma A375 cell line, we observed the IC_50_ values of 6.8 nM and 1156.3 nM for **CLPP-3036** and **ONC201**, respectively (approximately 169-fold improvement). In the pancreatic ductal adenocarcinoma Mia PaCa-2 and non-small cell lung cancer H1299 cell lines, the activity of **CLPP-3036** was improved by 516-fold and 513-fold compared to **ONC201**, respectively. These results demonstrated that across tested cell lines, compound **CLPP-3036** exhibited a potent antiproliferative effect.

**Figure 5:**
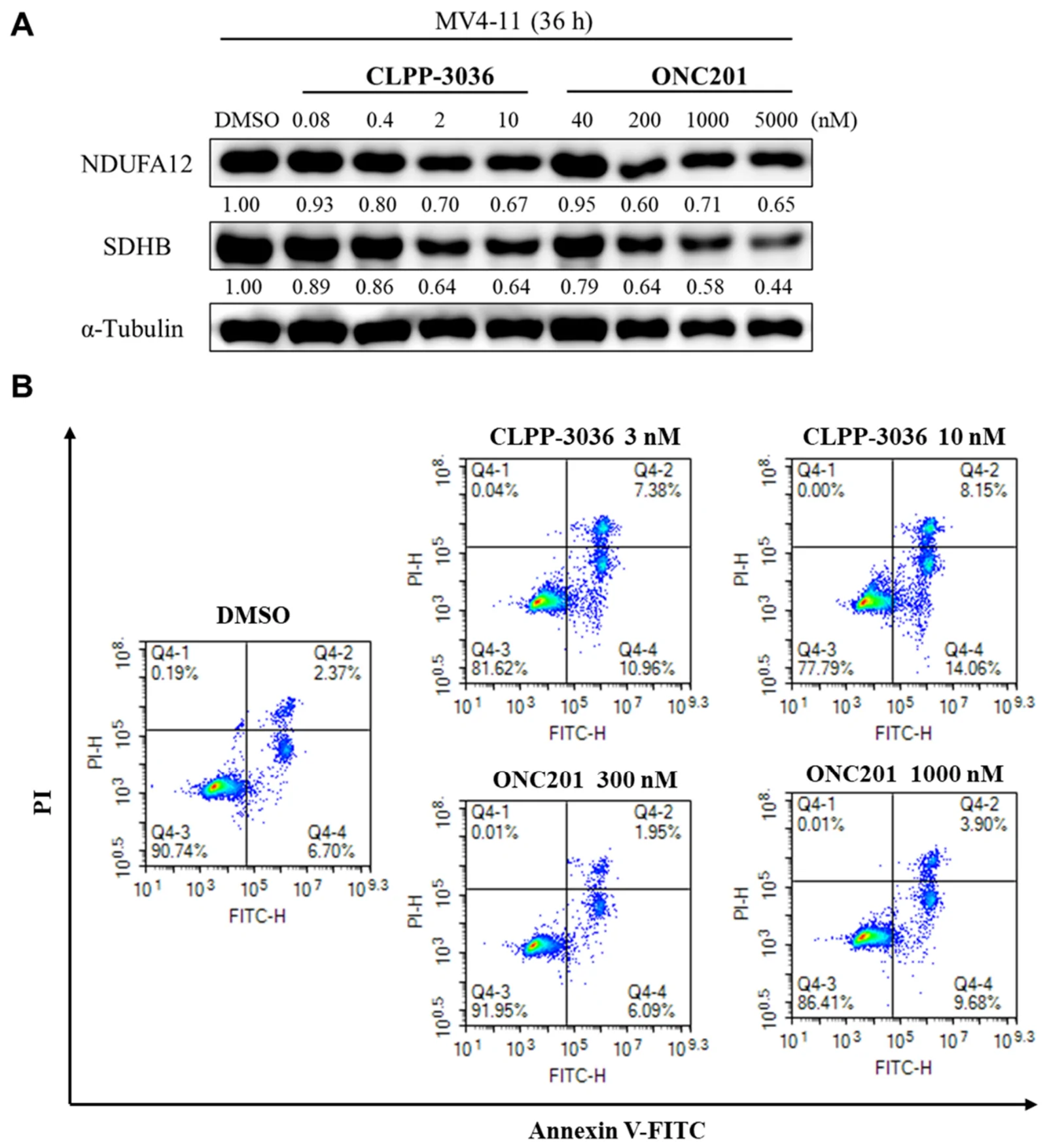
(**A**) Western blotting analysis of the degradation levels of SDHB and NDUFA12 in MV4-11 treated with **CLPP-3036** for 36 h at the indicated concentrations. (**B**) Apoptosis analysis of the MV4-11 cells treated with **CLPP-3036** for 36 h.

ClpP activators are known to be able to induce degradation of mitochondrial proteins; therefore, we examined the impact of **CLPP-3036** on two established ClpP representative substrates SDHB and NDUFA12 in MV4-11 cells. Since the degradation levels of NDUFA12 and SDHB correlate positively with the cellular activity of ClpP activators, and **CLPP-3036** exhibits markedly stronger agonistic activity than **ONC201**, a lower initial dosing concentration was applied to **CLPP-3036** in the experiment. As shown in Figure 5A: after treating MV4-11 cells with different concentrations of **CLPP-3036** for 36 h, Western blot results demonstrated that treatment with 2 nM **CLPP-3036** markedly downregulated the protein levels of SDHB and NDUFA12. In comparison, a concentration of 200 nM **ONC201** was required to achieve obvious degradation of these two proteins. These findings suggest that CLPP-3036 possesses stronger ClpP-activating activity than **ONC201**. Furthermore, **CLPP-3036** and **ONC201** have been evaluated for their ability to induce apoptosis in the MV4-11 cell line by flow cytometry (Figure 5B). The results showed that **CLPP-3036** triggered robust apoptosis in this cell line. Treatment with **CLPP-3036** for 36 h induced apoptosis in MV4-11 cells in a dose-dependent manner, with apoptotic proportions reaching 18.34% at 3 nM and 22.21% at 10 nM. In contrast, **ONC201** triggered substantially lower levels of apoptosis at much higher concentrations, with only 8.04% at 300 nM and 13.58% at 1000 nM. These findings indicate that the antitumor efficacy of CLPP-3036 is at least partially attributed to its potent pro-apoptotic activity.

### 2.4. Binding Mode Analysis of CLPP-3036 in ClpP

**Figure 6:**
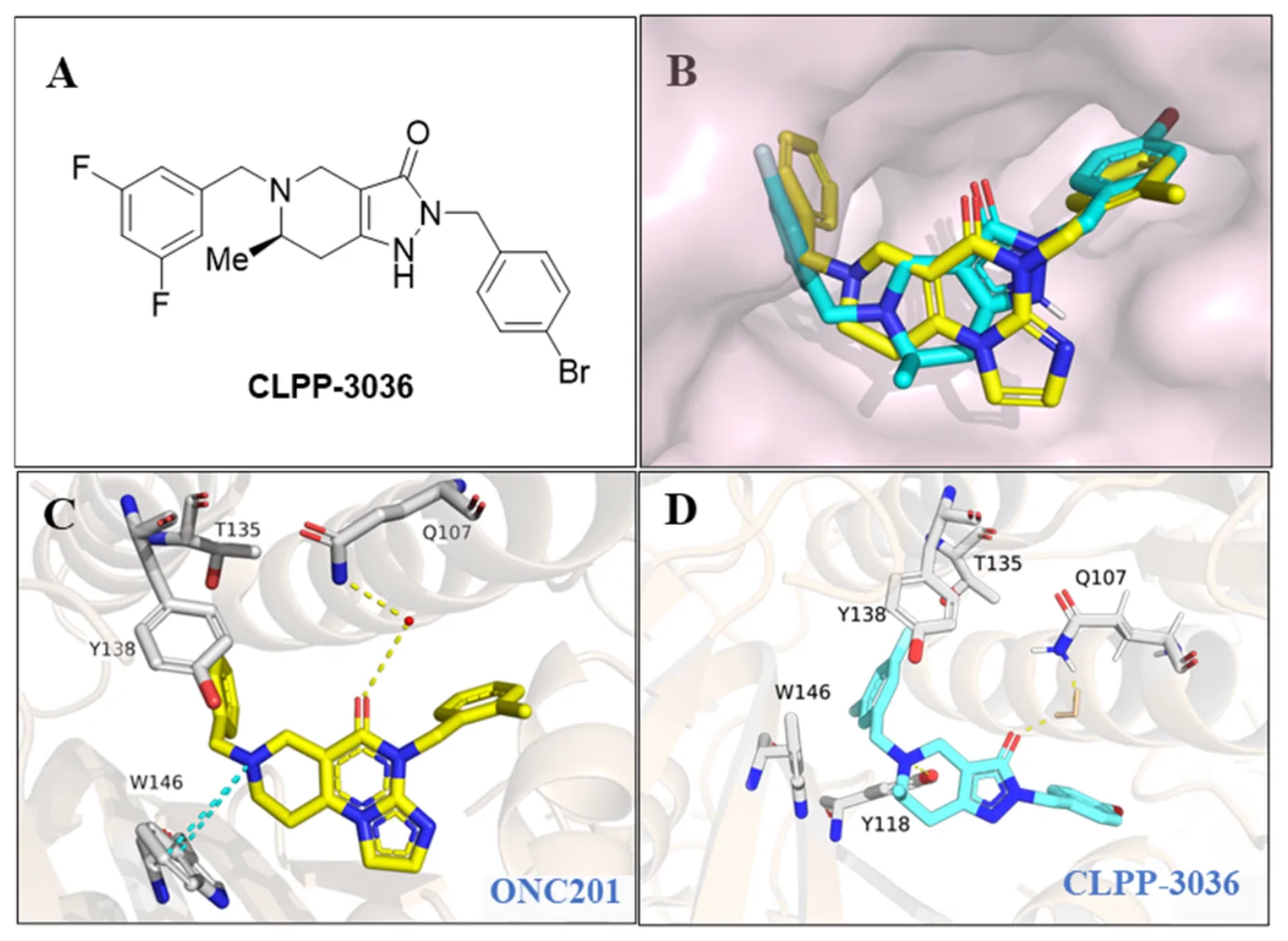
Predicted binding mode of **CLPP-3036** in ClpP. (**A**) Chemical structure of **CLPP-3036**. (**B**) Overall view of **CLPP-3036** (blue) and **ONC201** (yellow) in the ClpP activator-binding pocket, RMSD = 0.115. (**C**) Binding interactions of **ONC201** in ClpP. (**D**) Docking-predicted binding interactions of **CLPP-3036** in ClpP (Docking score = 8.19). Key residues are shown as sticks, and hydrogen bonds are indicated by dashed lines.

To illustrate the improved activity of the piperidine-fused imidazolone activator **CLPP-3036**, we predicted its binding mode in the ClpP activator-binding pocket by molecular docking (Figure 6). The predicted binding hypothesis suggests that the pose of **CLPP-3036** exhibits deeper extension of its two aryl side arms into the hydrophobic subpockets compared to **ONC201**, leading to more extensive contacts with the pocket-lining residues (Figure 6B). The imidazolone moiety of **CLPP-3036** is predicted to remain near the Q107-centered polar region, enabling water-mediated hydrogen-bond interactions with Q107 (Figure 6C,D). In addition, the aryl groups of **CLPP-3036** are predicted to form favorable aromatic contacts with Y138 and W146, which may contribute to stabilizing the activator-bound conformation. Furthermore, the tertiary amine moiety of **CLPP-3036** is predicted to form hydrophobic contacts with Y118. The stereochemically defined methyl substituent further projects toward the W146-associated hydrophobic subpocket, potentially enhancing local hydrophobic packing. Collectively, these predicted interactions provided a structural rationale for the enhanced improved ClpP-activating potency of **CLPP-3036**.

## 3. Material and Methods

### 3.1. Chemical Synthesis

General information: starting materials, reagents and solvents were purchased from Bide Pharmatech (Shanghai, China), Adamas-beta (Shanghai, China), Energy Chemical (Shanghai, China), and J&K (Beijing, China), and were used without further purification. Flash Chromatography was performed on a Biotage Isolera One (Biotage AB publ, Uppsala, Sweden) using a Sfär Bio C18 column (Biotage AB publ, Uppsala, Sweden). Nuclear magnetic resonance (NMR) spectroscopy was performed on a Bruker Ascend 500 or 600 MHz NMR instrument (IS as TMS). Chemical shifts were reported in parts per million (ppm, δ) downfield from tetramethylsilane. Proton coupling patterns were described as singlet (s), doublet (d), triplet (t), quartet (q), multiplet (m), and broad (br). High-resolution mass spectra (HRMS) and low-resolution mass spectra (LRMS) were obtained by electrospray ionization (ESI) using Thermo Exactive Plus (Thermo Fisher Scientific, Waltham, MA, USA) and Agilent 6125C (Agilent Technologies Inc., Santa Clara, CA, USA), respectively. HPLC data analysis of compounds was performed on an Agilent 1290 (Agilent Technologies Inc., Santa Clara, CA, USA) with a quaternary pump and diode-array detector (DAD), the peak purity was verified by UV spectra. All compounds are >95% pure by HPLC analysis. The NMR and HRMS, HPLC traces are contained in Supplementary Materials.

*benzyl-3-(2-methylbenzyl)-5,6,7,8-tetrahydropyrido[4,3-d]pyrimidin-4(3H)-on* (**A1**). A mixture of **1a** (2.00 g, 7.00 mmol), formamidine hydrochloride (471 mg, 5.80 mmol), and MeONa (626 mg, 11.00 mmol) in MeOH (20 mL) was stirred overnight at 80 °C. The residue was diluted with H_2_O (20 mL) and extracted with EA (3 × 20 mL). The combined organic layers were washed with brine, dried over Na_2_SO_4_, and concentrated. The residue was purified by silica gel chromatography (PE:EA = 3:1) to afford intermediate **1b** as a yellow solid (600 mg, 35% yield). LC/MS (ESI): *m*/*z* 242.2 [M + H]^+^. Then, a solution of **1b** (300 mg, 1.25 mmol), 2-methylbenzyl chloride (180 μL, 1.36 mmol), and Cs_2_CO_3_ (604 mg, 1.86 mmol) in THF (15 mL) was stirred for overnight at 80 °C. The mixture was diluted with H_2_O (20 mL) and extracted with EA (3 × 20 mL). The combined organic layers were washed with brine, dried over Na_2_SO_4_, and concentrated. The residue was purified via silica gel chromatography to afford the final compound **A1** as a yellow powder (30 mg, 7% yield). ^1^H NMR (500 MHz, DMSO-*d*_6_) δ 8.37 (s, 1H), 7.34 (t, *J* = 3.7 Hz, 4H), 7.30–7.24 (m, 1H), 7.21–7.12 (m, 3H), 6.82 (d, *J* = 7.6 Hz, 1H), 5.06 (d, *J* = 2.6 Hz, 2H), 3.68 (s, 2H), 3.33 (s, 1H), 2.75–2.63 (m, 4H), 2.33 (s, 3H). ^13^C NMR (151 MHz, DMSO-*d*_6_) δ 163.76, 159.54, 158.48, 149.87, 138.31, 136.05, 134.80, 130.69, 129.34, 128.73, 127.88, 127.60, 126.81, 126.55, 119.43, 61.88, 56.56, 49.43, 49.35, 46.97, 31.44, 19.23; HRMS (ESI) calcd for C_22_H_24_N_3_O^+^ 346.1913 [M + H]^+^, found 346.1912.
*1-(tert-butyl)-3-methyl-4-(2-(((benzyloxy)carbonyl)amino)ethyl)piperazine-1,3-dicarboxylate* (**2b**). A mixture of methyl 1-Boc-piperazine-3-carboxylate **2a** (2.00 g, 8.2 mmol), benzyl (2-bromoethyl)carbamate (2.32 g, 9.0 mmol), and K_2_CO_3_ (1.36 g, 9.80 mmol) in DMF (10 mL) was stirred for overnight at 60 °C. After the reaction was completed, the mixture was diluted with H_2_O (20 mL) and extracted with EA (3 × 20 mL). The combined organic layers were washed with brine, dried over Na_2_SO_4_, filtered, and concentrated under reduced pressure. The residue was purified via silica gel chromatography (PE:EA = 1:1) to afford the title compound **2b** (1.10 g, 31% yield) as a yellow solid.
*tert-butyl-9-oxooctahydro-2H-pyrazino[1,2-a]pyrazine-2-carboxylate* (**2c**). The previously obtained intermediate **2b** (1.10 g, 2.50 mmol) was dissolved in MeOH (10 mL). After purging with hydrogen three times, 10% Pd/C (500 mg) was added, and the mixture was stirred at room temperature for 2 h. After the reaction was completed, the catalyst was removed by filtration through a pad of Celite. The filtrate was concentrated in vacuo, dissolved in ACN (10 mL), and DIPEA (435 μL, 2.50 mmol) was added. The crude product was purified by reversed-phase chromatography to afford the title compound **2c** (500 mg, 75% yield). LC/MS (ESI, *m*/*z*): 255.2 [M + H]^+^.
*1-(2-methylbenzyl)hexahydro-2H-pyrazino[1,2-a]pyrazin-1(6H)-one* (**2e**). The aforementioned intermediate **2c** (500 mg, 1.96 mmol) was dissolved in DMF (5 mL), and NaH (150 mg, 3.92 mmol) was slowly added. The mixture was stirred at room temperature for 1h. Subsequently, 1-(chloromethyl)-2-methylbenzene (300 mg, 2.15 mmol) was added, and the reaction was stirred at room temperature for an additional 8h. The reaction was quenched with H_2_O (20 mL) and extracted with EA (3 × 20 mL). The combined organic layers were dried over Na_2_SO_4_ and concentrated in vacuo to afford the crude compound **2d** (395 mg, 56% yield). The crude product **2d** was dissolved in DCM (2 mL), followed by the addition of TFA (1 mL). After the reaction was completed, the solvent was removed under reduced pressure to afford the title compound **2e** (350 mg, 89% yield) as a yellow solid.
*6-benzyl-2-(2-methylbenzyl)hexahydro-2H-pyrazino[1,2-a]pyrazin-1(6H)-one* (**A2**). The obtained compound **2e** (350 mg, 0.98 mmol), benzaldehyde (168 μL, 1.43 mmol), and CH_3_COOH (112 μL, 1.96 mmol) were dissolved in DCM (25 mL). Activated 4Å molecular sieves were added, and the mixture was stirred at room temperature for 1 h under a nitrogen atmosphere. Then, sodium triacetoxyborohydride (415 mg, 1.96 mmol) was added, and the reaction was stirred at room temperature overnight. After the reaction was completed, the molecular sieves were removed by filtration through a pad of Celite. The filtrate was diluted with H_2_O (20 mL) and extracted with DCM (3 × 20 mL). The combined organic layers were concentrated in vacuo, and purified by silica gel chromatography (PE:EA = 2:1) to afford the final compound **A2** (80 mg, 17% yield) as a white solid. ^1^H NMR (500 MHz, DMSO-*d*_6_) δ 7.37–7.28 (m, 4H), 7.26 (ddd, *J* = 8.7, 5.5, 2.3 Hz, 1H), 7.19–7.14 (m, 3H), 7.07–7.03 (m, 1H), 4.49 (d, *J* = 3.5 Hz, 2H), 3.56 (d, *J* = 13.2 Hz, 1H), 3.48 (d, *J* = 13.1 Hz, 1H), 3.28–3.25 (m, 1H), 2.98 (dd, *J* = 11.7, 4.0 Hz, 1H), 2.86 (dd, *J* = 11.7, 4.9 Hz, 1H), 2.78 (dt, *J* = 10.8, 2.5 Hz, 1H), 2.73 (dd, *J* = 10.6, 3.1 Hz, 2H), 2.43 (td, *J* = 11.9, 4.3 Hz, 1H), 2.24 (td, *J* = 11.0, 2.7 Hz, 1H), 2.21 (s, 3H), 2.14 (td, *J* = 11.1, 2.7 Hz, 1H), 1.93–1.86 (m, 1H). ^13^C NMR (151 MHz, DMSO-*d*_6_) δ 166.64, 138.60, 136.48, 134.88, 130.72, 129.30, 128.66, 127.65, 127.57, 127.44, 126.37, 63.97, 62.47, 54.36, 53.98, 52.50, 50.33, 47.19, 45.85, 19.16. HRMS (ESI) calcd for C_22_H_28_N_3_O^+^ 350.2226 [M + H]^+^, found 350.2225.
*6-benzyl-3-(2-methylbenzyl)-5,6,7,8-tetrahydropyrido[4,3-d]pyrimidine-2,4(1H,3H)-dione* (**A3**). Intermediate **1a** (5.00 g, 17.00 mmol) and NH_4_OAc (6.70 g, 87.00 mmol) were dissolved in MeOH (50 mL), and the mixture was stirred at reflux overnight. Upon completion, the reaction mixture was cooled to room temperature, and the solvent was removed in vacuo. The residue was diluted with H_2_O (30 mL) and extracted with EA (3 × 20 mL). The combined organic layers were washed with brine, dried over Na_2_SO_4_, and concentrated under reduced pressure. The crude product was purified by silica gel chromatography (PE:EA = 2:1) to afford intermediate **3a** (3.00 g, 69% yield) as a yellow solid. LC/MS (ESI, *m*/*z*): 247.3 [M + H]^+^. A mixture of **3a** (300 mg, 1.22 mmol) and 1-(isocyanatomethyl)-2-methylbenzene (190 μL, 1.50 mmol) was dissolved in toluene (10 mL), and triethylamine (180 μL, 1.80 mmol) was added dropwise. The reaction was stirred at 80 °C for 6 h. After the reaction was completed, the solvent was removed in vacuo. The residue was diluted with H_2_O (50 mL) and extracted with EA (3 × 50 mL). The combined organic layers were washed with brine, dried over Na_2_SO_4,_ and concentrated under reduced pressure. The crude product was purified by reversed-phase chromatography to afford the final product **A3** (60 mg, 13% yield). ^1^H NMR (500 MHz, DMSO-*d*_6_) 9.11 (s, 1H), 7.35–7.31 (m, 5H), 7.29–7.23 (m, 1H), 7.19 (d, *J* = 7.5 Hz, 1H), 7.14 (t, *J* = 7.6 Hz, 1H), 7.06 (t, *J* = 7.4 Hz, 1H), 5.01 (s, 2H), 3.60 (s, 2H), 3.58 (s, 2H), 3.11 (s, 2H), 2.97 (t, *J* = 5.9 Hz, 2H), 2.20 (s, 3H). ^13^C NMR (151 MHz, DMSO-*d*_6_) δ 168.18, 163.78, 152.39, 151.83, 138.75, 136.86, 132.35, 130.77, 129.08, 128.74, 127.48, 126.47, 125.62, 125.27, 99.59, 61.90, 51.61, 51.47, 49.17, 29.41, 18.37. HRMS (ESI) calcd for C_22_H_26_N_3_O_3_^+^ 380.1969 [M + H_2_O + H]^+^, found 380.1967.
*methyl (piperazine-2-carbonyl) glycinate* (**4c**). To a solution of **4a** (10.00 g, 30.30 mmol), glycine methyl ester hydrochloride (2.76 g, 31.00 mmol), HOBt (4.20 g, 31.00 mmol), and EDCI (5.94 g, 31.0 mmol) in DCM (20 mL), DIPEA (6.00 g, 46.50 mmol) was added, and the mixture was stirred at room temperature. Upon completion, the reaction mixture was filtered and concentrated under reduced pressure to afford the crude intermediate **4b** (7.32 g, 61% yield). Then the compound **4b** (7.32 g, 18.3 mmol) was dissolved in DCM (20 mL), followed by the addition of TFA (10 mL). The reaction mixture was stirred at reflux. After completion, the solvent was removed in vacuo to afford the title compound **4c** (4.62 g, 100% yield) as a yellow solid.
*hexahydro-2H-pyrazino[1,2-a]pyrazine-6,9-dione* (**4d**). To a solution of compound **4c** (4.50 g, 22.39 mmol) in MeOH (10 mL) was added DBU (10.22 g, 67.16 mmol). The reaction mixture was stirred at room temperature for 2 h, and then concentrated in vacuo. The residue was purified via silica gel chromatography (DCM:MeOH = 10:1) to afford the title compound **4d** (1.50 g, 40% yield).
*2-benzyl-8-(2-methylbenzyl)hexahydro-2H-pyrazino[1,2-a]pyrazine-6,9-dione* (**A4**). Following the procedure described for **A2**, the obtained compound (**4d**, 1.04 g, 6.00 mmol), benzaldehyde (674 μL, 6.60 mmol), and CH_3_COOH (686 μL, 12.00 mmol) were dissolved in DCM (25 mL) to afford the crude compound **4e** (541 mg, 35% yield). The reaction of **4e** (541 mg, 2.09 mmol) with 2-methylbenzyl chloride (444 mg, 3.16 mmol) afforded the target compound **A4** (105 mg, 14% yield) as a white solid. ^1^H NMR (500 MHz, Chloroform-*d*) δ 7.36–7.30 (m, 4H), 7.30–7.27 (m, 1H), 7.26–7.19 (m, 1H), 7.18 (dd, *J* = 7.5, 5.8 Hz, 2H), 7.12 (d, *J* = 6.8 Hz, 1H), 4.72 (d, *J* = 14.7 Hz, 1H), 4.56 (d, *J* = 14.7 Hz, 1H), 4.47 (ddd, *J* = 13.1, 3.3, 1.7 Hz, 1H), 4.17 (dd, *J* = 11.0, 3.2 Hz, 1H), 3.78 (t, *J* = 1.8 Hz, 2H), 3.67 (d, *J* = 13.1 Hz, 1H), 3.59–3.48 (m, 2H), 2.88–2.74 (m, 2H), 2.28 (s, 3H), 2.11 (t, *J* = 11.2 Hz, 1H), 2.03 (td, *J* = 11.7, 3.3 Hz, 1H). ^13^C NMR (151 MHz, Chloroform-*d*) δ 163.24, 161.76, 137.07, 136.92, 132.34, 130.93, 129.31, 129.11, 128.47, 128.40, 127.49, 126.41, 62.59, 57.36, 56.76, 51.31, 48.29, 47.04, 41.30, 19.27. HRMS (ESI) calcd for C_22_H_26_N_3_O_2_^+^ 364.2019 [M + H]^+^, found 364.2017.
*5-benzyl-2-(2-methylbenzyl)-1,2,4,5,6,7-hexahydro-3H-pyrazolo[4,3-c]pyridin-3-one* (**A5**). A mixture of **1a** (283 mg, 1.00 mmol), 2-methylbenzylhydrazine (136 mg, 1.00 mmol), and sodium acetate (164 mg, 2.00 mmol) in EtOH (5 mL) was stirred at 80 °C overnight. Upon completion, the solvent was removed in vacuo, and the residue was purified by reversed-phase chromatography to afford the compound **A5** (180 mg, 54% yield). ^1^H NMR (500 MHz, DMSO-*d*_6_) δ 11.33 (s, 1H), 7.67 (dd, *J* = 6.6, 3.0 Hz, 2H), 7.50–7.43 (m, 3H), 7.16 (d, *J* = 4.1 Hz, 2H), 7.13–7.08 (m, 4.1 Hz, 1H), 6.88 (d, *J* = 7.5 Hz, 1H), 5.03 (s, 2H), 4.50 (dd, *J* = 13.0, 4.3 Hz, 1H), 4.36 (dd, *J* = 12.9, 6.3 Hz, 1H), 4.07–3.91 (m, 2H), 3.68–3.57 (m, 1H), 3.36–3.26 (mf, 6.1 Hz, 1H), 3.08–2.98 (m, 1H), 2.81 (dt, *J* = 16.7, 4.0 Hz, 1H), 2.30 (s, 3H). ^13^C NMR (151 MHz, DMSO-*d*_6_) δ 150.97, 142.09, 136.07, 134.90, 131.89, 130.61, 130.40, 129.94, 129.23, 128.11, 128.09, 126.43, 91.15, 58.31, 49.19, 47.22, 46.28, 20.33, 19.23. HRMS (ESI) calcd for C_21_H_24_N_3_O^+^ 334.1913 [M + H]^+^, found 334.1914.
*5-benzyl-1-methyl-2-(2-methylbenzyl)-1,2,4,5,6,7-hexahydro-3H-pyrazolo[4,3-c]pyridin-3-one* (**A6**). A mixture of **A5** (333 mg, 1.00 mmol), iodomethane (141 mg, 1.00 mmol), and Cs_2_CO_3_ (650 mg, 2.00 mmol) in DMF (5 mL) was stirred at 80 °C overnight. Upon completion, the reaction mixture was diluted with H_2_O (20 mL) and extracted with EA (3 × 20 mL). The combined organic layers were purified by reversed-phase chromatography to afford the title compound **A6** (35 mg, 10% yield). ^1^H NMR (500 MHz, Chloroform-*d*) 7.35–7.30 (m, 4H), 7.28 (t, *J* = 4.7 Hz, 1H), 7.22–7.14 (m, 4H), 4.92 (d, *J* = 15.3 Hz, 1H), 4.74 (d, *J* = 15.3 Hz, 1H), 3.67–3.61 (m, 2H), 3.53 (d, *J* = 13.2 Hz, 1H), 3.16 (dd, *J* = 10.9, 6.0 Hz, 1H), 3.03–2.98 (m, 1H), 2.62 (td, *J* = 12.7, 5.9 Hz, 1H), 2.35 (s, 3H), 2.10 (td, *J* = 11.4, 3.4 Hz, 1H), 1.97 (d, *J* = 10.9 Hz, 1H), 1.46 (s, 3H). ^13^C NMR (151 MHz, Chloroform-*d*) δ 176.69, 172.96, 165.72, 138.04, 136.29, 134.52, 130.46, 128.74, 128.60, 128.45, 127.74, 127.42, 126.14, 61.47, 59.81, 55.28, 45.51, 27.61, 19.31, 18.24. HRMS (ESI) calcd for C_22_H_26_N_3_O^+^ 348.2070 [M + H]^+^, found 348.2071.
*5-benzyl-1-ethyl-2-(2-methylbenzyl)-1,2,4,5,6,7-hexahydro-3H-pyrazolo[4,3-c]pyridin-3-one* (**A7**). Following the procedure described for **A6**, the target compound **A7** (25 mg, 6% yield) was prepared by replacing iodomethane with iodoethane (155 mg, 1.00 mmol). ^1^H NMR (500 MHz, DMSO-*d*_6_) δ 7.39–7.31 (m, 4H), 7.30–7.25 (m, 1H), 7.19–7.09 (m, 4H), 4.79 (d, *J* = 15.5 Hz, 1H), 4.67 (d, *J* = 15.5 Hz, 1H), 3.65 (d, *J* = 13.3 Hz, 1H), 3.50 (d, *J* = 13.4 Hz, 1H), 3.19–3.07 (m, 1H), 2.87 (dd, *J* = 10.9, 2.0 Hz, 1H), 2.57–2.52 (m, 1H), 2.46–2.38 (m, 1H), 2.28 (s, 3H), 2.26–2.17 (m, 1H), 2.00 (td, *J* = 11.4, 3.6 Hz, 1H), 1.85 (d, *J* = 10.9 Hz, 1H), 1.66 (dq, *J* = 14.6, 7.4 Hz, 1H), 0.59 (t, *J* = 7.5 Hz, 3H). ^13^C NMR (151 MHz, Methanol-*d*_4_) δ 176.65, 165.76, 138.11, 136.03, 134.30, 130.03, 128.57, 128.56, 128.06, 127.50, 127.06, 125.66, 60.77, 59.01, 56.05, 55.08, 44.98, 27.26, 25.69, 18.11. HRMS (ESI) calcd for C_23_H_28_N_3_O^+^ 362.2226 [M + H]^+^, found, 362.2225.
General Procedure for the Preparation of **A8**–**A26.** Intermediate **6d** was prepared by following the procedure described for our previous work [28]. A mixture of intermediate **6d** (303 mg, 1.00 mmol), various substituted intermediates **6f** (2.00 mmol), and sodium acetate (246 mg, 3.00 mmol) in EtOH (10 mL) was stirred at room temperature overnight. Subsequently, MeONa (108 mg, 2.00 mmol) was added, and stirred at room temperature for an additional 5 h. Upon completion, the solvent was removed in vacuo, and the residues was diluted with H_2_O (20 mL) and extracted with EA (3 × 20 mL). The combined organic layers were washed with brine (20 mL), dried over Na_2_SO_4_, and concentrated. The residues were purified by reversed-phase chromatography to afford the target compounds **A8**–**A26** in 15–20% yields.
*(R)-5-benzyl-6-methyl-2-(2-methylbenzyl)-1,2,4,5,6,7-hexahydro-3H-pyrazolo[4,3-c]pyridin-3-one* (**A8**). ^1^H NMR (500 MHz, Methanol-*d*_4_) δ 7.56–7.51 (m, 2H), 7.50–7.42 (m, 3H), 7.18 (d, *J* = 4.5 Hz, 2H), 7.13 (dt, *J* = 8.8, 4.0 Hz, 1H), 6.96 (d, *J* = 7.5 Hz, 1H), 5.04 (d, *J* = 2.4 Hz, 2H), 4.95 (s, 2H), 4.38–4.26 (m, 2H), 3.98–3.82 (m, 3H), 3.10 (dd, *J* = 17.5, 5.5 Hz, 1H), 2.72 (dd, *J* = 17.5, 5.3 Hz, 1H), 2.32 (s, 3H), 1.51 (d, *J* = 6.7 Hz, 3H). ^13^C NMR (126 MHz, Methanol-*d*_4_) δ 155.87, 141.70, 135.80, 134.18, 130.63, 130.50, 130.06, 129.54, 129.01, 127.59, 127.47, 125.82, 90.58, 55.38, 54.80, 46.00, 42.99, 26.34, 17.77, 13.65. HRMS (ESI) calcd for C_22_H_26_N_3_O^+^ 348.2070 [M + H]^+^, found 348.2067.
*(R)-5-benzyl-2-(cyclohexylmethyl)-6-methyl-1,2,4,5,6,7-hexahydro-3H-pyrazolo[4,3-c]pyridin-3-one* (**A9**). ^1^H NMR (500 MHz, Methanol-*d*_4_) δ 7.60–7.54 (m, 2H), 7.47–7.42 (m, 3H), 4.32–4.20 (m, 2H), 3.94–3.77 (m, 3H), 3.68 (d, *J* = 7.4 Hz, 2H), 3.14 (dd, *J* = 17.3, 5.5 Hz, 1H), 2.70 (dd, *J* = 17.4, 5.0 Hz, 1H), 1.86–1.77 (m, 1H), 1.76–1.69 (m, 2H), 1.69–1.64 (m, 1H), 1.63–1.56 (m, 2H), 1.48 (d, *J* = 6.7 Hz, 3H), 1.29–1.16 (m, 3H), 0.99 (qd, *J* = 12.0, 3.4 Hz, 2H). ^13^C NMR (126 MHz, Methanol-*d*_4_) δ 140.82, 131.08, 130.63, 129.18, 128.86, 128.85, 90.60, 54.65, 50.51, 47.09, 42.69, 37.68, 30.15, 26.09, 25.99, 25.39, 13.62. HRMS (ESI) calcd for C_21_H_30_N_3_O^+^ 340.2383 [M + H]^+^, found 340.2385.
*(R)-5-benzyl-6-methyl-2-(3-methylbenzyl)-1,2,4,5,6,7-hexahydro-3H-pyrazolo[4,3-c]pyridin-3-one* (**A10**). ^1^H NMR (500 MHz, Methanol-*d*_4_) δ 7.59 (dd, *J* = 6.5, 2.9 Hz, 2H), 7.42 (dd, *J* = 5.0, 1.9 Hz, 3H), 7.17 (t, *J* = 7.5 Hz, 1H), 7.12–6.98 (m, 3H), 5.02–4.92 (m, 2H), 4.37–4.19 (m, 2H), 3.95 (d, *J* = 14.8 Hz, 1H), 3.90–3.78 (m, 2H), 3.12 (dd, *J* = 17.5, 5.5 Hz, 1H), 2.68 (dd, *J* = 17.5, 5.0 Hz, 1H), 2.27 (s, 3H), 1.46 (d, *J* = 6.6 Hz, 3H). ^13^C NMR (126 MHz, Methanol-*d*_4_) δ 155.28, 141.47, 138.12, 136.51, 130.85, 130.34, 129.39, 128.93, 128.24, 128.14, 128.04, 124.51, 90.22, 54.86, 54.39, 48.09, 42.77, 25.94, 20.06, 13.66. HRMS (ESI) calcd for C_22_H_26_N_3_O^+^ 348.2070 [M + H]^+^, 348.2072.
*(R)-5-benzyl-6-methyl-2-(4-methylbenzyl)-1,2,4,5,6,7-hexahydro-3H-pyrazolo[4,3-c]pyridin-3-one* (**A11**). ^1^H NMR (500 MHz, Methanol-*d*_4_) δ 7.50–7.29 (m, 6H), 7.11 (s, 3H), 5.00–4.89 (m, 2H), 4.06–3.90 (m, 2H), 3.60 (d, *J* = 3.9 Hz, 2H), 3.47 (h, *J* = 6.1 Hz, 1H), 2.86 (dd, *J* = 17.0, 5.4 Hz, 1H), 2.46 (dd, *J* = 16.9, 4.9 Hz, 1H), 2.30 (s, 3H), 1.31 (d, *J* = 6.6 Hz, 3H). ^13^C NMR (126 MHz, Methanol-*d*_4_) δ 159.16, 143.18, 137.08, 133.97, 129.75, 128.81, 128.49, 128.14, 127.17, 127.16, 93.61, 55.68, 53.25, 47.16, 43.12, 27.27, 19.73, 13.31. HRMS (ESI) calcd for C_22_H_26_N_3_O^+^ 348.2070 [M + H]^+^, found 348.2074.
*(R)-5-benzyl-2-(4-methoxybenzyl)-6-methyl-1,2,4,5,6,7-hexahydro-3H-pyrazolo[4,3-c]pyridin-3-one* (**A12**). ^1^H NMR (500 MHz, Methanol-*d*_4_) δ 7.48–7.35 (m, 5H), 7.22–7.14 (m, 2H), 6.84 (d, *J* = 8.4 Hz, 2H), 4.97–4.87 (m, 2H), 4.15–4.02 (m, 2H), 3.74 (s, 3H), 3.71 (d, *J* = 6.2 Hz, 2H), 3.65–3.58 (m, 1H), 2.93 (dd, *J* = 17.2, 5.4 Hz, 1H), 2.54 (dd, *J* = 17.1, 5.0 Hz, 1H), 1.36 (d, *J* = 6.7 Hz, 3H). ^13^C NMR (126 MHz, Methanol-*d*_4_) δ 159.39, 157.25, 142.25, 132.70, 130.15, 130.12, 128.79, 128.72, 128.70, 113.63, 91.89, 55.09, 54.35, 54.11, 47.26, 43.00, 26.75, 13.48. HRMS (ESI) calcd for C_22_H_26_N_3_O_2_^+^ 364.2019 [M + H]^+^, found 364.2020.
*(R)-5-benzyl-2-(4-(tert-butyl)benzyl)-6-methyl-1,2,4,5,6,7-hexahydro-3H-pyrazolo[4,3-c]pyridin-3-one* (**A13**). ^1^H NMR (500 MHz, Methanol-*d*_4_) δ 7.58 (dd, *J* = 6.5, 2.9 Hz, 2H), 7.45–7.40 (m, 3H), 7.33 (d, *J* = 8.0 Hz, 2H), 7.19 (d, *J* = 8.1 Hz, 2H), 4.98 (s, 2H), 4.34–4.22 (m, 2H), 3.95 (d, *J* = 14.8 Hz, 1H), 3.90–3.80 (m, 2H), 3.12 (dd, *J* = 17.5, 5.5 Hz, 1H), 2.68 (dd, *J* = 17.4, 5.0 Hz, 1H), 1.46 (d, *J* = 6.7 Hz, 3H), 1.27 (s, 9H). ^13^C NMR (126 MHz, Methanol-*d*_4_) δ 155.42, 150.56, 141.49, 133.55, 130.81, 130.46, 129.36, 128.92, 127.27, 125.18, 90.39, 54.82, 54.48, 52.98, 42.75, 33.96, 30.36, 25.96, 13.67. HRMS (ESI) calcd for C_25_H_33_N_3_O^+^ 390.2539 [M + H]^+^, found 390.2537.
*(R)-5-benzyl-6-methyl-2-(naphthalen-2-ylmethyl)-1,2,4,5,6,7-hexahydro-3H-pyrazolo[4,3-c]pyridin-3-one* (**A14**). ^1^H NMR (500 MHz, Methanol-*d*_4_) δ 7.83–7.73 (m, 3H), 7.65 (s, 1H), 7.47–7.30 (m, 8H), 5.20–5.07 (m, 2H), 4.04–3.91 (m, 2H), 3.63 (s, 2H), 3.42 (h, *J* = 6.2 Hz, 1H), 2.81 (dd, *J* = 17.0, 5.4 Hz, 1H), 2.44 (dd, *J* = 17.0, 5.2 Hz, 1H), 1.28 (d, *J* = 6.6 Hz, 3H). ^13^C NMR (126 MHz, Methanol-*d*_4_) δ 158.60, 142.96, 134.67, 133.83, 133.39, 132.92, 129.91, 128.58, 128.41, 128.04, 127.43, 127.33, 125.99, 125.97, 125.69, 125.15, 92.28, 55.25, 53.70, 47.73, 43.32, 27.17, 13.44. HRMS (ESI) calcd for C_25_H_26_N_3_O^+^ 384.2070 [M + H]^+^, found 384.2073.
*(R)-5-benzyl-2-(4-fluorobenzyl)-6-methyl-1,2,4,5,6,7-hexahydro-3H-pyrazolo[4,3-c]pyridin-3-one* (**A15**). ^1^H NMR (500 MHz, Methanol-*d*_4_) δ 7.58–7.51 (m, 2H), 7.50–7.41 (m, 3H), 7.27 (dd, *J* = 8.5, 5.3 Hz, 2H), 7.04 (t, *J* = 8.7 Hz, 2H), 5.07–4.94 (m, 2H), 4.45–4.25 (m, 2H), 4.03–3.83 (m, 3H), 3.11 (dd, *J* = 17.5, 5.4 Hz, 1H), 2.73 (dd, *J* = 17.4, 5.3 Hz, 1H), 1.51 (d, *J* = 6.6 Hz, 3H). ^13^C NMR (126 MHz, Methanol-*d*_4_) δ 162.38 (d, *J* = 244.8 Hz), 155.19, 141.67, 132.71 (d, *J* = 3.3 Hz), 130.75, 130.22, 129.61, 129.40 (d, *J* = 8.2 Hz), 129.03, 115.03 (d, *J* = 21.8 Hz), 90.01, 55.49, 54.64, 47.48, 43.04, 26.35, 13.78. ^19^F NMR (471 MHz, Methanol-*d*_4_) δ −116.52. HRMS (ESI) calcd for C_21_H_23_FN_3_O^+^ 352.1819 [M + H]^+^, found 352.1814.
*(R)-5-benzyl-2-(4-bromobenzyl)-6-methyl-1,2,4,5,6,7-hexahydro-3H-pyrazolo[4,3-c]pyridin-3-one* (**A16**). ^1^H NMR (500 MHz, Methanol-*d*_4_) δ 7.47–7.36 (m, 5H), 7.30 (d, *J* = 8.5 Hz, 2H), 7.19 (d, *J* = 8.4 Hz, 2H), 5.02–4.93 (m, 2H), 4.14–4.00 (m, 2H), 3.73–3.64 (m, 2H), 3.62–3.54 (m, 1H), 2.91 (dd, *J* = 17.0, 5.4 Hz, 1H), 2.52 (dd, *J* = 17.0, 5.0 Hz, 1H), 1.36 (d, *J* = 6.6 Hz, 3H). ^13^C NMR (126 MHz, Methanol-*d*_4_) δ 158.49, 142.98, 136.18, 133.51, 132.96, 129.97, 128.79, 128.63, 128.52, 128.24, 91.55, 55.29, 54.00, 46.91, 43.34, 27.22, 13.43. HRMS (ESI) calcd for C_21_H_23_BrN_3_O^+^ 412.1019 [M + H]^+^, found 412.1027.
*(R)-5-benzyl-2-(4-iodobenzyl)-6-methyl-1,2,4,5,6,7-hexahydro-3H-pyrazolo[4,3-c]pyridin-3-one* (**A17**). ^1^H NMR (500 MHz, Methanol-*d*_4_) δ 7.64 (d, *J* = 7.9 Hz, 2H), 7.48–7.36 (m, 5H), 6.99 (d, *J* = 8.0 Hz, 2H), 5.02–4.87 (m, 2H), 4.08 (q, *J* = 13.1 Hz, 2H), 3.70 (s, 2H), 3.63–3.55 (m, 1H), 2.92 (dd, *J* = 17.1, 5.4 Hz, 1H), 2.53 (dd, *J* = 17.0, 5.1 Hz, 1H), 1.37 (d, *J* = 6.7 Hz, 3H). ^13^C NMR (126 MHz, Methanol-*d*_4_) δ 158.19, 142.86, 137.39, 137.18, 133.20, 130.04, 129.26, 128.68, 128.64, 127.77, 92.18, 55.23, 54.17, 47.15, 43.35, 27.16, 13.50. HRMS (ESI) calcd for C_21_H_23_IN_3_O^+^ 460.0880 [M + H]^+^, found 460.0885.
*(R)-5-benzyl-6-methyl-2-(4-(trifluoromethyl)benzyl)-1,2,4,5,6,7-hexahydro-3H-pyrazolo[4,3-c]pyridin-3-one* (**A18**). ^1^H NMR (500 MHz, Methanol-*d*_4_) δ 7.59 (d, *J* = 8.0 Hz, 2H), 7.43 (dd, *J* = 13.8, 6.9 Hz, 4H), 7.38 (t, *J* = 8.2 Hz, 3H), 5.14–4.97 (m, 2H), 4.20–3.96 (m, 2H), 3.74–3.63 (m, 2H), 3.55 (h, *J* = 6.4 Hz, 1H), 2.90 (dd, *J* = 16.9, 5.4 Hz, 1H), 2.50 (dd, *J* = 16.9, 5.2 Hz, 1H), 1.35 (d, *J* = 6.7 Hz, 3H). ^13^C NMR (126 MHz, Methanol-*d*_4_) δ 159.24, 143.24, 142.30, 133.80, 129.93, 129.19 (q, *J* = 32.0 Hz), 128.59, 128.43, 127.60, 124.99 (q, *J* = 3.8 Hz), 124.29 (q, *J* = 271.1 Hz), 90.99, 55.32, 53.99, 47.06, 43.60, 27.48, 13.43. ^19^F NMR (471 MHz, Methanol-*d*_4_) δ −63.94. HRMS (ESI) calcd for C_22_H_23_F_3_N_3_O^+^ 402.1787 [M + H]^+^, found 402.1783.
*(R)-5-benzyl-6-methyl-2-(4-(trifluoromethoxy)benzyl)-1,2,4,5,6,7-hexahydro-3H-pyrazolo [4,3-c]pyridin-3-one* (**A19**). ^1^H NMR (500 MHz, Methanol-*d*_4_) δ 7.44–7.37 (m, 5H), 7.31 (d, *J* = 8.3 Hz, 2H), 7.21 (d, *J* = 8.2 Hz, 2H), 5.06–4.95 (m, 2H), 4.11–3.99 (m, 2H), 3.67 (s, 2H), 3.61–3.52 (m, 1H), 2.90 (dd, *J* = 16.9, 5.4 Hz, 1H), 2.50 (dd, *J* = 17.0, 5.0 Hz, 1H), 1.36 (d, *J* = 6.6 Hz, 3H). ^13^C NMR (126 MHz, Methanol-*d*_4_) δ 154.81, 148.59, 141.76, 135.90, 130.84, 130.03, 129.53, 129.22, 128.99, 120.87, 120.48 (q, *J* = 255.7 Hz), 89.82, 55.11, 54.43, 53.15, 42.82, 26.01, 13.65. ^19^F NMR (471 MHz, Methanol-*d*_4_) δ −59.57. HRMS (ESI) calcd for C_22_H_23_F_3_N_3_O_2_^+^ 418.1736 [M + H]^+^, found 418.1733.
*(R)-5-benzyl-2-(4-(difluoromethoxy)benzyl)-6-methyl-1,2,4,5,6,7-hexahydro-3H-pyrazolo[4,3-c]pyridin-3-one* (**A20**). ^1^H NMR (500 MHz, Methanol-*d*_4_) δ 7.43 (dd, *J* = 19.6, 7.1 Hz, 5H), 7.26 (d, *J* = 8.2 Hz, 2H), 7.08 (d, *J* = 8.2 Hz, 2H), 6.78 (td, *J* = 74.1, 1.6 Hz, 1H), 5.04–4.91 (m, 2H), 4.17–4.03 (m, 2H), 3.72 (d, *J* = 3.2 Hz, 2H), 3.65–3.57 (m, 1H), 2.94 (dd, *J* = 17.2, 5.4 Hz, 1H), 2.55 (dd, *J* = 17.2, 5.1 Hz, 1H), 1.38 (d, *J* = 6.6 Hz, 3H). ^13^C NMR (126 MHz, Methanol-*d*_4_) δ 150.90, 142.78, 134.27, 134.25, 130.08, 130.05, 128.84, 128.69, 128.68, 118.94, 116.32 (t, *J* = 257.9 Hz), 91.53, 55.26, 54.20, 46.99, 43.27, 27.08, 13.46. ^19^F NMR (471 MHz, Methanol-*d*_4_) δ −83.38. HRMS (ESI) calcd for C_22_H_24_F_2_N_3_O_2_^+^ 400.1831 [M + H]^+^, found 400.1826.
*(R)-5-benzyl-2-(2,3-difluorobenzyl)-6-methyl-1,2,4,5,6,7-hexahydro-3H-pyrazolo[4,3-c]pyridin-3-one* (**A21**). ^1^H NMR (500 MHz, Methanol-*d*_4_) δ 7.49–7.40 (m, 5H), 7.17 (q, *J* = 8.6 Hz, 1H), 7.07 (q, *J* = 7.5 Hz, 1H), 6.87 (t, *J* = 7.1 Hz, 1H), 5.16–5.05 (m, 2H), 4.90 (s, 1H), 4.15 (q, *J* = 13.0 Hz, 2H), 3.83–3.72 (m, 2H), 3.71–3.62 (m, 1H), 2.97 (dd, *J* = 17.2, 5.3 Hz, 1H), 2.58 (dd, *J* = 17.1, 5.0 Hz, 1H), 1.41 (d, *J* = 6.5 Hz, 3H). ^13^C NMR (126 MHz, Methanol-*d*_4_) δ 149.38, 147.29, 147.19, 143.09, 132.60, 130.16, 128.84, 128.74, 127.03 (d, *J* = 11.6 Hz), 124.14, 124.09, 116.04 (d, *J* = 17.2 Hz), 90.62, 55.03, 54.58, 43.46, 41.11, 27.16, 13.51. ^19^F NMR (471 MHz, Methanol-*d*_4_) δ −141.40 (dd, *J* = 20.6, 8.1 Hz), −146.42 (dd, *J* = 20.0, 7.6 Hz). HRMS (ESI) calcd for C_21_H_22_F_2_N_3_O^+^ 370.1725 [M + H]^+^, found 370.1721.
*(R)-5-benzyl-2-(2,4-difluorobenzyl)-6-methyl-1,2,4,5,6,7-hexahydro-3H-pyrazolo[4,3-c]pyridin-3-one* (**A22**). ^1^H NMR (500 MHz, Methanol-*d*_4_) δ 7.54–7.36 (m, 5H), 7.16–7.10 (m, 1H), 6.95 (td, *J* = 9.7, 2.6 Hz, 1H), 6.89 (td, *J* = 8.6, 2.4 Hz, 1H), 5.11–4.97 (m, 2H), 4.19–4.07 (m, 2H), 3.80–3.70 (m, 2H), 3.69–3.62 (m, 1H), 2.95 (d, *J* = 17.7 Hz, 1H), 2.56 (d, *J* = 17.2 Hz, 1H), 1.40 (d, *J* = 6.9 Hz, 3H). ^13^C NMR (126 MHz, Methanol-*d*_4_) δ 163.73–159.22 (m), 163.61–159.10 (m), 161.47, 158.44, 143.24, 133.22, 130.48 (dd, *J* = 9.8, 5.6 Hz), 130.06, 128.67, 120.62 (dd, *J* = 15.1, 3.8 Hz), 110.95 (dd, *J* = 21.5, 3.7 Hz), 103.15 (t, *J* = 25.8 Hz), 90.85, 55.14, 54.19, 43.41, 40.93 (d, *J* = 4.3 Hz), 27.28, 13.52. ^19^F NMR (471 MHz, Methanol-*d*_4_) δ −113.06, −116.33. HRMS (ESI) calcd for C_21_H_22_F_2_N_3_O^+^ 370.1725 [M + H]^+^, found 370.1724.
*(R)-5-benzyl-2-(2,5-difluorobenzyl)-6-methyl-1,2,4,5,6,7-hexahydro-3H-pyrazolo[4,3-c]pyridin-3-one* (**A23**). ^1^H NMR (500 MHz, Methanol-*d*_4_) δ 7.50–7.36 (m, 5H), 7.11 (td, *J* = 9.2, 4.3 Hz, 1H), 7.01 (tt, *J* = 8.1, 3.5 Hz, 1H), 6.77–6.64 (m, 1H), 5.13–4.99 (m, 2H), 4.23–4.07 (m, 2H), 3.86–3.74 (m, 2H), 3.74–3.61 (m, 1H), 2.98 (dd, *J* = 17.1, 5.4 Hz, 1H), 2.59 (dd, *J* = 17.1, 5.1 Hz, 1H), 1.48–1.38 (m, 3H). ^13^C NMR (126 MHz, Methanol-*d*_4_) δ 161.33–158.38 (m), 161.25–158.38 (m), 159.56, 156.66 (d, *J* = 2.6 Hz), 144.34, 133.69, 131.60, 130.30, 130.16, 128.05 (dd, *J* = 17.4, 7.7 Hz), 117.54 (dd, *J* = 24.5, 8.6 Hz), 116.68–116.32 (m), 90.94, 56.30, 56.11, 45.01, 42.70 (d, *J* = 4.7 Hz), 28.60, 14.92. ^19^F NMR (471 MHz, Methanol-*d*_4_) δ −120.81, −126.80. HRMS (ESI) calcd for C_21_H_22_F_2_N_3_O^+^ 370.1725 [M + H]^+^, found 370.1722.
*(R)-5-benzyl-2-(3,4-difluorobenzyl)-6-methyl-1,2,4,5,6,7-hexahydro-3H-pyrazolo[4,3-c]pyridin-3-one* (**A24**). ^1^H NMR (500 MHz, Methanol-*d*_4_) δ 7.50–7.34 (m, 5H), 7.18 (dt, *J* = 10.6, 8.3 Hz, 1H), 7.09 (ddd, *J* = 10.7, 7.7, 2.1 Hz, 1H), 7.05–6.99 (m, 1H), 4.98–4.96 (m, 2H), 4.10 (q, *J* = 13.0 Hz, 2H), 3.78–3.66 (m, 2H), 3.64–3.56 (m, 1H), 2.94 (dd, *J* = 17.0, 5.4 Hz, 1H), 2.54 (dd, *J* = 17.0, 5.1 Hz, 1H), 1.38 (d, *J* = 6.6 Hz, 3H). ^13^C NMR (126 MHz, Methanol-*d*_4_) δ 158.27, 150.09 (dd, *J* = 246.8, 12.8 Hz), 149.52 (dd, *J* = 246.3, 12.7 Hz), 142.92, 135.14 (t, *J* = 4.6 Hz), 133.04, 130.07, 128.69, 123.66, (d, *J* = 3.7 Hz), 123.61 (d, *J* = 3.8 Hz), 116.92 (d, *J* = 17.5 Hz), 116.12 (d, *J* = 17.9 Hz), 90.58, 55.14, 54.33, 46.67, 43.51, 27.27, 13.49. ^19^F NMR (471 MHz, Methanol-*d*_4_) δ −140.34 (d, *J* = 20.0 Hz), −142.53 (d, *J* = 20.8 Hz). HRMS (ESI) calcd for C_21_H_22_F_2_N_3_O^+^ 370.1725 [M + H]^+^, found 370.1720.
*(R)-5-benzyl-2-(3,5-difluorobenzyl)-6-methyl-1,2,4,5,6,7-hexahydro-3H-pyrazolo[4,3-c]pyridin-3-one* (**A25**). ^1^H NMR (500 MHz, Methanol-*d*_4_) δ 7.52–7.36 (m, 5H), 6.86–6.72 (m, 3H), 5.03 (d, *J* = 16.2 Hz, 2H), 4.22–4.07 (m, 2H), 3.76 (d, *J* = 3.6 Hz, 2H), 3.70–3.60 (m, 1H), 2.96 (dd, *J* = 17.0, 5.4 Hz, 1H), 2.57 (dd, *J* = 17.0, 5.1 Hz, 1H), 1.41 (d, *J* = 6.7 Hz, 3H). ^13^C NMR (126 MHz, Methanol-*d*_4_) δ 164.59 (d, *J* = 247.4 Hz), 164.49 (d, *J* = 247.4 Hz), 160.23, 144.39, 143.93, 143.93 (d, *J* = 17.5 Hz), 134.44, 131.46, 130.07, 111.23 (d, *J* = 6.1 Hz), 111.08 (d, *J* = 6.0 Hz), 103.37 (t, *J* = 25.8 Hz), 91.30, 56.48, 55.77, 48.23, 45.13, 28.80, 14.88. ^19^F NMR (471 MHz, Methanol-*d*_4_) δ −111.86. HRMS (ESI) calcd for C_21_H_22_F_2_N_3_O^+^ 370.1725 [M + H]^+^, found 370.1723.
*(R)-5-benzyl-6-methyl-2-(3,4,5-trifluorobenzyl)-1,2,4,5,6,7-hexahydro-3H-pyrazolo[4,3-c]pyridin-3-one* (**A26**). ^1^H NMR (500 MHz, Methanol-*d*_4_) δ 7.48–7.39 (m, 5H), 6.94 (dd, *J* = 8.5, 6.6 Hz, 2H), 4.95 (d, *J* = 9.3 Hz, 2H), 4.21–4.10 (m, 2H), 3.77 (d, *J* = 3.5 Hz, 2H), 3.71–3.62 (m, 1H), 2.96 (dd, *J* = 17.0, 5.4 Hz, 1H), 2.57 (dd, *J* = 17.0, 5.2 Hz, 1H), 1.42 (d, *J* = 6.6 Hz, 3H). ^13^C NMR (126 MHz, Methanol-*d*_4_) δ 158.6, 150.94 (ddd, *J* = 248.6, 9.9, 3.7 Hz), 143.0, 138.59 (dt, *J* = 249.0, 15.7 Hz), 135.3–135.1 (m), 132.6, 130.2, 128.8, 128.7, 111.52–111.00 (m), 89.3, 55.0, 54.7, 46.5, 43.8, 27.4, 13.5. ^19^F NMR (471 MHz, Methanol-*d*_4_) δ −137.07, −166.01. HRMS (ESI) calcd for C_21_H_21_F_3_N_3_O^+^ 388.1631 [M + H]^+^, found 388.1629.
General Procedure for the Preparation of **A27**–**A37**. Intermediate **7d** was prepared by following the procedure described for our previous work [28].
*tert-butyl-(R)-6-methyl-3-oxo-1,2,3,4,6,7-hexahydro-5H-pyrazolo[4,3-c]pyridine-5-carboxylate* (**7e**). Compound **7d** (2.50 g, 8.00 mmol) was dissolved in EtOH, and hydrazine hydrate (4 mL) was added. The mixture was stirred at reflux at 80 °C for 2 h. Upon completion, the solvent was removed in vacuo to afford the compound **7e** (1.82 g, 90% yield) as a yellow solid.
*di-tert-butyl-(R)-6-methyl-3-oxo-2,3,6,7-tetrahydro-1H-pyrazolo[4,3-c]pyridine-1,5(4H)-dicarboxylate* (**7f**). Compound **7e** (1.82 g, 7.20 mmol) was dissolved in DCM, and Boc_2_O (2.10 g, 8.60 mmol) was added dropwise under ice-bath conditions. The resulting mixture was stirred at room temperature overnight. After the reaction was completed, the solvent was removed under reduced pressure to afford the crude compound **7f** (2.23 g, 88% yield).
*di-tert-butyl-(R)-2-(4-bromobenzyl)-6-methyl-3-oxo-2,3,6,7-tetrahydro-1H-pyrazolo[4,3-c]pyridine-1,5(4H)-dicarboxylate* (**7g**). Compound **7f** (2.23 g, 6.30 mmol) was dissolved in DMF (5 mL), and NaH (240 mg, 6.30 mmol) was added. The mixture was stirred at room temperature for 1 h, followed by the addition of 4-bromobenzyl bromide (1.74 g, 6.93 mmol). The reaction was stirred at room temperature for an additional 12 h, and then quenched with H_2_O (30 mL) and extracted with EA (3 × 30 mL). The combined organic layers were dried over Na_2_SO_4_, filtered and concentrated under reduced pressure. The residue was purified via silica gel chromatography (PE:EA = 2:1) to afford the title compound **7g** (3.90 g, 83% yield) as a yellow solid.
*(R)-2-(4-bromobenzyl)-6-methyl-1,2,4,5,6,7-hexahydro-3H-pyrazolo[4,3-c]pyridin-3-one* (**7h**). To a solution of the compound **7g** (3.90 g, 7.46 mmol) in DCM (15 mL) was added TFA (3 mL) and the mixture was stirred at room temperature. After the reaction was completed, the solvent was removed in vacuo, quenched by saturated NaHCO_3_ aqueous solution, and extracted with DCM. The combined organic layers were washed with brine, dried over Na_2_SO_4_, filtered and concentrated under reduced pressure to afford the crude **7h** (1.93 g, 80% yield) without purification. Then, a mixture of the obtained intermediate **7h** (160 mg, 0.50 mmol), various substituted benzaldehydes, and DIPEA (88 μL, 0.5 mmol) was dissolved in anhydrous DCM (5 mL). Activated molecular sieves were then added, and the reaction was stirred at room temperature for 1 h under an nitrogen atmosphere. Subsequently, sodium triacetoxyborohydride (211 mg, 1.00 mmol) was added, and the mixture was stirred at room temperature for 12 h. Upon completion, the molecular sieves were removed by filtration through a pad of Celite. The filtrate was diluted with H_2_O (20 mL) and extracted with DCM (3 × 20 mL). The combined organic layers were concentrated in vacuo, and the crude residues were purified by reversed-phase chromatography to afford the target compounds **A27**–**A37** in 10–20% yields.
*(R)-2-(4-bromobenzyl)-5-(2-fluorobenzyl)-6-methyl-1,2,4,5,6,7-hexahydro-3H-pyrazolo[4,3-c]pyridin-3-one* (**A27**). ^1^H NMR (500 MHz, Methanol-*d*_4_) δ 7.54–7.43 (m, 3H), 7.43–7.36 (m, 1H), 7.22 (td, *J* = 7.5, 1.2 Hz, 1H), 7.19–7.10 (m, 3H), 5.02–4.91 (m, 2H), 4.08–3.97 (m, 2H), 3.64 (s, 2H), 3.51 (h, *J* = 6.6 Hz, 1H), 2.87 (dd, *J* = 17.0, 5.4 Hz, 1H), 2.49 (dd, *J* = 17.0, 5.4 Hz, 1H), 1.33 (d, *J* = 6.6 Hz, 3H). ^13^C NMR (126 MHz, Methanol-*d*_4_) δ 161.64 (d, *J* = 246.5 Hz), 143.93, 136.16, 132.10, 131.34, 130.31 (d, *J* = 8.3 Hz), 132.07, 129.10, 124.33, 124.30, 121.09, 115.20 (d, *J* = 21.7 Hz), 101.42, 63.03, 53.71, 46.84, 43.07, 27.16, 13.59. ^19^F NMR (471 MHz, Methanol-*d*_4_) δ −118.74. HRMS (ESI) calcd for C_21_H_22_BrFN_3_O^+^ 430.0924 [M + H]^+^, found 430.0919.
*(R)-2-(4-bromobenzyl)-5-(3-fluorobenzyl)-6-methyl-1,2,4,5,6,7-hexahydro-3H-pyrazolo[4,3-c]pyridin-3-one* (**A28**). ^1^H NMR (500 MHz, Methanol-*d*_4_) δ 7.47 (d, *J* = 8.1 Hz, 2H), 7.38 (td, *J* = 8.0, 6.0 Hz, 1H), 7.23–7.17 (m, 2H), 7.15 (d, *J* = 8.1 Hz, 2H), 7.06 (td, *J* = 8.5, 2.6 Hz, 1H), 5.01–4.92 (m, 2H), 3.98–3.84 (m, 2H), 3.51 (s, 2H), 3.40 (h, *J* = 6.1 Hz, 1H), 2.84 (dd, *J* = 16.8, 5.4 Hz, 1H), 2.42 (dd, *J* = 16.8, 4.7 Hz, 1H), 1.26 (d, *J* = 6.6 Hz, 3H). ^13^C NMR (126 MHz, Methanol-*d*_4_) δ 163.03 (d, *J* = 245.0 Hz), 159.40, 144.33, 136.14, 131.36, 131.15, 130.06 (d, *J* = 8.1 Hz), 129.13, 125.16, 121.10, 115.91 (d, *J* = 22.1 Hz), 114.43 (d, *J* = 21.0 Hz), 87.11, 54.42, 52.76, 46.69, 42.37, 27.45, 13.24. ^19^F NMR (471 MHz, Methanol-*d*_4_) δ −115.04. HRMS (ESI) calcd for C_21_H_22_BrFN_3_O^+^ 430.0924 [M + H]^+^, found 430.0927.
*(R)-2-(4-bromobenzyl)-5-(4-fluorobenzyl)-6-methyl-1,2,4,5,6,7-hexahydro-3H-pyrazolo [4,3-c]pyridin-3-one* (**A29**). ^1^H NMR (500 MHz, Methanol-*d*_4_) δ 7.63–7.35 (m, 5H), 7.22–7.08 (m, 3H), 7.13–7.02 (m, 1H), 5.19–4.97 (m, 2H), 4.12–3.81 (m, 2H), 3.68–3.49 (m, 2H), 3.51–3.41 (m, 1H), 2.87 (dd, *J* = 16.9, 5.4 Hz, 1H), 2.46 (dd, *J* = 16.9, 4.8 Hz, 1H), 1.31 (d, *J* = 6.6 Hz, 3H). ^19^F NMR (471 MHz, Methanol-*d*_4_) δ −75.79. HRMS (ESI) calcd for C_21_H_22_BrFN_3_O^+^ 430.0924 [M + H]^+^, found 430.0927.
*(R)-2-(4-bromobenzyl)-5-(3-chlorobenzyl)-6-methyl-1,2,4,5,6,7-hexahydro-3H-pyrazolo[4,3-c]pyridin-3-one* (**A30**). ^1^H NMR (500 MHz, Methanol-*d*_4_) δ 7.55–7.45 (m, 3H), 7.38–7.31 (m, *J* = 3.1 Hz, 3H), 7.15 (d, *J* = 8.2 Hz, 2H), 5.04–4.91 (m, 2H), 4.00–3.81 (m, 2H), 3.56–3.47 (m, 2H), 3.41 (h, *J* = 6.2 Hz, 1H), 2.85 (dd, *J* = 16.9, 5.5 Hz, 1H), 2.43 (dd, *J* = 16.9, 4.8 Hz, 1H), 1.27 (d, *J* = 6.6 Hz, 3H). ^13^C NMR (126 MHz, Methanol-*d*_4_) δ 165.16, 144.34, 138.56, 136.03, 134.20, 131.38, 129.84, 129.27, 129.13, 127.80, 127.74, 121.15, 96.78, 55.42, 52.88, 46.72, 42.75, 27.43, 13.29. HRMS (ESI) calcd for C_21_H_22_BrClN_3_O^+^ 446.0629 [M + H]^+^, found 446.0627.
*(R)-5-(3-bromobenzyl)-2-(4-bromobenzyl)-6-methyl-1,2,4,5,6,7-hexahydro-3H-pyrazolo[4,3-c]pyridin-3-one* (**A31**). ^1^H NMR (500 MHz, Methanol-*d*_4_) δ 7.64 (s, 1H), 7.52 (d, *J* = 6.6 Hz, 1H), 7.47 (d, *J* = 8.0 Hz, 2H), 7.40 (d, *J* = 7.6 Hz, 1H), 7.31 (t, *J* = 7.9 Hz, 1H), 7.15 (d, *J* = 8.1 Hz, 2H), 5.01–4.94 (m, 2H), 4.06–3.90 (m, 2H), 3.65–3.55 (m, 2H), 3.55–3.47 (m, 1H), 2.90 (dd, *J* = 17.0, 5.4 Hz, 1H), 2.49 (dd, *J* = 17.0, 4.8 Hz, 1H), 1.31 (d, *J* = 6.6 Hz, 3H). ^13^C NMR (126 MHz, Methanol-*d*_4_) δ 158.20, 135.98, 132.53, 131.40, 131.24, 129.79, 129.37, 129.17, 128.49, 125.12, 122.38, 121.19, 94.04, 55.04, 53.54, 46.94, 42.80, 27.17, 13.41. HRMS (ESI) calcd for C_21_H_22_Br_2_N_3_O^+^ 492.0103 [M + H]^+^, found 492.0107.
*(R)-2-(4-bromobenzyl)-6-methyl-5-(3-methylbenzyl)-1,2,4,5,6,7-hexahydro-3H-pyrazolo[4,3-c]pyridin-3-one* (**A32**). ^1^H NMR (500 MHz, Methanol-*d*_4_) δ 7.47 (d, *J* = 8.0 Hz, 2H), 7.34 (ddd, *J* = 23.3, 14.3, 7.3 Hz, 4H), 7.17 (d, *J* = 8.0 Hz, 2H), 5.07–4.97 (m, 2H), 4.41–4.21 (m, 2H), 4.04–3.84 (m, 3H), 3.12 (dd, *J* = 17.5, 5.5 Hz, 1H), 2.76 (dd, *J* = 17.5, 5.5 Hz, 1H), 2.39 (s, 3H), 1.53 (d, *J* = 6.6 Hz, 3H). ^13^C NMR (126 MHz, Methanol-*d*_4_) δ 155.01, 141.69, 139.23, 135.83, 131.42, 131.21, 130.42, 129.75, 129.25, 129.00, 127.66, 121.29, 89.89, 55.79, 54.67, 47.11, 43.01, 26.26, 19.96, 13.74. HRMS (ESI) calcd for C_22_H_25_BrN_3_O^+^ 426.1175 [M + H]^+^, found 426.1169.
*(R)-2-(4-bromobenzyl)-6-methyl-5-(3-(trifluoromethyl)benzyl)-1,2,4,5,6,7-hexahydro-3H-pyrazolo[4,3-c]pyridin-3-one* (**A33**). ^1^H NMR (500 MHz, Methanol-*d*_4_) δ 7.72 (s, 1H), 7.66 (d, *J* = 7.6 Hz, 1H), 7.60 (d, *J* = 7.9 Hz, 1H), 7.55 (t, *J* = 7.7 Hz, 1H), 7.47 (d, *J* = 8.1 Hz, 2H), 7.15 (d, *J* = 8.1 Hz, 2H), 4.99–4.91 (m, 2H), 3.97–3.82 (m, 2H), 3.42 (s, 2H), 3.29–3.14 (m, 1H), 2.81 (dd, *J* = 16.8, 5.4 Hz, 1H), 2.38 (dd, *J* = 16.8, 4.7 Hz, 1H), 1.22 (d, *J* = 6.6 Hz, 3H). ^13^C NMR (126 MHz, Methanol-*d*_4_) δ 159.69, 144.97, 139.00, 135.93, 132.82, 131.40, 130.47 (q, *J* = 32.0 Hz), 129.13, 128.97, 125.56, 124.29 (q, *J* = 271.5 Hz), 124.05, 121.18, 96.81, 55.80, 52.30, 46.56, 42.57, 27.60, 13.25. ^19^F NMR (471 MHz, Methanol-*d*_4_) δ −64.01. HRMS (ESI) calcd for C_22_H_22_BrF_3_N_3_O^+^ 480.0892 [M + H]^+^, found 480.0887.
*(R)-2-(4-bromobenzyl)-6-methyl-5-(3-(trifluoromethoxy)benzyl)-1,2,4,5,6,7-hexahydro-3H-pyrazolo[4,3-c]pyridin-3-one* (**A34**). ^1^H NMR (500 MHz, Methanol-*d*_4_) δ 7.53–7.45 (m, 2H), 7.43 (d, *J* = 7.8 Hz, 1H), 7.39 (s, 1H), 7.27 (d, *J* = 8.1 Hz, 1H), 7.17 (dd, *J* = 8.4, 2.2 Hz, 2H), 7.09–7.00 (m, 1H), 5.03–4.91 (m, 2H), 3.91–3.80 (m, 2H), 3.58–3.53 (m, 2H), 3.50–3.40 (m, 1H), 2.85 (dd, *J* = 37.4, 5.4 Hz, 1H), 2.39 (dd, *J* = 16.8, 4.6 Hz, 1H), 1.22 (d, *J* = 6.6 Hz, 3H). ^13^C NMR (126 MHz, Methanol-*d*_4_) δ 164.14, 162.17, 149.42, 135.78, 131.44, 130.01, 129.15, 128.06, 125.7 (q, *J* = 267.12 Hz), 121.68, 121.26, 120.15, 111.55 (d, *J* = 25.1 Hz), 102.35, 55.47, 52.29, 46.62, 42.57, 27.48, 13.24. ^19^F NMR (471 MHz, Methanol-*d*_4_) δ −59.31. HRMS (ESI) calcd for C_22_H_22_BrF_3_N_3_O_2_^+^ 496.0842 [M + H]^+^, found 496.0835.
*(R)-2-(4-bromobenzyl)-5-(3,4-difluorobenzyl)-6-methyl-1,2,4,5,6,7-hexahydro-3H-pyrazolo[4,3-c]pyridin-3-one* (**A35**). ^1^H NMR (500 MHz, Methanol-*d*_4_) δ 7.47 (dd, *J* = 8.6, 2.2 Hz, 2H), 7.38 (ddd, *J* = 11.3, 7.7, 2.1 Hz, 1H), 7.33–7.20 (m, 2H), 7.15 (d, *J* = 8.4 Hz, 2H), 5.03–4.91 (m, 2H), 4.06–3.91 (m, 2H), 3.64–3.55 (m, 2H), 3.51 (h, *J* = 6.1 Hz, 1H), 2.90 (dd, *J* = 17.0, 5.4 Hz, 1H), 2.49 (dd, *J* = 17.0, 4.7 Hz, 1H), 1.30 (d, *J* = 6.6 Hz, 3H). ^13^C NMR (126 MHz, Methanol-*d*_4_) δ 157.92, 150.23 (dd, *J* = 247.3, 12.8 Hz), 150.17 (dd, *J* = 248.2, 12.6 Hz), 143.79, 135.86, 132.47, 131.41, 129.16, 126.19, 121.24, 118.43 (d, *J* = 17.4 Hz), 117.21 (d, *J* = 17.4 Hz), 94.29, 54.70, 53.36, 46.93, 42.61, 27.14, 13.25. ^19^F NMR (471 MHz, Methanol-*d*_4_) δ −139.75, −140.80. HRMS (ESI) calcd for C_21_H_21_BrF_2_N_3_O^+^ 448.0831 [M + H]^+^, found 448.0823.
*(R)-2-(4-bromobenzyl)-5-(3,5-difluorobenzyl)-6-methyl-1,2,4,5,6,7-hexahydro-3H-pyrazolo[4,3-c]pyridin-3-one* (**CLPP-3036**). ^1^H NMR (500 MHz, Methanol-*d*_4_) δ 7.50 (d, *J* = 8.3 Hz, 2H), 7.18 (d, *J* = 8.4 Hz, 2H), 7.09 (d, *J* = 6.2 Hz, 2H), 6.97–6.90 (m, 1H), 5.11–4.82 (m, 3H), 3.98 (qd, *J* = 13.6, 2.6 Hz, 2H), 3.63–3.53 (m, 2H), 3.50–3.40 (m, 1H), 2.91 (dd, *J* = 17.0, 5.3 Hz, 1H), 2.48 (dd, *J* = 17.0, 4.7 Hz, 1H), 1.29 (d, *J* = 6.5 Hz, 3H). ^13^C NMR (126 MHz, Methanol-*d*_4_) δ 163.24 (dd, *J* = 248.0, 12.8 Hz), 158.32, 144.17, 140.43, 135.73, 131.44, 129.18, 121.30, 112.02 (d, *J* = 24.8 Hz), 102.93 (t, *J* = 25.6 Hz), 95.42, 55.11, 53.11, 46.85, 42.67, 27.21, 13.31. ^19^F NMR (471 MHz, MeOD) δ −111.31. HRMS (ESI) calcd for C_21_H_21_BrF_2_N_3_O^+^ 448.0831 [M + H]^+^, found 448.0825.
*(R)-4-((2-(4-bromobenzyl)-6-methyl-3-oxo-1,2,3,4,6,7-hexahydro-5H-pyrazolo[4,3-c]pyridin-5-yl)methyl)-2-fluorobenzonitrile* (**A37**). ^1^H NMR (500 MHz, Methanol-*d*_4_) δ 7.71 (dd, *J* = 7.9, 6.7 Hz, 1H), 7.49 (d, *J* = 8.3 Hz, 2H), 7.44–7.37 (m, 2H), 7.16 (d, *J* = 8.1 Hz, 2H), 4.99–4.89 (m, 2H), 3.92–3.79 (m, 2H), 3.38 (s, 2H), 3.30–3.21 (m, 1H), 2.79 (dd, *J* = 16.7, 5.4 Hz, 1H), 2.35 (dd, *J* = 16.7, 4.5 Hz, 1H), 1.17 (d, *J* = 6.6 Hz, 3H). ^13^C NMR (126 MHz, Methanol-*d*_4_) δ 163.22 (d, *J* = 256.5 Hz), 148.51, 145.32, 135.63, 133.34, 131.46, 129.17, 125.23 (d, *J* = 3.1 Hz), 121.32, 116.09 (d, *J* = 20.3 Hz), 113.44, 99.37 (d, *J* = 15.2 Hz), 97.97, 55.66, 51.96, 46.52, 42.50, 27.48, 13.31. ^19^F NMR (471 MHz, Methanol-*d*_4_) δ −110.08. HRMS (ESI) calcd for C_22_H_21_BrFN_4_O^+^ 455.0877 [M + H]^+^, found 455.0870.

### 3.2. Protein Preparation for In Vitro Enzyme Assay

The preparation of ClpP protein was carried out as reported in the previous literature. Generally, ClpP sequence 57−277 with an N-terminal SUMO tag was cloned and transformed in Escherichia coli. The bacteria were cultured with LB medium in the presence of 50 μg/mL ampicillin at 37 °C. The bacteria were harvested after treatment with 0.5 mM isopropyl β D-1 thiogalactopyranoside for 4 h and sequentially lysed in buffer (25 mM Tris-2HCl pH 7.5, 0.5 M NaCl, 5 mM β-mercaptoethanol, 10 mM imidazole, 10% glycerol, 1% Triton X-100) by sonication. The pellet was removed by centrifugation, the supernatant was combined with Ni sepharose 6 Fast Flow for 2 h, and then purified using a gravity column, first with washing buffer (25 mM Tris-2HCl pH 7.5, 0.5 M NaCl, 5 mM β-mercaptoethanol, 10 mM imidazole, 10% glycerol) for the first impurity protein elution and finally with 400 mM imidazole for the protein of interest. The eluted protein was dialyzed against 4 L of dialysate solution for 6 h. Subsequently, the His-Sumo tag fused to ClpP was cleaved via overnight incubation with Ulp1 protease at 16 °C. Protein concentration was determined using the BCA method, and the purified protein was finally preserved at −80 °C in storage buffer supplemented with 50% glycerol.

### 3.3. In Vitro Enzyme Assay

The ClpP protein was diluted with buffer (50 mM Tris-2HCl pH 8, 10 mM MgCl_2_, 100 mM KCl, 1 mM DTT, 5% glycerol, and 0.02% Triton X-100) solution to the final concentration of 0.7 μM and AC-WLA-AMC to the final concentration of 100 μM. In 384 black plate, 10 μL of ClpP protein was first added, and then 5 μL of the compound (The 10 mM compound was diluted 5 times with DMSO to generate seven concentrations, and subsequently diluted 100 times using ddH_2_O) was added, and set wells with 0.02% DMSO (10 μL protein, 20 μL AC-WLA-AMC, 20 μL 0.02%DMSO) and blank wells (10 μL buffer, 20 AC-WLA-AMC, 20 μL 0.02%DMSO). After mixing, it was incubated at room temperature for 10 min. Finally, 10 μL of AC-WLA-AMC was added. The first fluorescence signal was detected at 355 nm and 460 nm wavelengths using a multifunctional enzyme labeler. After detection, the board was sealed and incubated in 37 incubators for 6 h, and the fluorescence signal was collected for a second time. The obtained data were fitted with the enzyme activity curve and EC_50_ values were fitted with a variable-slope four-parameter model in GraphPad Prism 3 based on the fold increase formula:

Fold increase = [(OD_2_(compound) − OD_2_(blank)) − (OD_1_(compound) − OD_1_(blank))/h]

[(OD_2_(DMSO) − OD_2_(blank)) − (OD_1_(DMSO) − OD_1_(blank))/h]

### 3.4. Cell Lines and Cell Culture

The MV4-11 cells were obtained from the American Type Culture Collection (Manassas, VA, USA). MOLM13 was obtained from the Japanese Collection of Research Bioresources Cell Bank (JCRB, Osaka, Japan). Raji, Mia-paca2, A375 and H1299 were obtained from the Cell Bank of Chinese Academy of Sciences, Shanghai Institutes for Biological Sciences. All cells were maintained in the appropriate culture medium suggested by the suppliers. All cell lines were authenticated by STR profiling, tested for mycoplasma contamination, and used within 15 passages and less than 6 months.

### 3.5. Growth Inhibition Assay

Briefly, 80 μL of cells (H1299 and Mia-PaCa2 1 × 10^3^ per well, A375 2 × 10^3^ per well Raji and MOLM13 1 × 10^4^ per well) were seeded into 96-well plates. After 0.5 h or 12 h of incubation (Suspend cells were incubated for 0.5 hours to stabilize; adherent cells were seeded and incubated overnight (12 h) for full attachment prior to drug administration), the cells were treated with 20 μL of 0.02% DMSO or varying concentrations (The 10 mM ONC201 and 100 μM CLPP-3036 was diluted 3 times with DMSO to generate seven concentrations, and subsequently diluted 100 times using complete medium) of the test compounds for 72 h. Cell viability was measured using the Cell Counting Kit-8 (CCK-8, Rare Bio, MA0225S; Shanghai Shenger Biotechnology Co., Ltd, Shanghai, China). The combined solution of CCK-8 (10 μL) was pipetted into each well of the 96-well plates and incubated at 37 °C for 2–4 h. Optical density was determined at 450 nm (background values measured at 650 nm were subtracted) using a Spectra Max 340 microplate reader (Molecular Devices, Sunnyvale, CA, USA). IC_50_ values were fitted using the log(inhibitor) vs. normalized response variable-slope model in GraphPad Prism. Cell growth inhibition rate was calculated according to the formula: inhibition (%) = [1 − (OD compound − OD blank)/(OD DMSO − OD blank)] × 100.

### 3.6. Western Blot

4 × 10^5^ cells were seeded into 12-well plates and treated with compounds at various concentrations for 36 h. Cells were harvested by centrifugation at 5000 rpm for 3 min, resuspended in 100 μL of 1× loading buffer, and denatured at 100 °C for 10 min to obtain whole-cell protein extracts. Protein samples were separated by 15% tricine SDS-PAGE and subsequently transferred onto nitrocellulose membranes. The membranes were blocked with TBS-T containing 5% nonfat dry milk for 1 h at room temperature, followed by incubation with specific primary antibodies overnight at 4 °C. After three washes with TBS-T, the membranes were incubated with horseradish peroxidase (HRP)-conjugated secondary antibodies for 1 h at room temperature. Following another three washes with TBS-T, the membranes were incubated with ECL chemiluminescence reagent, and protein signals were detected using a ChemiDoc Touch Imaging System (E-blot, Yibote Life Sciences Co., Ltd., Shanghai, China). The primary antibodies used were as follows: NDUFA12 (ABclonal, A8237, ABclonal Technology Co., Ltd., Wuhan, China), SDHB (HUABIO, ET1706-30, Hangzhou HuaAn Biotechnology Co., Ltd., Hangzhou, China), and α-Tubulin (Proteintech, 66031-1, Proteintech Group, Inc., Chicago, IL, USA).

### 3.7. Apoptosis

10 × 10^5^ cells were added to the 6-well plate and treated with compounds of different concentrations for 36 h. After drug treatment, the cells were washed twice with ice-cold 1× PBS. 300 μL of 1× Binding buffer (Vazyme, A211-02, Nanjing Vazyme Biotech Co., Ltd., Nanjing, China) was added to each sample. After 2.5 μL of PI and Annexin V-FITC solution was added to each sample, it was immediately analyzed using flow cytometry (Agilent, Agilent Technologies Inc., Santa Clara, CA, USA). Flow cytometry data were analyzed using Agilent software 1.6.2. The gating strategy was as follows: first, intact cells were gated on FSC-A vs. SSC-A (P1); single cells were then selected on FSC-H vs. FSC-A (P2) to exclude doublets; finally, apoptotic cells were quantified in the Annexin V-FITC vs. PI plot, with early apoptotic cells (Q4-4) and late apoptotic cells (Q4-2) summed as the total apoptosis rate. Perform quick-compensation adjustment for PI- and FITC-single-stained wells using Agilent software.

### 3.8. Statistical Analysis

Statistical analysis was performed by using GraphPad Prism 8 software. Unless otherwise stated, the values for all samples under the different experimental conditions were averaged, and the standard deviation of the mean was calculated. Statistical differences between the two groups were assessed by Student’s test. A *p*-value of less than 0.05 was considered statistically significant.

### 3.9. Molecular Modeling

All molecular docking simulations were performed using SYBYL-X 2.1.1 (Certara, Radnor, PA, USA) with the Surflex-Dock module. The crystal structure of ClpP in complex with **ONC201** (PDB ID: 6DL7, resolution: 2.00 Å) used as the receptor model. The protein structure was prepared using the Biopolymer module. Hydrogen bonds were added, atom types were checked, and the protonation states of ionizable residues were assigned at pH 7.0. The co-crystallized ligand was used to define the docking pocket and then removed before docking. The receptor structure was energy-minimized using the AMBER7 FF99 force field with Gasteiger-Hückel charges until the energy gradient reached 0.005 kcal/(mol·Å). The ClpP activator-binding pocket was defined by generating a protomol based on the co-crystallized **ONC201**, with the binding region extended 5 Å around the ligand. Ligand structures were built in SYBYL-X and energy-minimized using the Tripos force field with Gasteiger-Hückel charges. During docking, 20 initial conformations were generated for each ligand. Docking poses were ranked according to the Surflex-Dock Total Score, an empirical scoring function approximating −log10(Kd). The final binding poses were selected by considering the Total Score, reasonable polar and hydrophobic interactions, and acceptable Crash values indicating no severe steric clashes. The redocking validation, RMSD values, docking scores, full Surflex Dock configuration parameters have been provided in the Supplementary Materials. Molecular interactions were analyzed and visualized using PyMOL 3.1.

## 4. Conclusions

Guided by the co-crystal structure of **ONC201** in complex with the ClpP protein, we applied a ring-opening-based molecular simplification strategy to modify the core scaffold and optimized the side arms. Through comprehensive structural optimization, we identified a series of novel piperidine-fused imidazolone scaffold-based ClpP activators. Structure-activity relationship (SAR) studies ultimately identified compound **CLPP-3036** as a highly potent ClpP activator. Its agonistic activity against the ClpP protein reached 6.02 nM, representing a 240-fold improvement compared to **ONC201**. Importantly, **CLPP-3036** exhibited IC_50_ values of 1.13 nM and 1.52 nM against the MV4-11 and MOLM-13 AML tumor cell lines, respectively, making it a highly potent ClpP agonist for hematological tumor cells. In the Raji cell line, **CLPP-3036** showed up to a 1260-fold (Raji IC_50_ = 9.80 nM) increase in antiproliferative activity compared to **ONC201**. In other solid tumor cell lines such as A375 and Mia PaCa-2, **CLPP-3036** also demonstrated significantly superior inhibitory activity over **ONC201**. Furthermore, at a low concentration, **CLPP-3036** facilitated the degradation of ClpP downstream proteins and robustly induced cell apoptosis in MV4-11 cells. Collectively, **CLPP-3036** can be utilized as a promising lead compound for further ClpP-targeted drug development.

## Figures and Tables

**Table 1:** Enzymatic Activity and Cell Growth Inhibition of Compounds **A1**–**A8** ^a^.

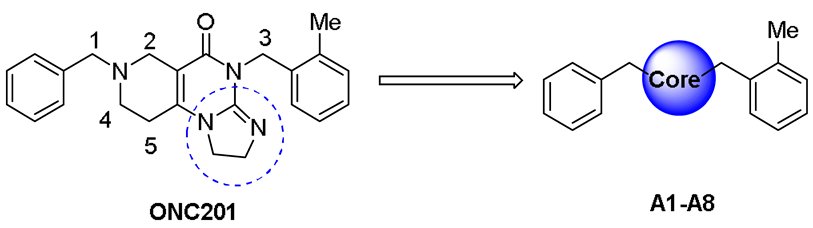			
Compd.	Compound Structures	EC_50_ (nM)	MV4-11 IC_50_ (nM)
**ONC201**	-	878.65 ± 56.45	265.30 ± 29.10
**A1**	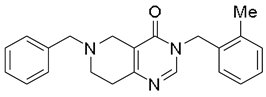	7126.0 ± 453.0	1159.0 ± 49.4
**A2**	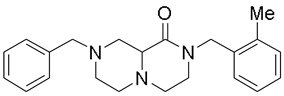	5559.5 ± 286.5	349.2 ± 36.9
**A3**	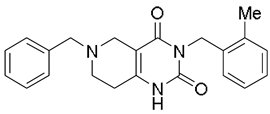	>20,000	>20,000
**A4**	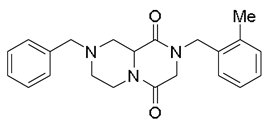	>20,000	>20,000
**A5**	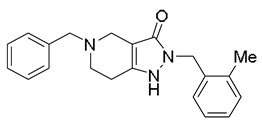	2960.5 ± 87.5	634.9 ± 45.3
**A6**	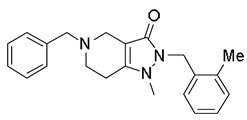	>20,000	>20,000
**A7**	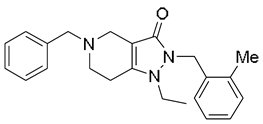	>20,000	>20,000
**A8**	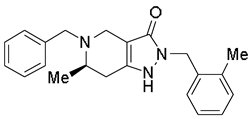	3839.5 ± 177.5	938.5 ± 45.7

^a^ Data are presented as the mean value of triplicate samples ± SD (*n* = 3 triplicate wells).

**Table 2:** Enzymatic Activity and Cell Growth Inhibition of Compounds **A9**–**A26** ^a^.

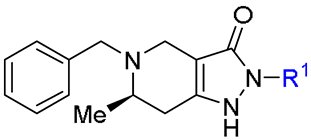							
Compd.	R^1^	EC_50_ (nM)	MV4-11 IC_50_ (nM)	Compd.	R^1^	EC_50_ (nM)	MV4-11 IC_50_ (nM)
**ONC201**	**-**	878.65 ± 56.45	265.30 ± 29.10	**A17**	** 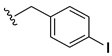 **	126.45 ± 4.95	38.60 ± 2.30
**A8**	** 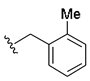 **	3839.50 ± 177.50	938.50 ± 45.70	**A18**	** 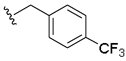 **	59.72 ± 2.33	41.00 ± 4.50
**A9**	** 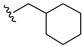 **	4092 ± 636	622.90± 80.10	**A19**	** 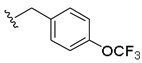 **	240.85 ± 10.45	133.00 ± 24.60
**A10**	** 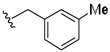 **	544.55 ± 12.05	321.20 ± 16.60	**A20**	** 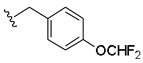 **	1188.50 ± 23.50	344.30 ± 19.60
**A11**	** 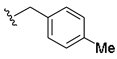 **	423.85 ± 7.15	229.40 ± 4.80	**A21**	** 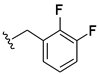 **	4505.00 ± 292.00	>20,000
**A12**	** 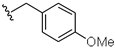 **	463.70 ± 0.80	327.00 ± 18.00	**A22**	** 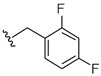 **	2313.00 ± 254.00	595.50 ± 40.30
**A13**	** 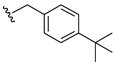 **	>20,000	>20,000	**A23**	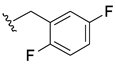	6571.00 ± 169.00	>20,000
**A14**	** 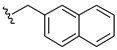 **	843.85 ± 428.15	135.10 ± 13	**A24**	** 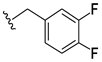 **	334.60 ± 45.50	591.90± 59.50
**A15**	** 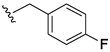 **	1096.50 ± 50.50	129.10 ± 6.20	**A25**	** 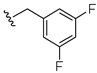 **	993.00 ± 77.00	351.90 ± 2.20
**A16**	** 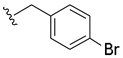 **	32.69 ± 1.80	13.30 ± 0.30	**A26**	** 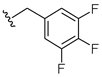 **	391.85 ± 4.85	115.80 ± 12.50

^a^ Data are presented as the mean value of triplicate samples ± SD (*n* = 3 triplicate wells).

**Table 3:** Enzymatic Activity and Cell Growth Inhibition of **A27**–**A37** ^a^.

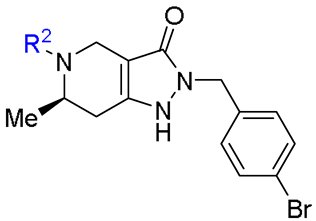							
Compd.	R^1^	EC_50_ (nM)	MV4-11 IC_50_ (nM)	Compd.	R^1^	EC_50_ (nM)	MV4-11 IC_50_ (nM)
**ONC201**	**-**	878.65 ± 56.45	265.30 ± 29.10	**A32**	** 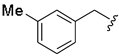 **	5.61 ± 0.25	4.80 ± 0.50
**A16**	** 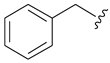 **	32.69 ± 1.80	13.30 ± 0.30	**A33**	** 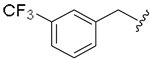 **	2.80 ± 0.24	1.70 ± 0.30
**A27**	** 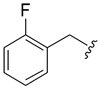 **	211.50 ± 6.20	254.80 ± 17.70	**A34**	** 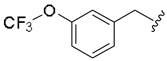 **	10.10 ± 0.37	82.10 ± 7.50
**A28**	** 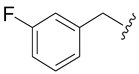 **	9.39 ± 0.04	10.70 ± 0.35	**A35**	** 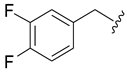 **	17.72 ± 1.33	10.10 ± 0.70
**A29**	** 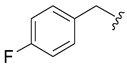 **	37.16 ± 2.50	2.70 ± 0.10	**A36** **(CLPP-3036)**	** 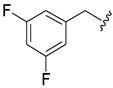 **	6.02 ± 0.34	1.13 ± 0.03
**A30**	** 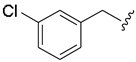 **	11.73 ± 1.35	4.60 ± 0.45	**A37**	** 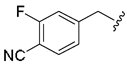 **	1111.0 ± 26.0	392.30 ± 44.10
**A31**	** 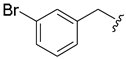 **	13.75 ± 0.34	3.90 ± 0.10				

^a^ Data are presented as the mean value of triplicate samples ± SD (*n* = 3 triplicate wells).

**Table 4:** IC_50_ Values of **CLPP-3036** Compounds for the Inhibition of the Proliferation of different cells ^a^.

Cell Lines	IC_50_ (nM)	
	CLPP-3036	ONC201
MV4-11	1.13 ± 0.03	265.27 ± 29.12
MOLM-13	1.52 ± 0.03	1795.33 ± 212.57
Raji	9.80 ± 1.05	12,383.67 ± 511.59
A375	6.83 ± 0.09	1156.33 ± 143.47
Mia PaCa-2	12.23 ± 1.31	6294.00 ± 747.04
H1299	13.11 ± 2.79	6724.33 ± 445.78

^a^ Data are presented as the mean value of triplicate samples ± SD (*n* = 3 triplicate wells).

## Data Availability

The original contributions presented in this study are included in the article/Supplementary Material. Further inquiries can be directed to the corresponding authors.

## Supplementary Materials

The following supporting information can be downloaded at: https://www.mdpi.com/article/10.3390/molecules31162916/s1, Figure S1: (A) Viability curves of Raji, A375, MIIA Paca-2, and H1299 cells treated with **CLPP-3036** at indicated concentrations for 72 h. (B) Viability curves of Raji, A375, MIIA Paca-2, and H1299 cells treated with **ONC201** at indicated concentrations for 72 h; Table S1: Enzymatic Activity and Cell Growth Inhibition of **S1** and **A16**; Figure S2: (A) Viability curves of MV4-11 cells treated with **S1** at indicated concentrations for 72 h. (B) Viability curves of MV4-11 cells treated with **A16** at indicated concentrations for 72 h. Figure S3: (A) Uncropped original western blot images of SDHB, NDUFA12, and α-Tubulin in MV4-11 cells treated with the indicated concentrations of **CLPP-3036** for 36 h. Figure S4: Redocking validation, RMSD values, docking scores. Figure S5: Surflex-Dock parameters. ^1^H/^13^C/^19^F NMR, HPLC traces, HRMS data of representative compounds (PDF); The Original Western Blot Images (PDF); Predicted_binding_mode_compound_**A1** (PDB); Predicted_binding_mode_compound_**CLPP-3036** (PDB); Molecular string files for all of the final target compounds (CSV).

## Abbreviations

SAR	Structure–Activity Relationships
EC_50_	Median effect concentration
IC_50_	Half maximal inhibitory concentration
TFA	Trifluoroacetic Acid
Cs_2_CO_3_	Cesium Carbonate
K_2_CO_3_	Potassium Carbonate
EDCI	N-(3-dimethylaminopropyl)-n**’**-ethylcarbodiimide hydrochloride
HOBT	1-Hydroxybenzotriazole
AML	Acute Myeloid Leukemia
SDHB	Succinate Dehydrogenase Complex Iron Sulfur Subunit B
NDUFA12	NADH:ubiquinone oxidoreductase subunit A12
Boc_2_O	Di-tert-butyl dicarbonate
NMR	Nuclear Magnetic Resonance
HRMS	High-Resolution Mass Spectrometry
ESI	Electrospray Ionization
HPLC	High-Performance Liquid Chromatography
SBDD	Structure-based drug design
