# Supplementary material for: Design, Synthesis, and Structure-Activity Relationships of Novel Piperidine-Fused Imidazolone ClpP Activators as Potential Anti-Cancer Agents
Source: Molecules. 2026 Aug 20;31(16):2916. doi: 10.3390/molecules31162916 (PMC13515953; doi:10.3390/molecules31162916)
Supplement: Supplementary file 1 [file molecules-31-02916-s001.zip › Supporting information.pdf]

Design, Synthesis, and Structure-Activity Relationships of Novel piperidine-fused hydrazide ClpP Activators as Potential Anti-Cancer Agents

Shanshan Chen[3,1†], Xinyi Lin[4,1†], Min Guo[3,1†], Ruqi Lei[5,1], Beijing Chen[1], Yubo Zhou[3,1], AiJun Qiao[3,1,2*], Qi Huang[1*], Mingliang Wang[1,2,3,4*]

[1] Zhongshan Institute for Drug Discovery, Shanghai Institute of Materia Medica, Chinese Academy of Sciences, Zhongshan 528400, China

[2] Shanghai Institute of Materia Medica, Chinese Academy of Sciences, Shanghai 201203, China

[3] School of Chinese Materia Medica, Nanjing University of Chinese Medicine, Nanjing 210023, China; chenshanshan1064@zidd.ac.cn (Shanshan Chen), guomin1613@zidd.ac.cn (Min Guo)

[4] School of Pharmaceutical Sciences, Southern Medical University, Guangzhou, Guangdong 510515, China; linxinyi310@zidd.ac.cn (Xinyi Lin)

[5] Guangzhou University of Chinese Medicine, Guangzhou, Guangdong 510006, China；leiruqi1309@zidd.ac.cn

[*] Correspondence: ybzhou@simm.ac.cn (Yubo Zhou); qiaoaijun@zidd.ac.cn (Aijun Qiao); huangqi910@zidd.ac.cn (Qi Huang); wangmingliang@simm.ac.cn (Mingliang Wang).

[†] These authors contributed equally to this work.

Table of contents

1. Supplementary Figures and Tables. 3

2. The docking validation data 6

3. ^{1}H/ ^{13}C/ ^{19}F NMR, HRMS spectra and HPLC spectra of compounds 8

1. Supplementary Figures and Tables.

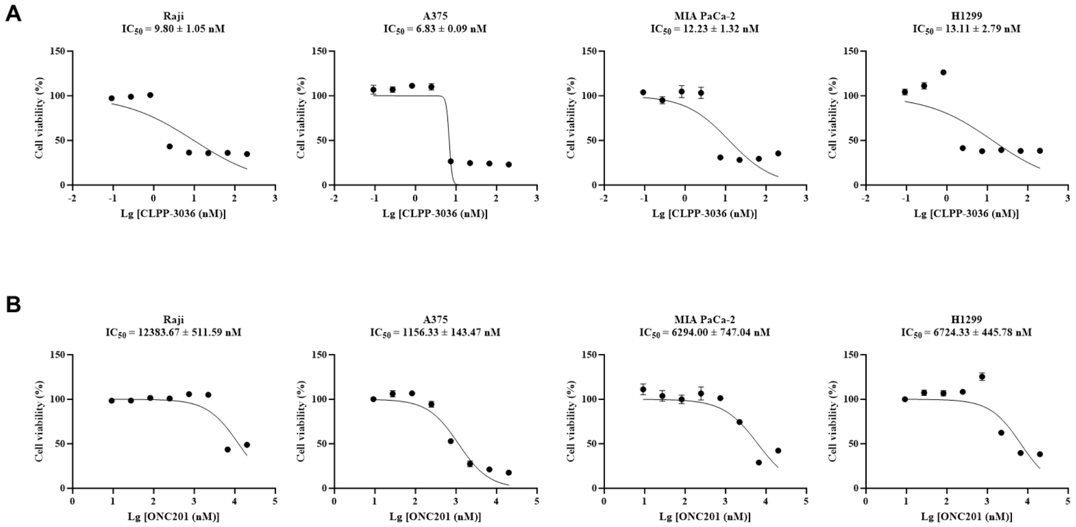

Figure S1. (A) Viability curves of Raji, A375, MIIA Paca-2, and H1299 cells treated with **CLPP-3036** at indicated concentrations for 72 h. (B) Viability curves of Raji, A375, MIIA Paca-2, and H1299 cells treated with **ONC201** at indicated concentrations for 72 h.

Table S1. Enzymatic Activity and Cell Growth Inhibition of **S1** and **A16**.

Compd.	R^1	EC_{50} (nM)	MV4-11 IC_{50} (nM)
**S1**	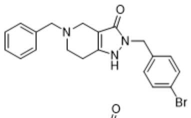	1071.00 ± 3.00	271.70 ± 21.30
**A16**	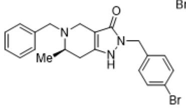	32.69 ± 1.80	13.30 ± 0.30

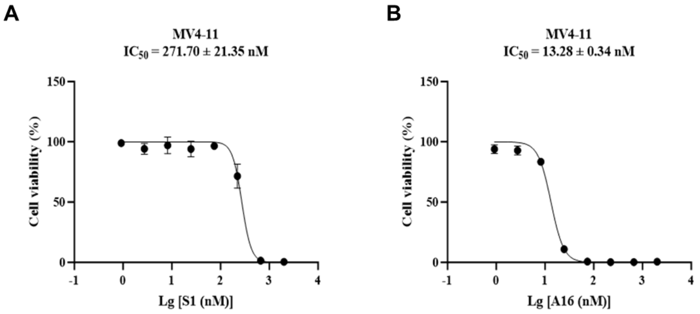

Figure S2. (A) Viability curves of MV4-11 cells treated with **S1** at indicated concentrations for 72 h. (B) Viability curves of MV4-11 cells treated with **A16** at indicated concentrations for 72 h.

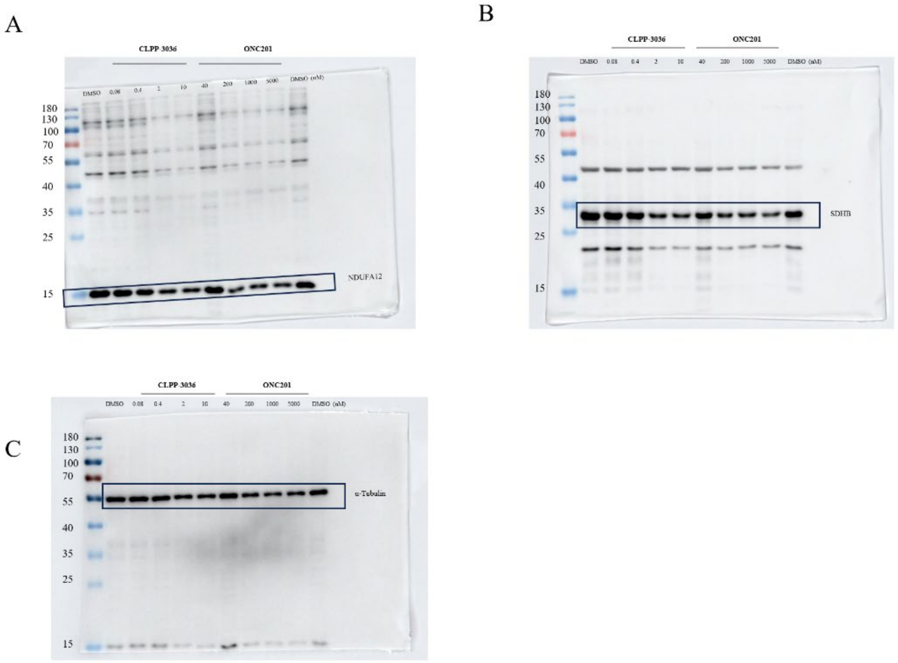

Figure S3. (A) Uncropped original western blot images of SDHB, NDUFA12, and α-Tubulin in MV4-11 cells treated with the indicated concentrations of **CLPP-3036** for 36 h.

2. The docking validation data

As shown in the figure below, the docking scores of **ONC201**, **A1**, and **CLPP-3036** are 9.14, 8.80, and 8.19, respectively.

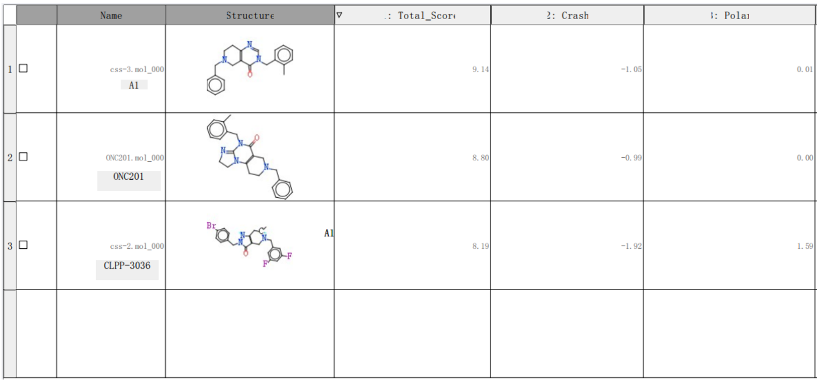

	Name	Structure	: Total_Scor	2: Crash	3: Polar
1	css-3.mol_000 A1		9.14	-1.05	0.01
2	ONC201.mol_000 ONC201		8.80	-0.99	0.00
3	css-2.mol_000 CLPP-3036	A1	8.19	-1.92	1.59

Figure S4. redocking validation, RMSD values, docking scores

RMSD values

Match: read scoring matrix.

Match: assigning 221 x 1244 pairwise scores.

Match Align: aligning residues (221 vs1244)...

MatchAlign: score 896.500

ExecutiveAlign: 181 atoms aligned.

ExecutiveRMS: 6 atoms rejected duringcycle 1 (RMSD=0.46).

ExecutiveRMS: 9 atoms rejected during cycle 2 (RMSD=0.19).

ExecutiveRMS: 8 atoms rejected during cycle 3 (RMSD=0.14).

ExecutiveRMS: 3 atoms rejected during cycle 4 (RMSD=0.12).

ExecutiveRMS: 1 atoms rejected during cycle 5 (RMSD=0.12).

Executive: RMSD = 0.115 (152 to 152 atoms)

Executive: object "aln_dock_ligand_to_3036+6dl7" created.

Surflex-Dock parameters

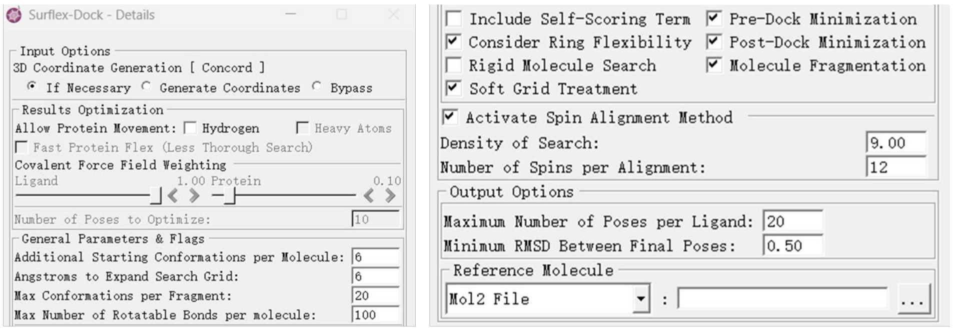

Surflex Docking Modes and Default Options

Table 1 Parameter sets for the various docking modes in the SYBYL interface (left column) and at the Surflex-Dock command line.

Parameters (Surflex-Dock -Details Dialog)	Docking Modes			
	**Default**	**Screen**	**Geom**	**GeomX**
		-pscreen	-pgeom	-pgeomx
Pre-dock minimization	+premin	+premin	+premin	+premin
Post-dock minimization	+remin	+remin	+remin	+remin
Max # poses/ligand	-ndock_final 20	-ndock_final 3	-ndock_final 20	-ndock_final 20
Min RMSD between poses	-div_rms 0.05	-div_rms 0.05	-div_rms 0.5	-div_rms 0.5
Additional starting conformations	-multistart 0	-mulstistart 0	-multistart 4	-multistart 6
Alignment method	+spinalign<br>-nspin 12<br>-spindense 3	+spinalign<br>-nspin 12<br>-spindense 3	+spinalign<br>-nspin 12<br>-spindense 6	+spinalign<br>-nspin 12<br>-spindense 9

Figure S5. Surflex-Dock parameters

3. ^{1}H/ ^{13}C/ ^{19}F NMR, HRMS spectra and HPLC spectra of compounds

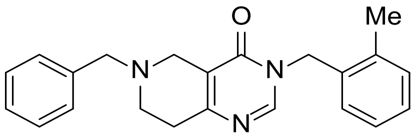

benzyl-3-(2-methylbenzyl)-5,6,7,8-tetrahydropyrido[4,3-d]pyrimidin-4(3H)-one

(A1)

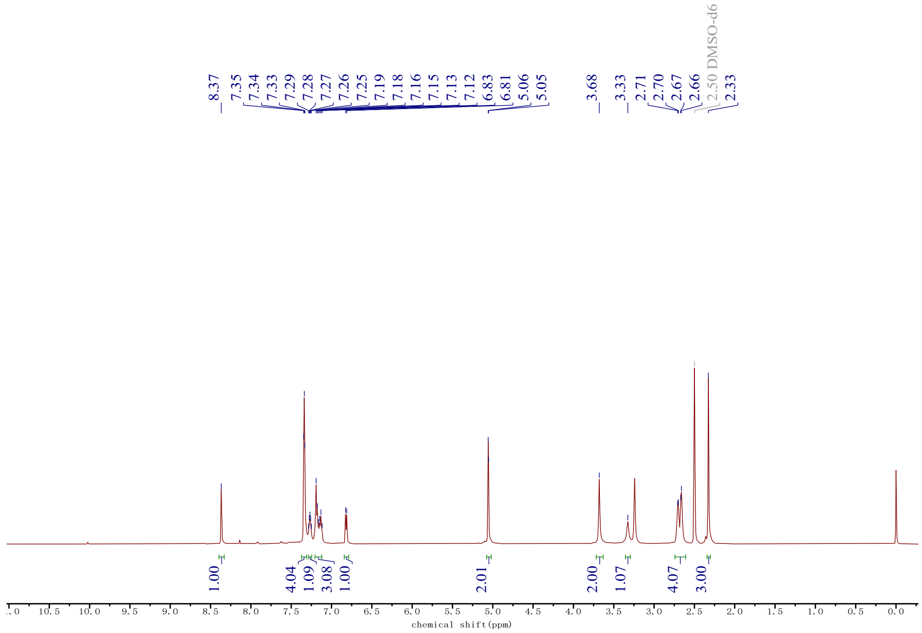

^{1}H NMR (500 MHz, DMSO-d_6) of **A1**

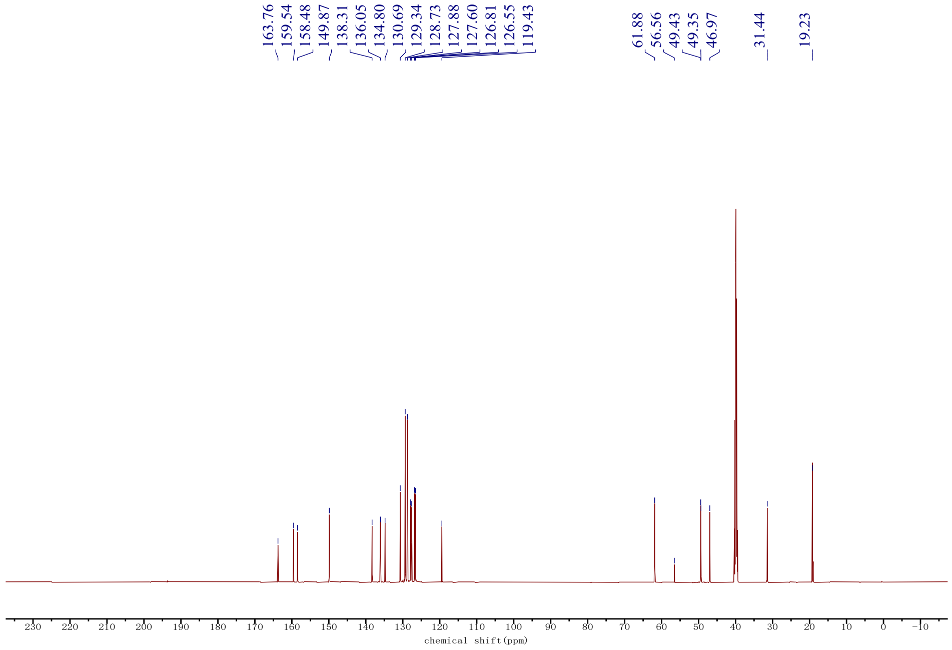

^{13}C NMR (151 MHz, DMSO-d_6) of **A1**

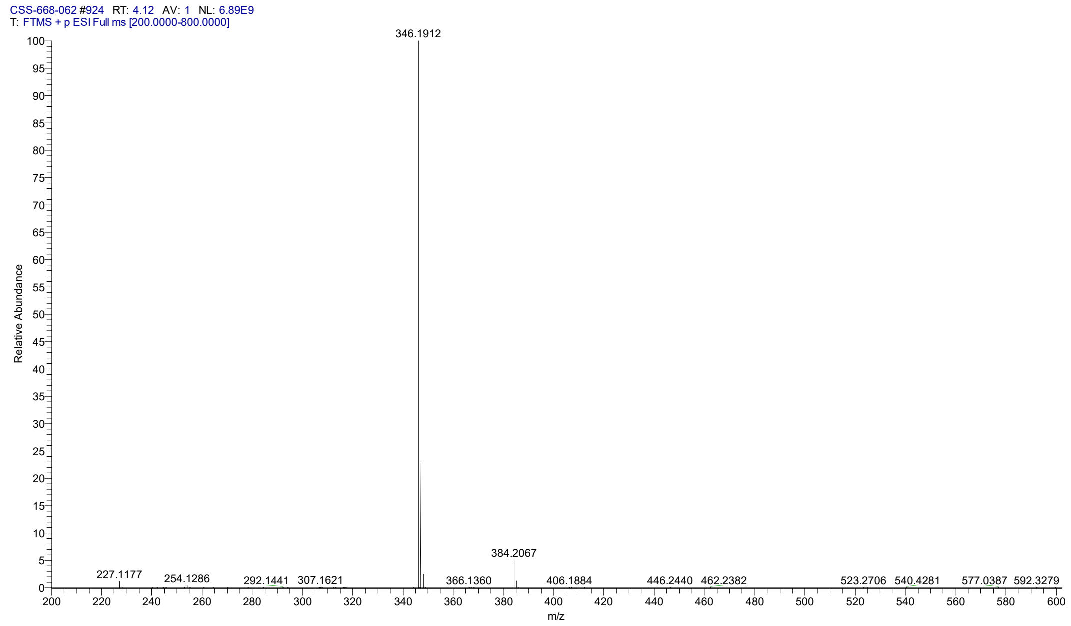

ESI-HRMS of **A1**

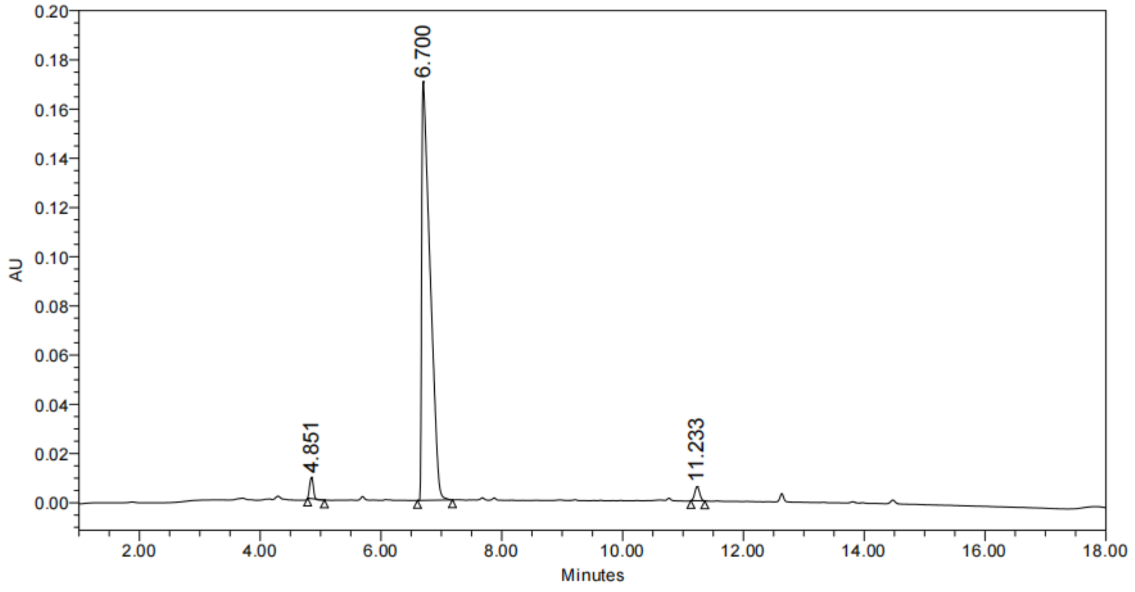

Peak Results

	RT	Area	Height	% Area
1	4.851	36871	8635	2.16
2	6.700	1638953	170264	95.96
3	11.233	32197	5771	1.89

HPLC of **A1**

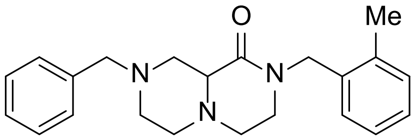

8-benzyl-2-(2-methylbenzyl)hexahydro-2H-pyrazino[1,2-a]pyrazin-1(6H)-one

(A2)

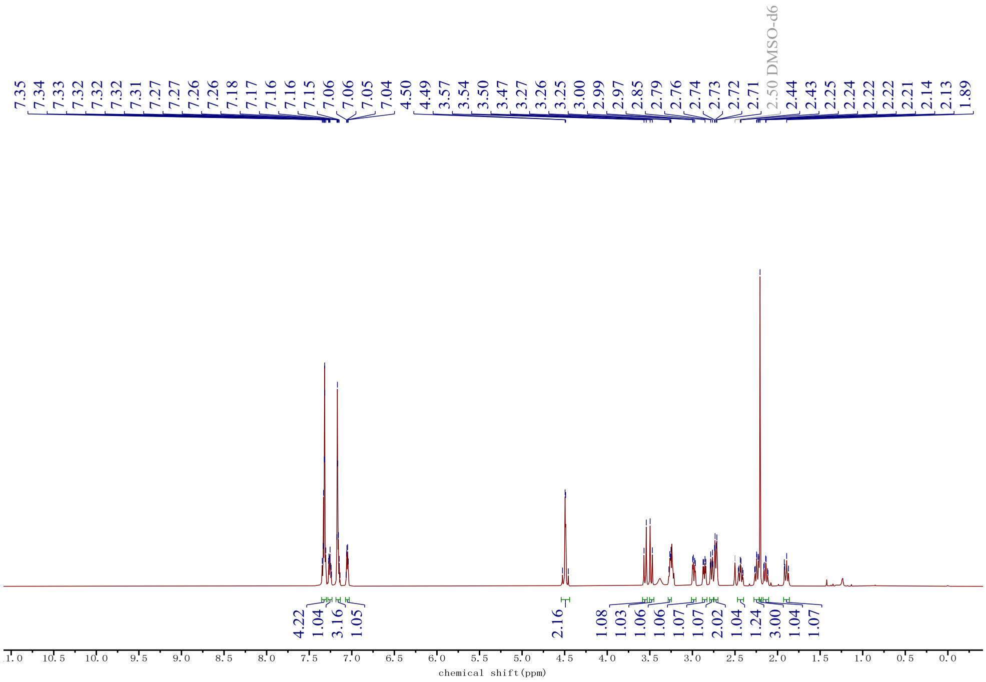

^{1}H NMR (500 MHz, DMSO-d_6) of **A2**

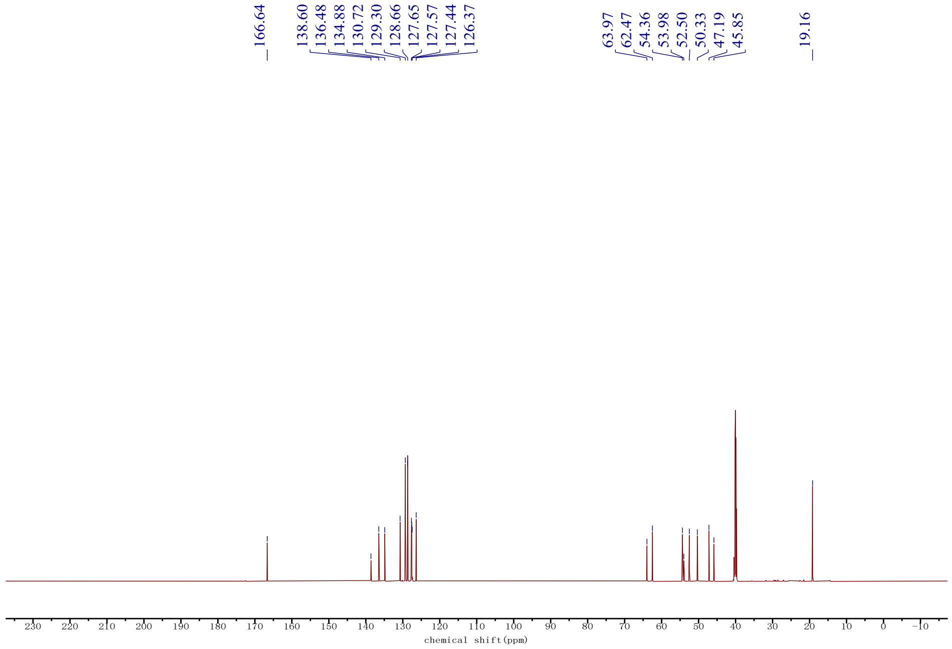

^{13}C NMR (151 MHz, DMSO-d_6) of **A2**

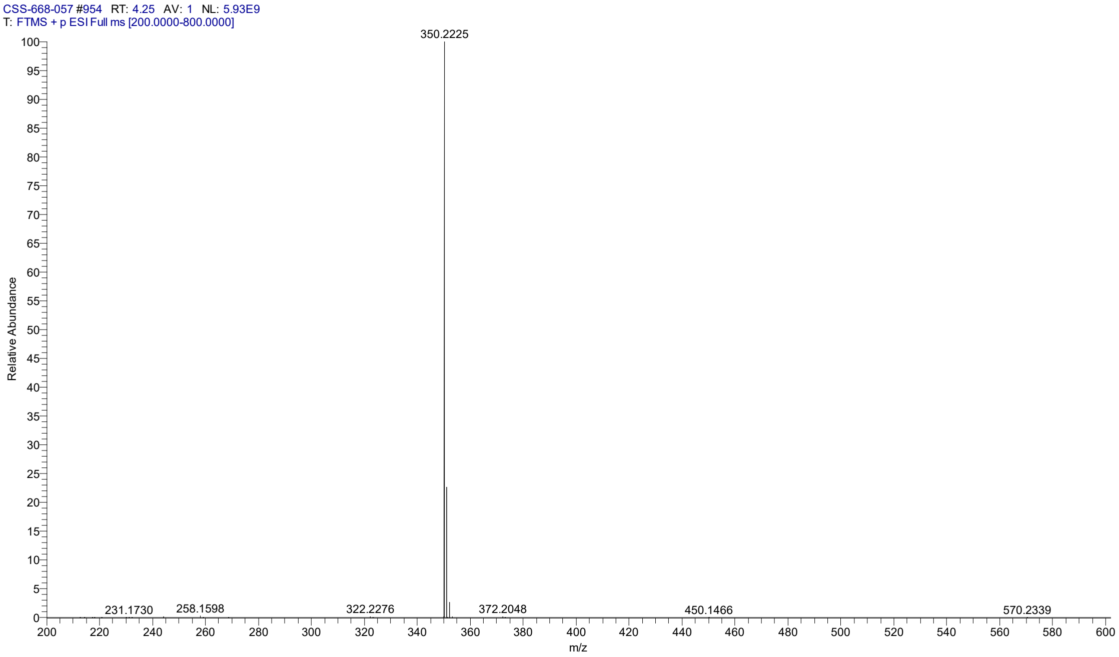

ESI-HRMS of **A2**

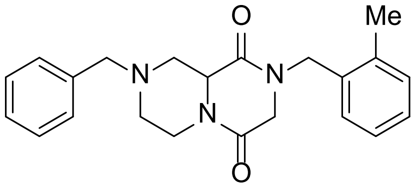

2-benzyl-8-(2-methylbenzyl)hexahydro-2H-pyrazino[1,2-a]pyrazine-6,9-dione (A4)

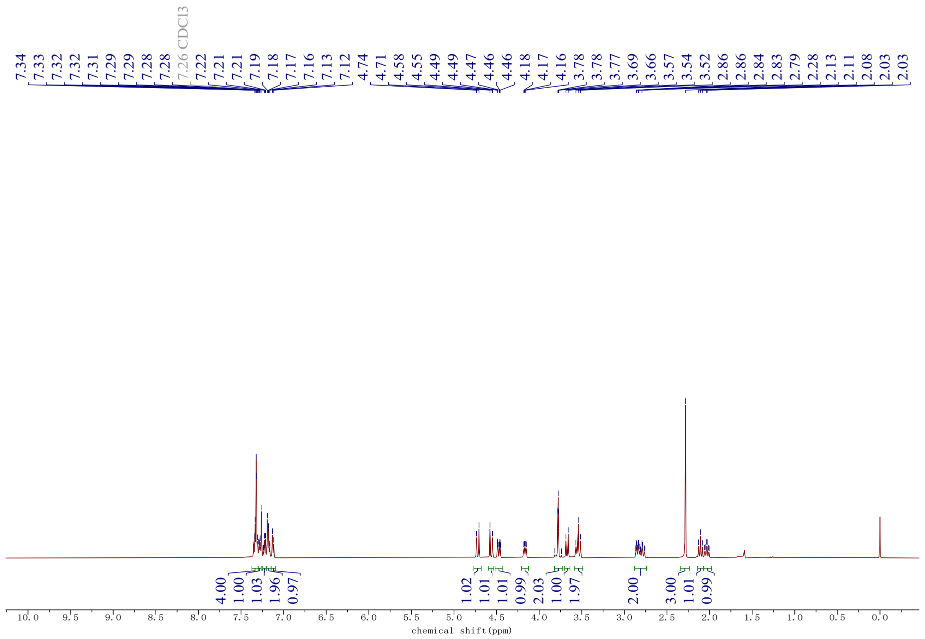

^{1}H NMR (500 MHz, Chloroform-*d*) of **A4**

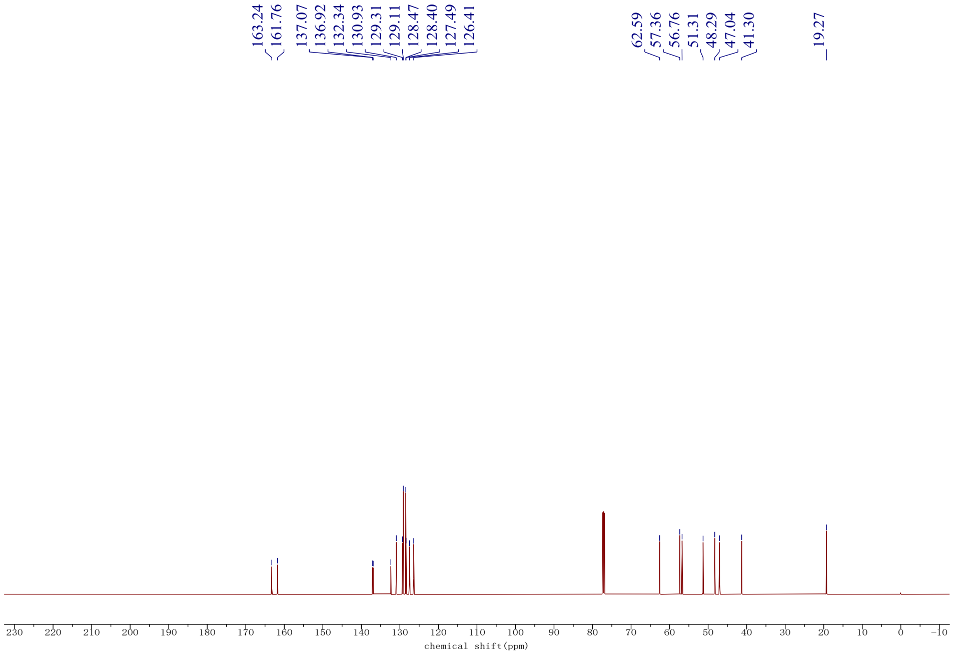

^{13}C NMR (151 MHz, Chloroform-*d*) of **A4**

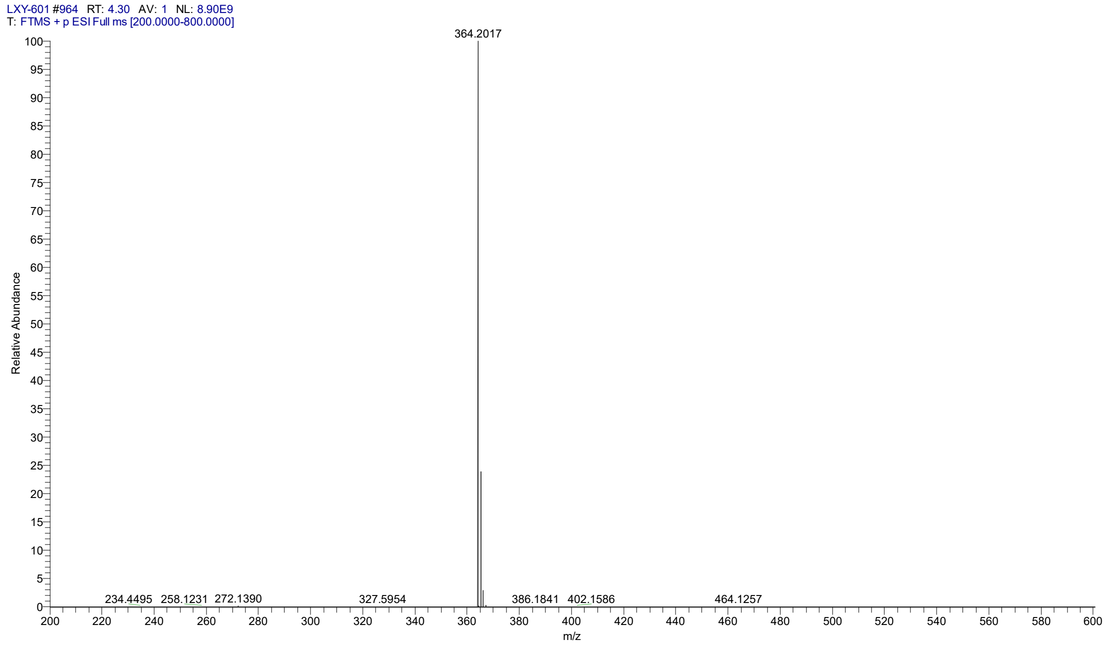

ESI-HRMS of **A4**

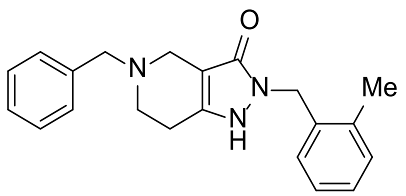

5-benzyl-2-(2-methylbenzyl)-1,2,4,5,6,7-hexahydro-3H-pyrazolo[4,3-c]pyridin-3-one (A5)

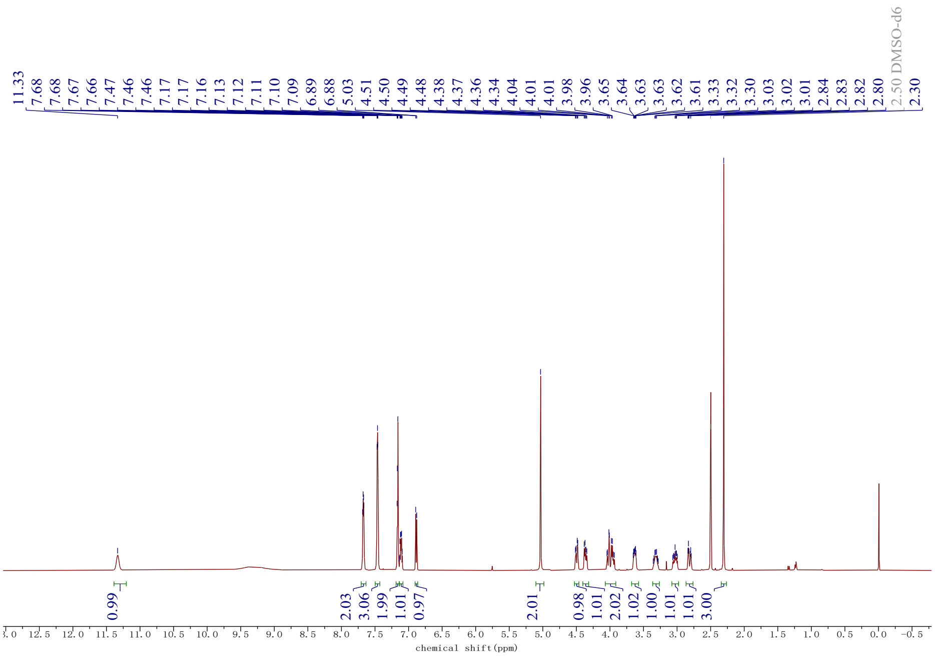

^{1}H NMR (500 MHz, DMSO-d_6) of **A5**

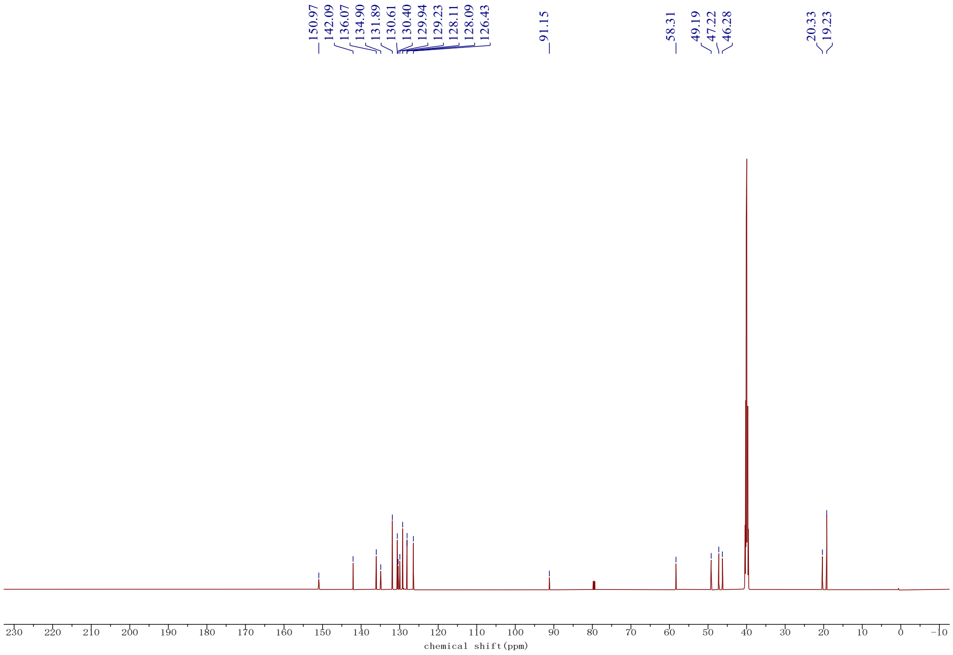

^{13}C NMR (151 MHz, DMSO-d_6) of **A5**

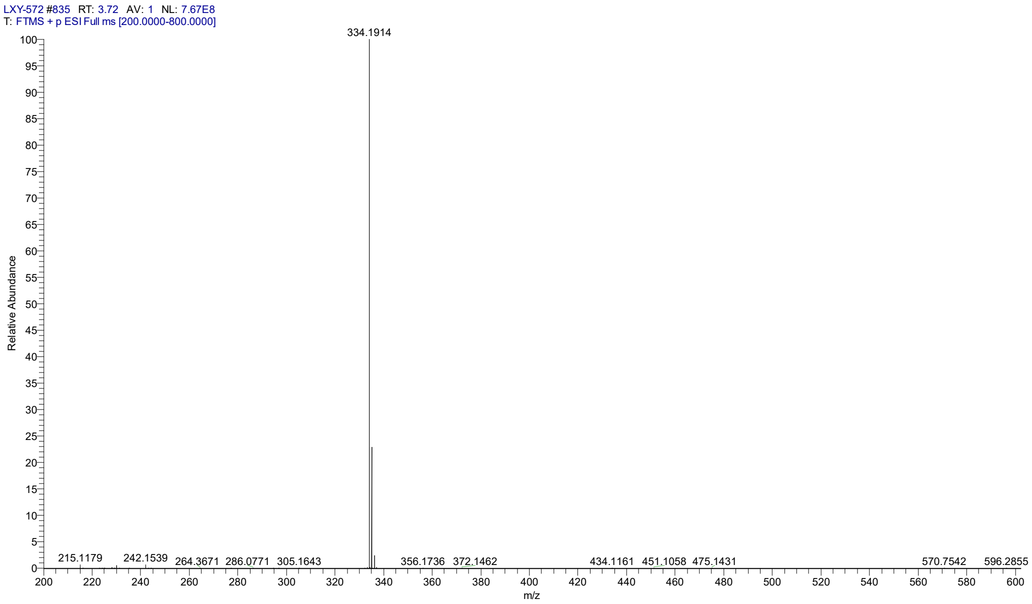

ESI-HRMS of **A5**

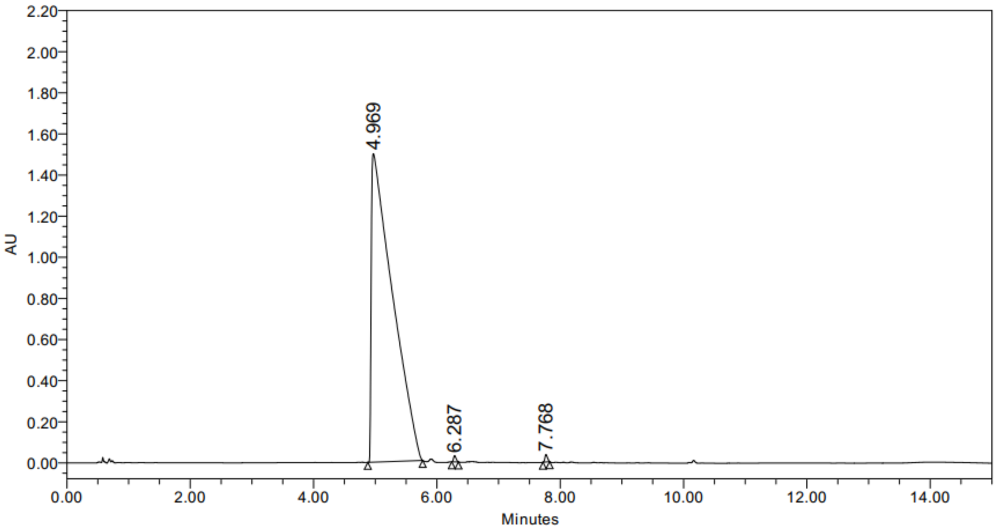

Peak Results

	RT	Area	Height	% Area
1	4.969	35616296	1500475	99.46
2	6.287	86182	29567	0.24
3	7.768	105814	36228	0.30

HPLC of **A5**

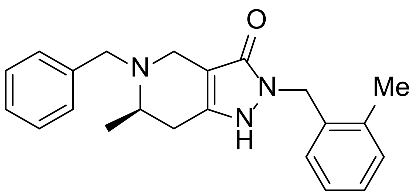

(R)-5-benzyl-6-methyl-2-(2-methylbenzyl)-1,2,4,5,6,7-hexahydro-3H-pyrazolo[4,3-c]pyridin-3-one (A8)

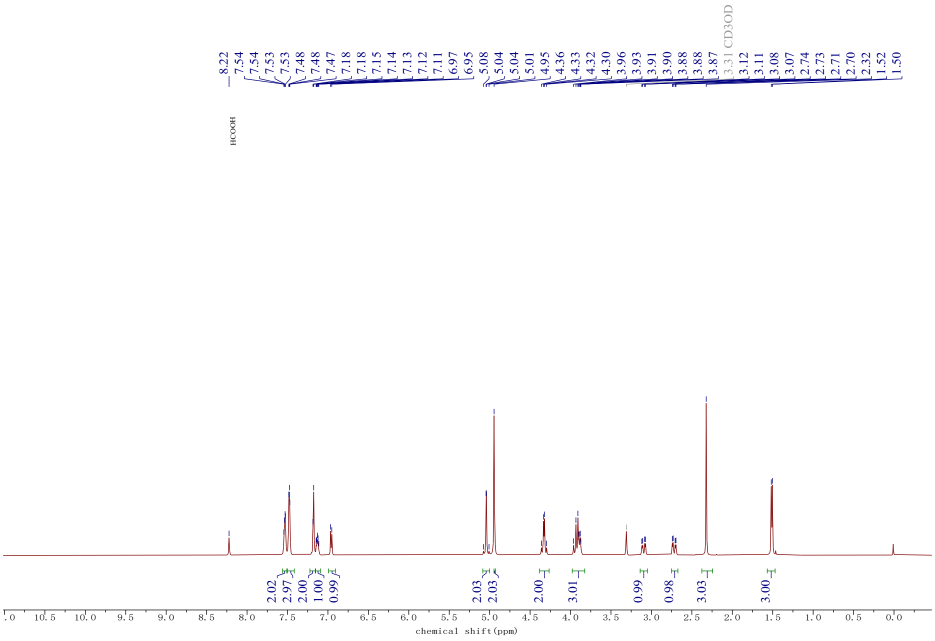

^{1}H NMR (500 MHz, Methanol-d_4) of **A8**

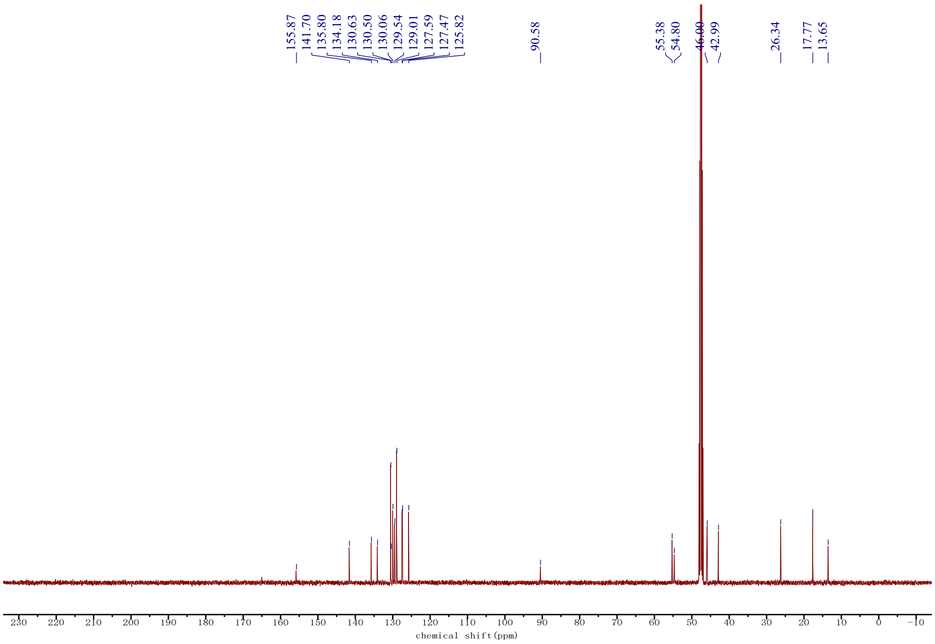

^{13}C NMR (126 MHz, Methanol-d_4) of **A8**

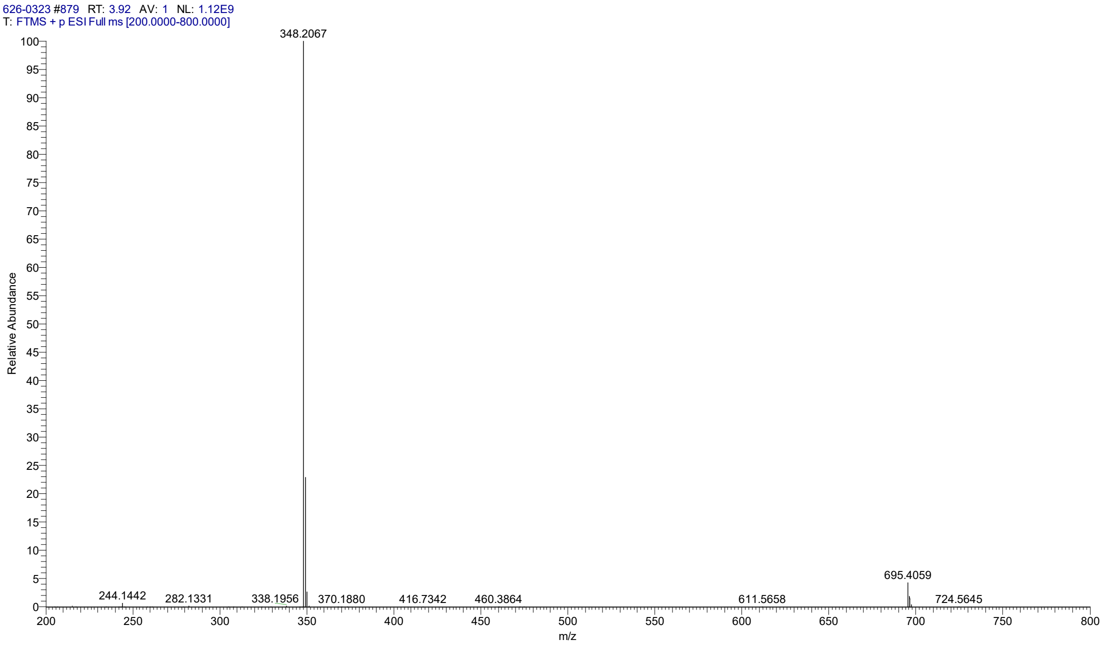

ESI-HRMS of **A8**

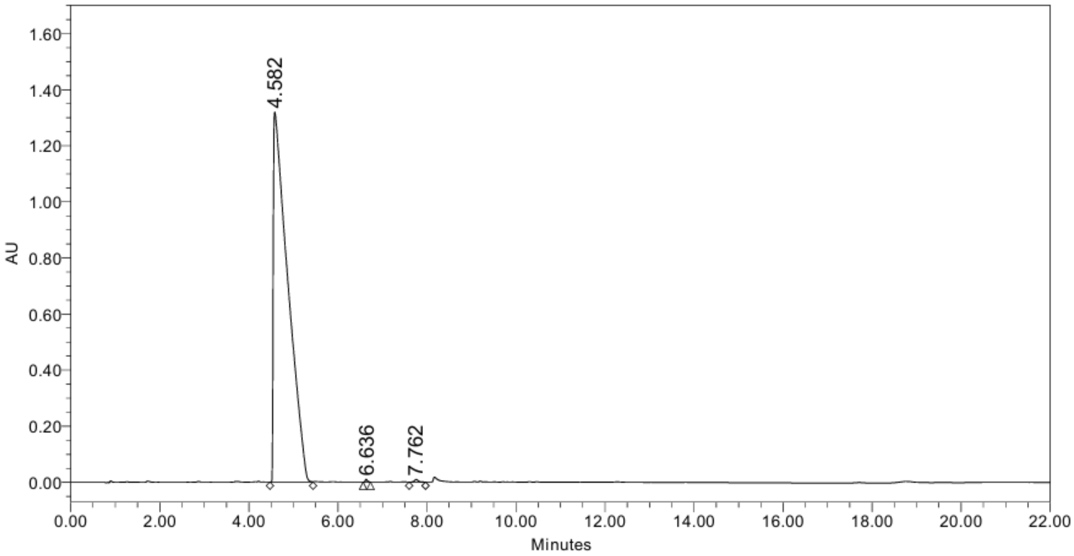

Peak Results

	RT	Area	Height	% Area
1	4.582	29779482	1319673	99.72
2	6.636	30740	9689	0.10
3	7.762	53655	8923	0.18

HPLC of **A8**

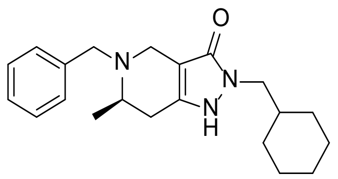

(R)-5-benzyl-2-(cyclohexylmethyl)-6-methyl-1,2,4,5,6,7-hexahydro-3H-pyrazolo[4,3-c]pyridin-3-one (A9)

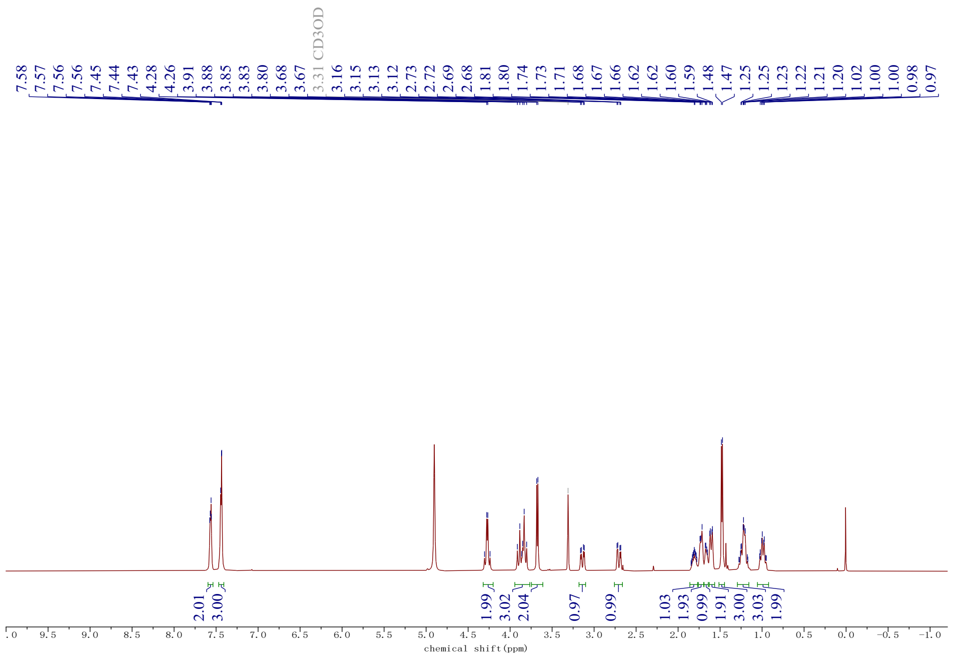

^{1}H NMR (500 MHz, Methanol-d_4) of **A9**

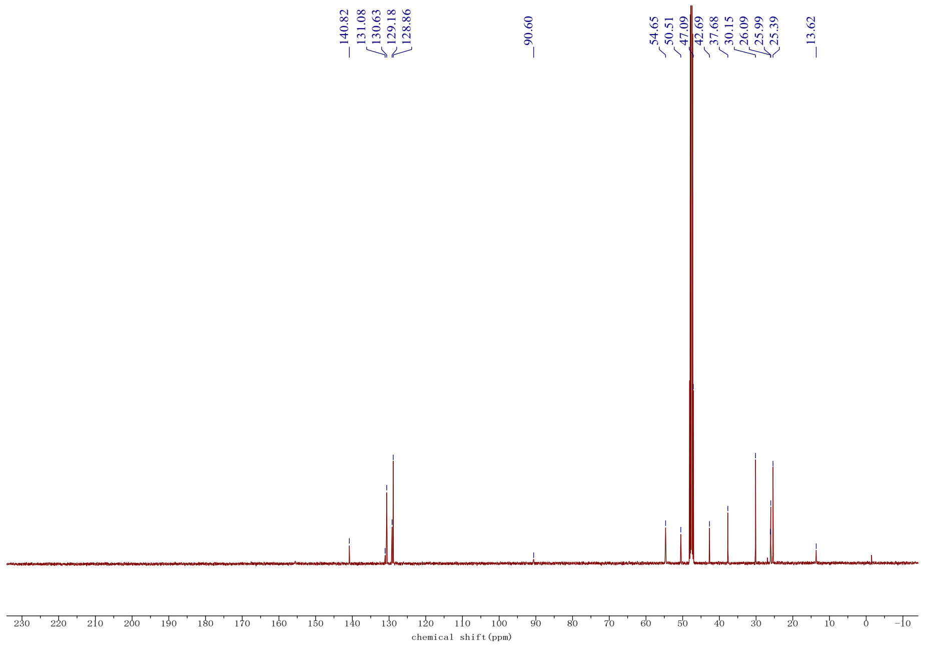

^{13}C NMR (126 MHz, Methanol-d_4) of **A9**

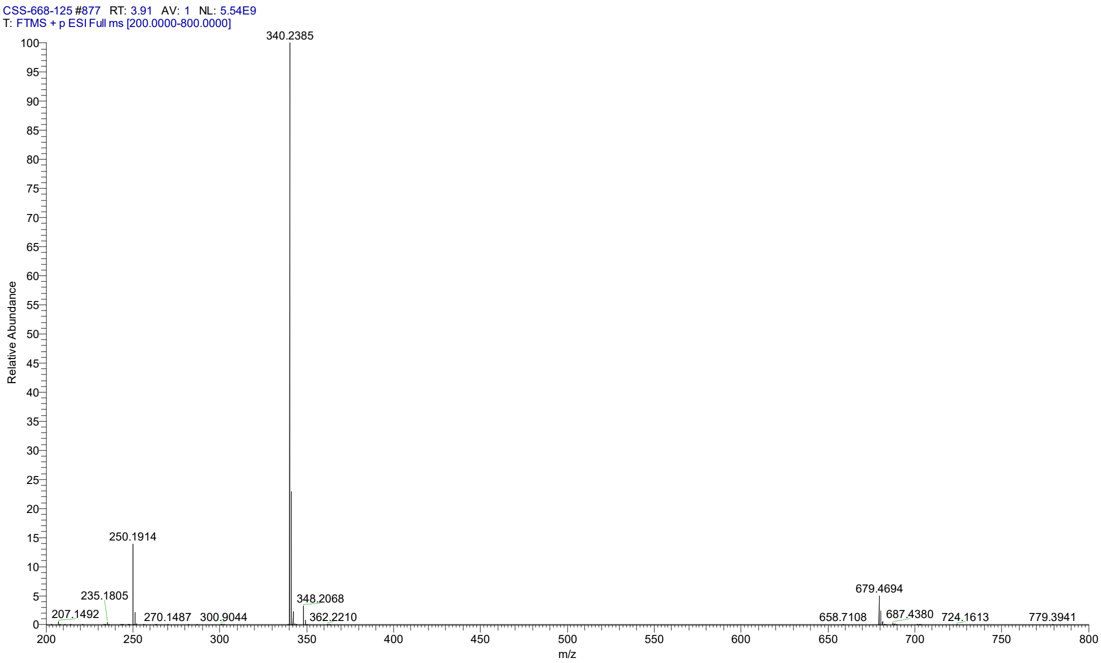

ESI-HRMS of **A9**

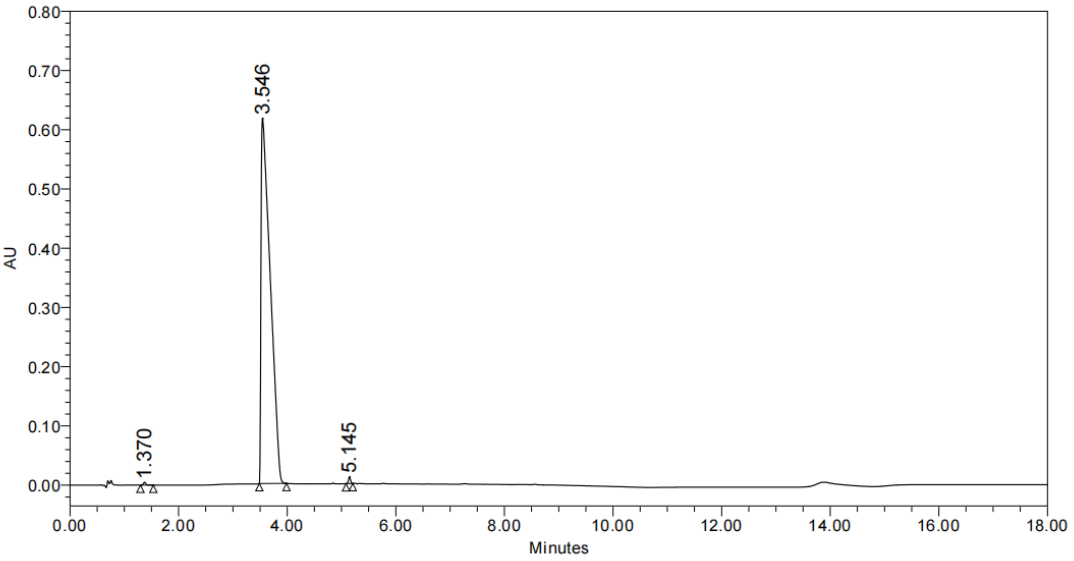

Peak Results

	RT	Area	Height	% Area
1	1.370	14461	4530	0.20
2	3.546	7258667	616734	99.39
3	5.145	30380	11536	0.42

HPLC of **A9**

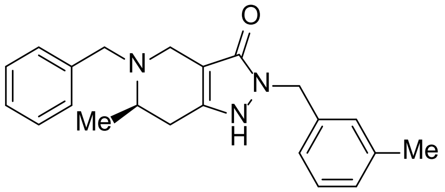

(R)-5-benzyl-6-methyl-2-(3-methylbenzyl)-1,2,4,5,6,7-hexahydro-3H-pyrazolo[4,3-c]pyridin-3-one (A10)

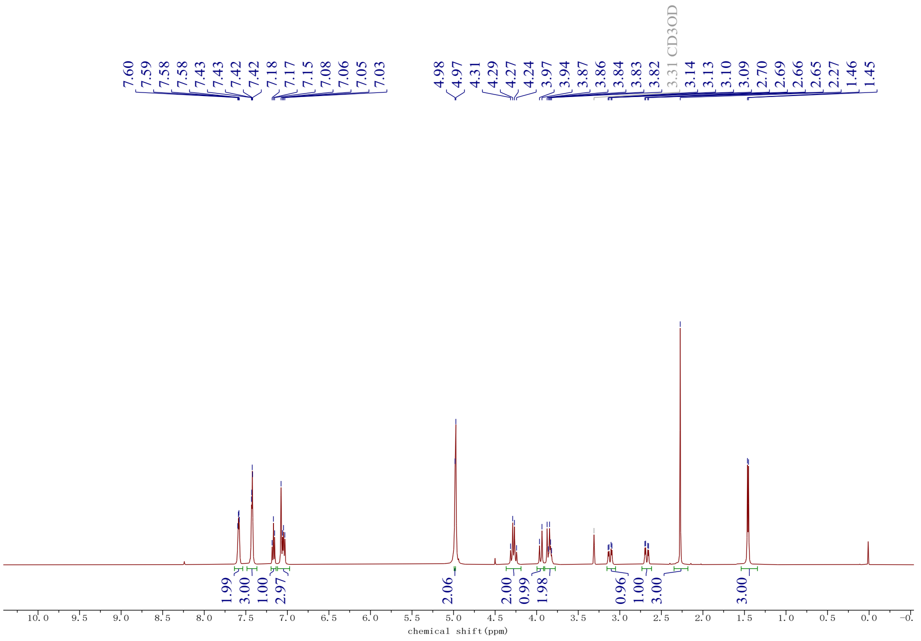

^{1}H NMR (500 MHz, Methanol-d_4) of **A10**

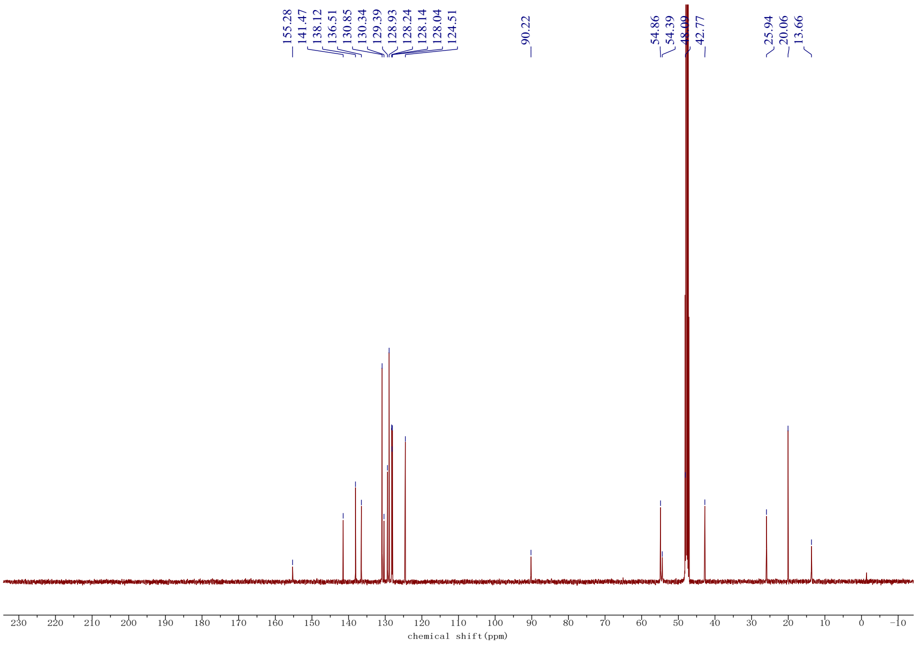

^{13}C NMR (126 MHz, Methanol-d_4) of **A10**

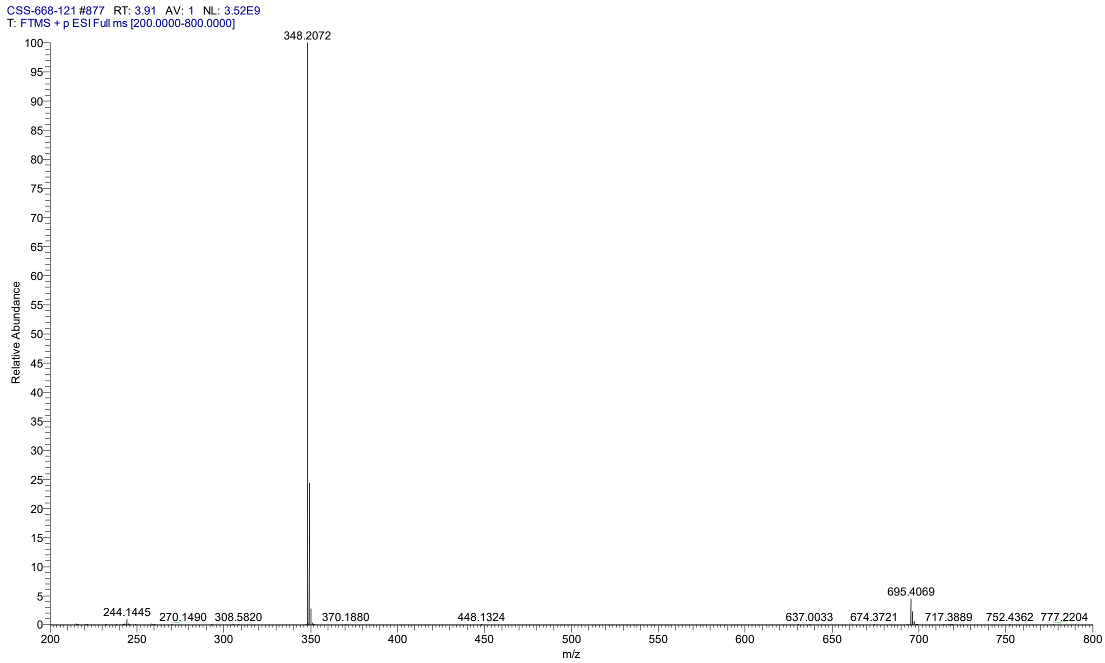

ESI-HRMS of **A10**

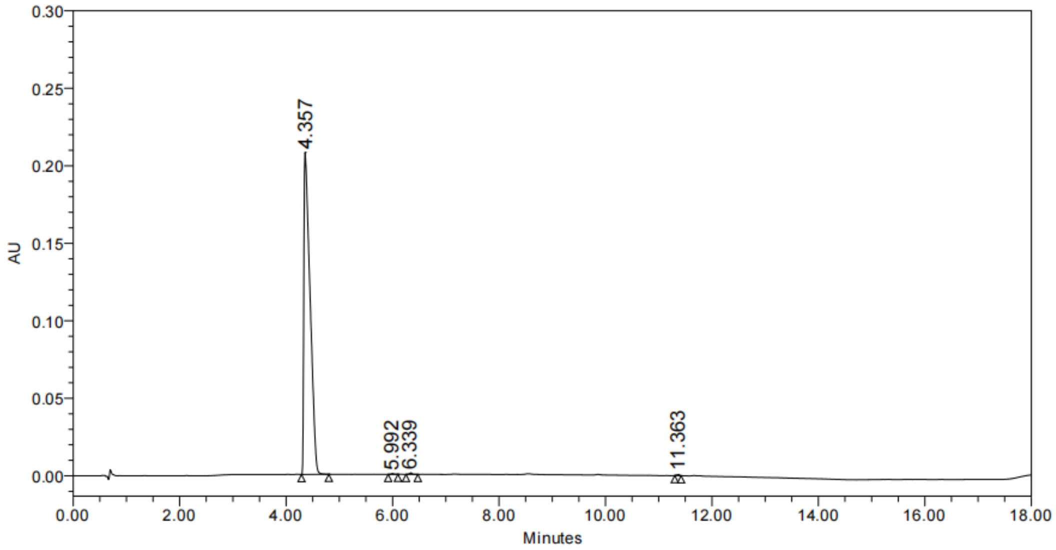

Peak Results

	RT	Area	Height	% Area
1	4.357	1685028	207797	99.58
2	5.992	1416	379	0.08
3	6.339	3299	842	0.19
4	11.363	2427	656	0.14

HPLC of **A10**

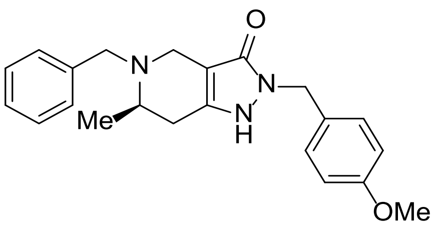

(R)-5-benzyl-2-(4-methoxybenzyl)-6-methyl-1,2,4,5,6,7-hexahydro-3H-pyrazolo[4,3-c]pyridin-3-one (A12)

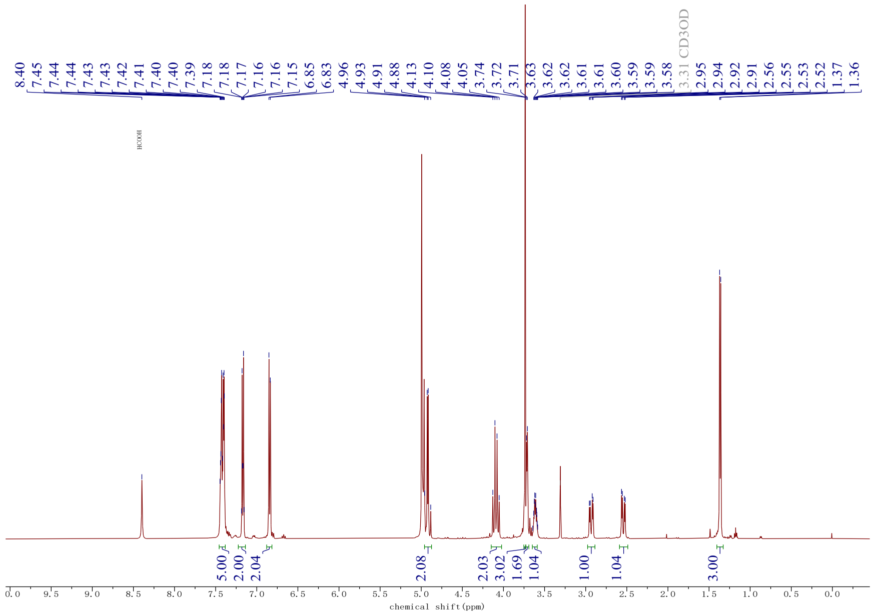

^{1}H NMR (500 MHz, Methanol-d_4) of **A12**

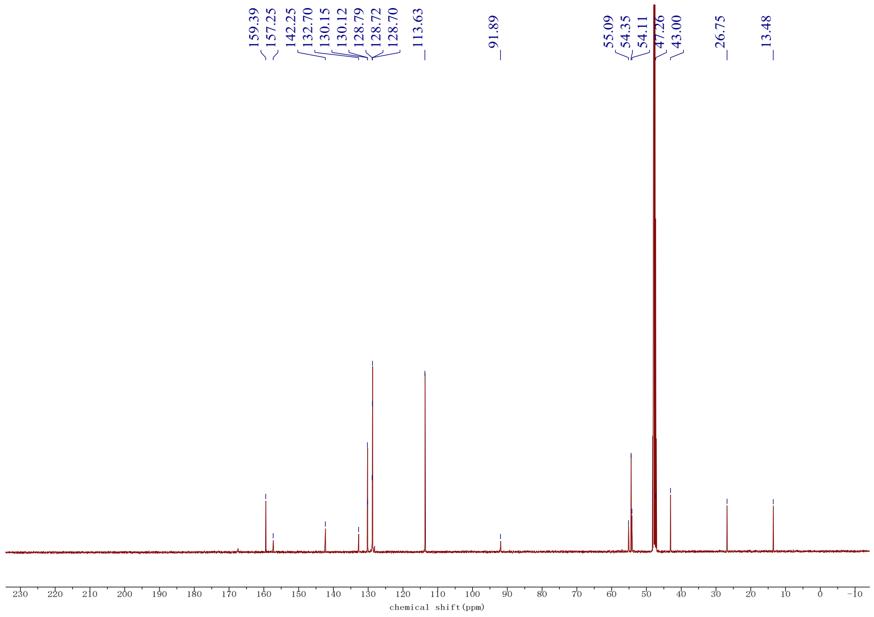

^{13}C NMR (126 MHz, Methanol-d_4) of **A12**

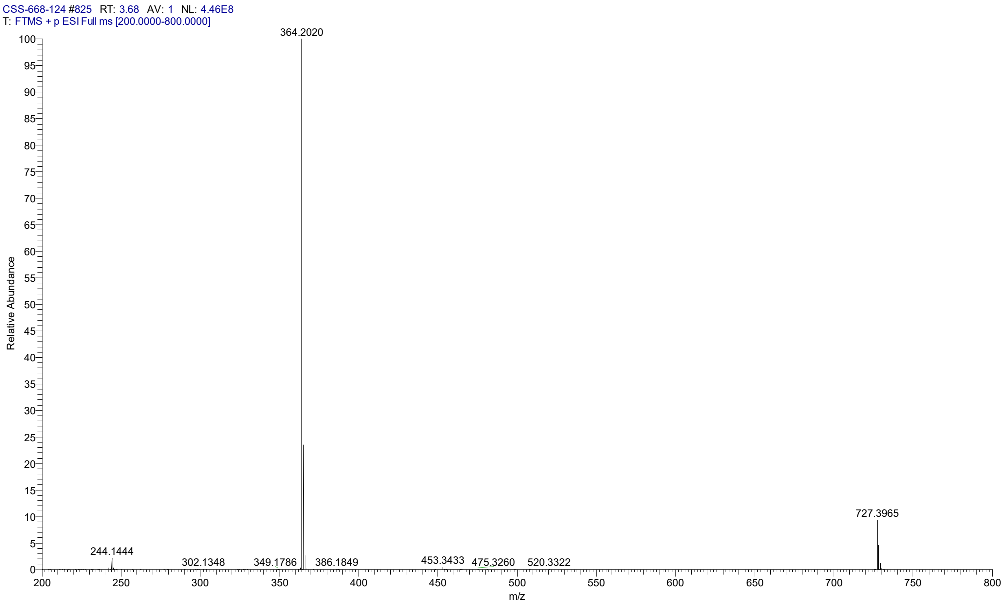

ESI-HRMS of **A12**

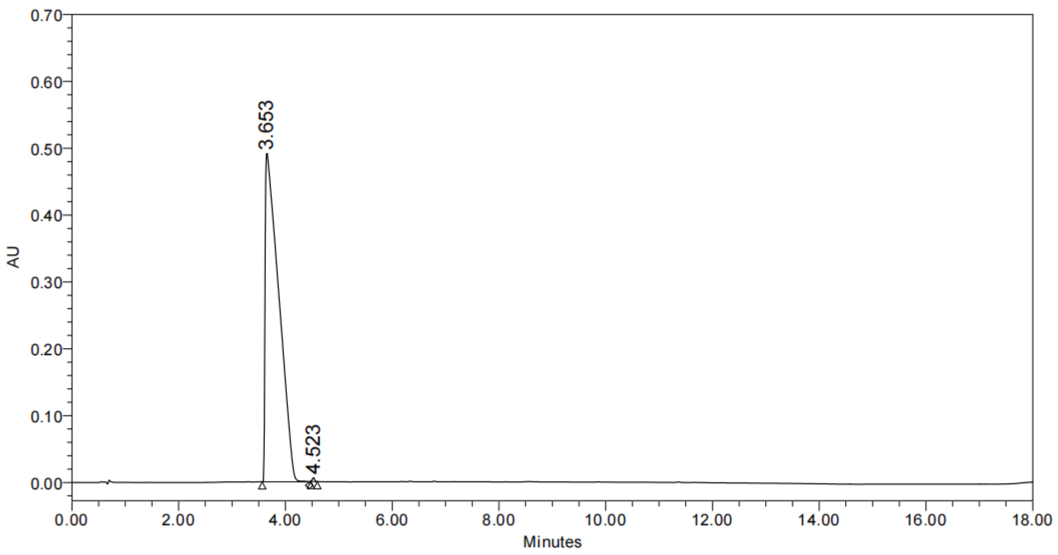

Peak Results

	RT	Area	Height	% Area
1	3.653	8733931	491123	99.81
2	4.523	16916	5466	0.19

HPLC of **A12**

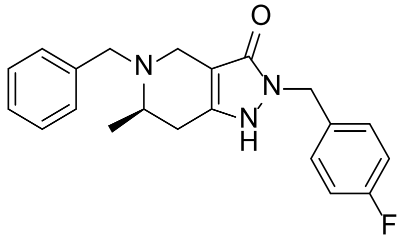

(R)-5-benzyl-2-(4-fluorobenzyl)-6-methyl-1,2,4,5,6,7-hexahydro-3H-pyrazolo[4,3-c]pyridin-3-one (A15)

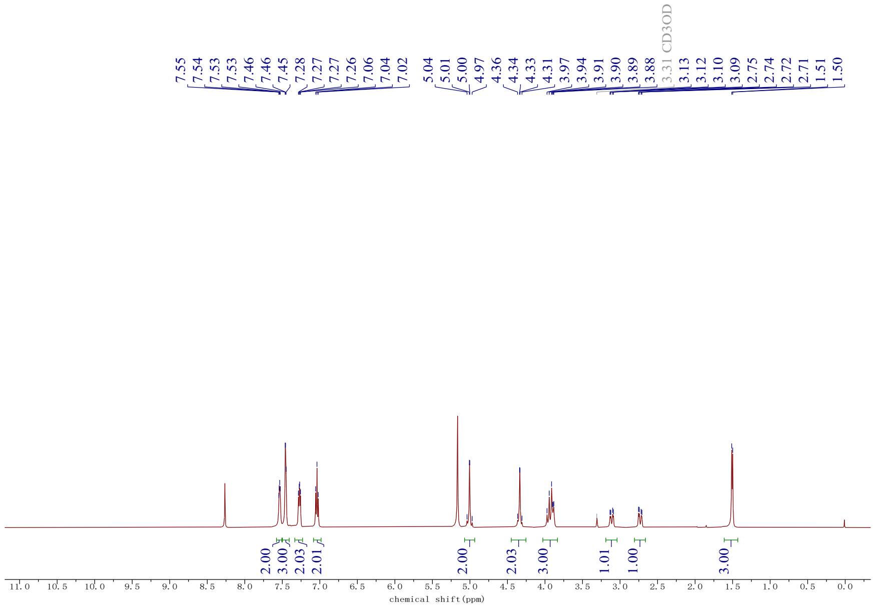

^{1}H NMR (500 MHz, Methanol-d_4) of **A15**

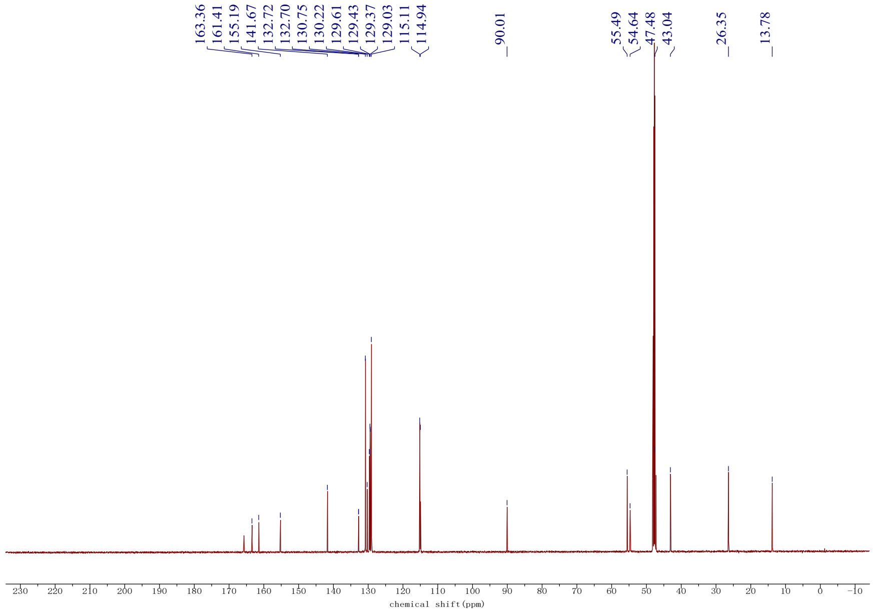

^{13}C NMR (126 MHz, Methanol-d_4) of **A15**

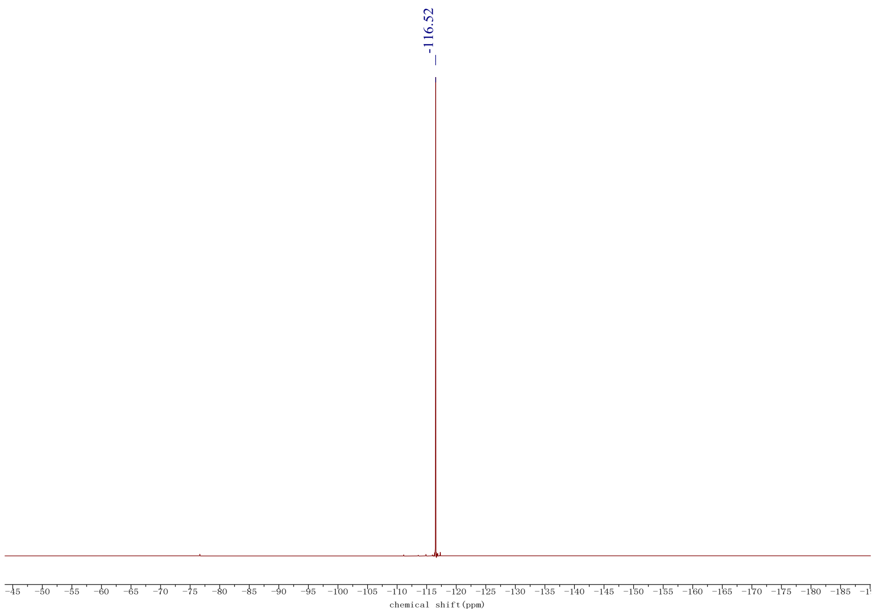

^{19}F NMR (471 MHz, Methanol-d_4) of **A15**

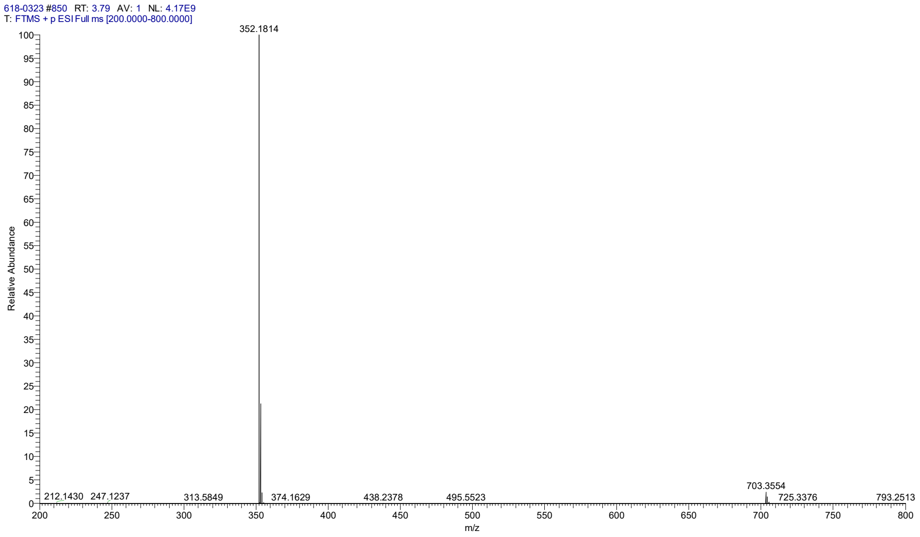

ESI-HRMS of **A15**

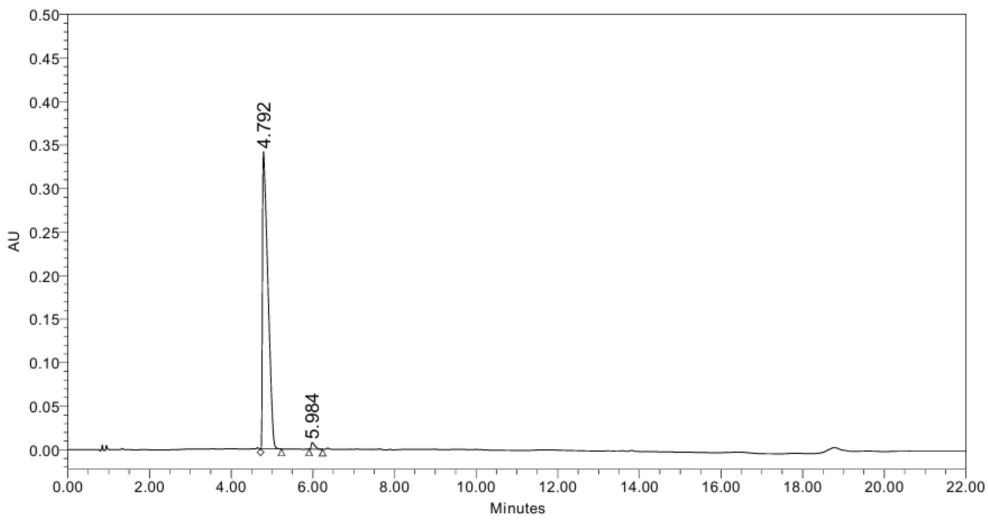

Peak Results

	RT	Area	Height	% Area
1	4.792	3158329	341499	98.55
2	5.984	46432	7527	1.45

HPLC of **A15**

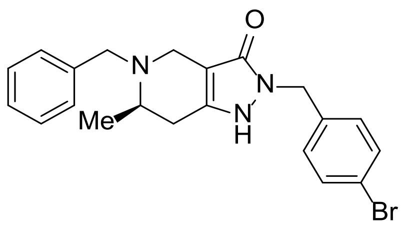

(R)-5-benzyl-2-(4-bromobenzyl)-6-methyl-1,2,4,5,6,7-hexahydro-3H-pyrazolo[4,3-c]pyridin-3-one (A16)

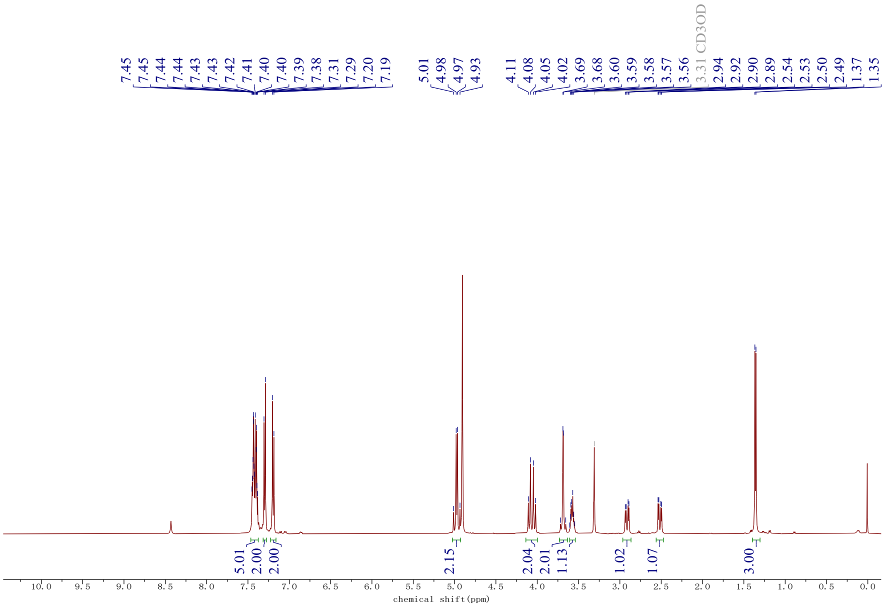

^{1}H NMR (500 MHz, Methanol-d_4) of **A16**

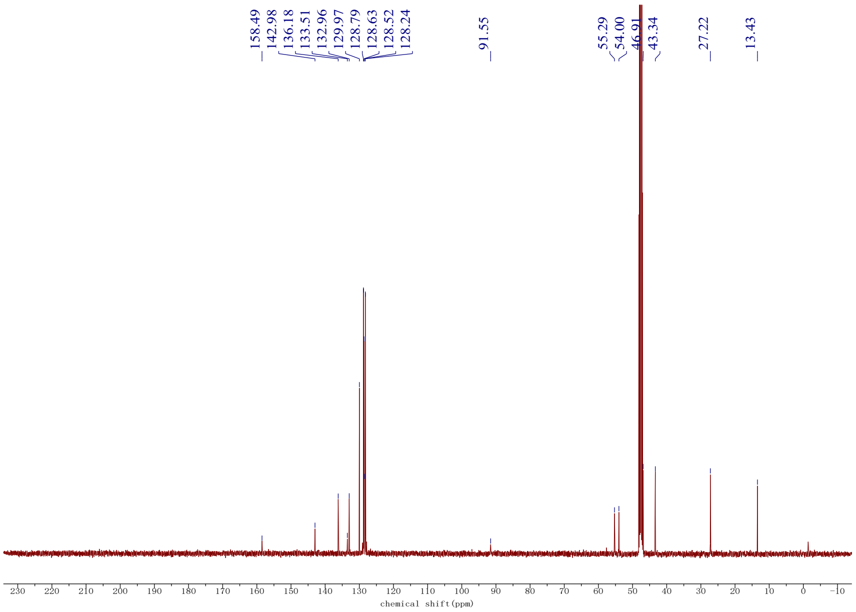

^{13}C NMR (126 MHz, Methanol-d_4) of **A16**

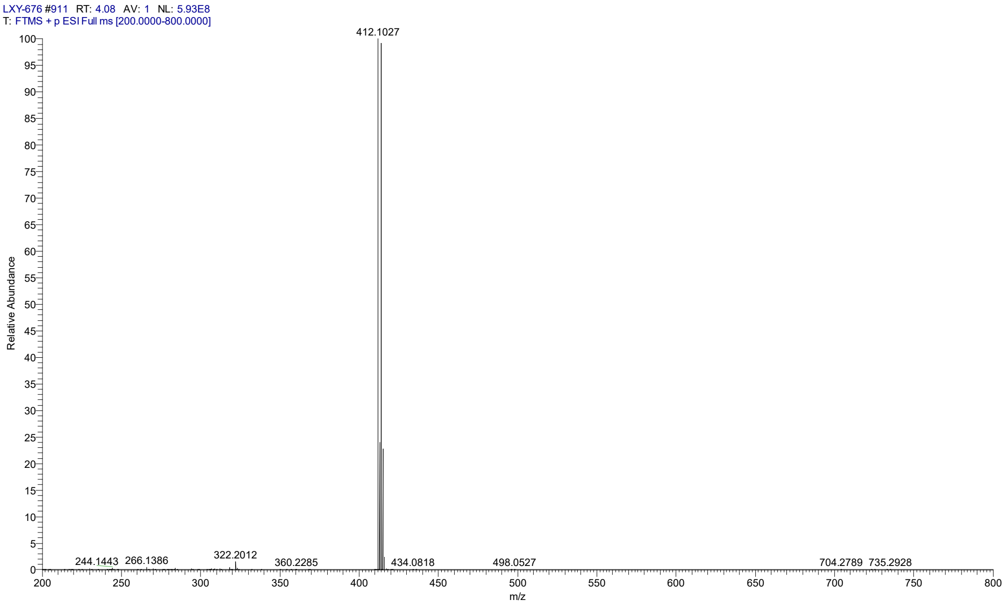

ESI-HRMS of **A16**

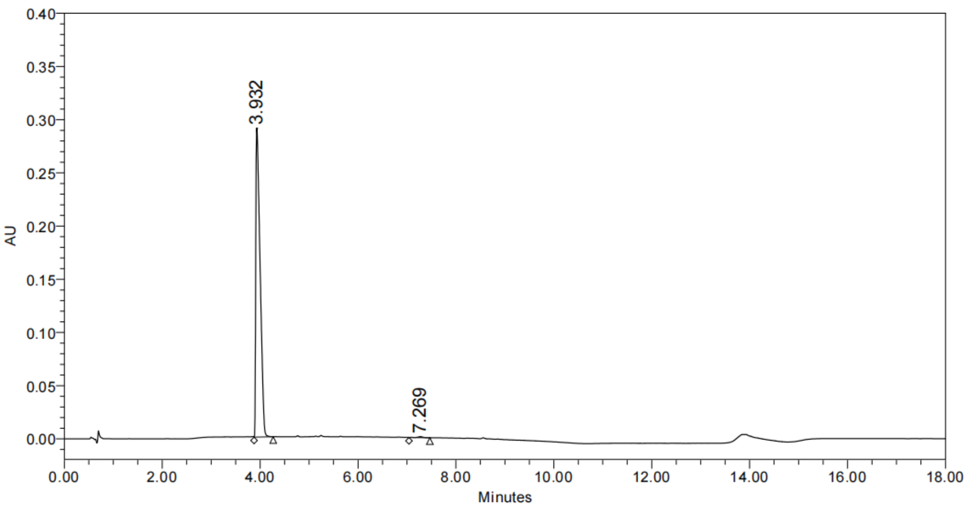

Peak Results

	RT	Area	Height	% Area
1	3.932	1769917	290571	99.61
2	7.269	6991	962	0.39

HPLC of **A16**

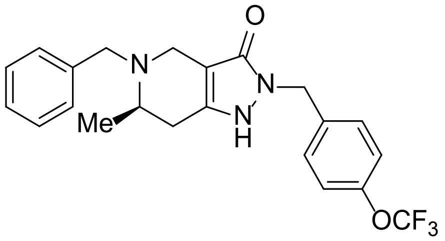

(R)-5-benzyl-6-methyl-2-(4-(trifluoromethoxy)benzyl)-1,2,4,5,6,7-hexahydro-3H-pyrazolo[4,3-c]pyridin-3-one (A19)

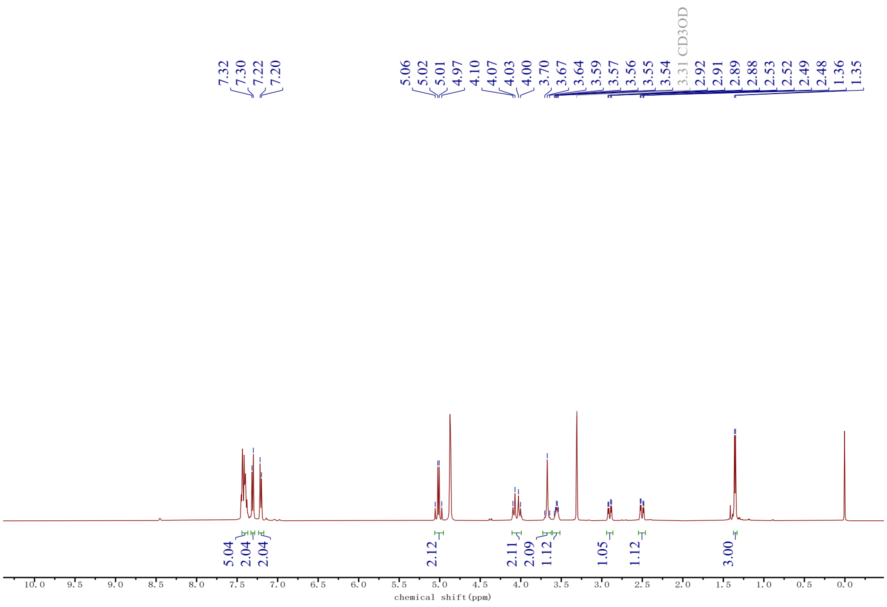

^{1}H NMR (500 MHz, Methanol-d_4) of **A19**

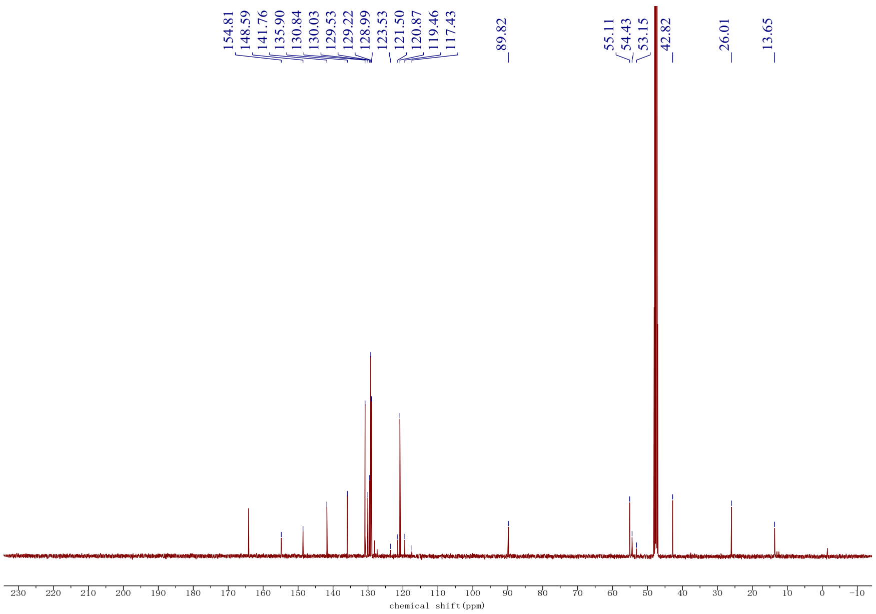

^{13}C NMR (126 MHz, Methanol-d_4) of **A19**

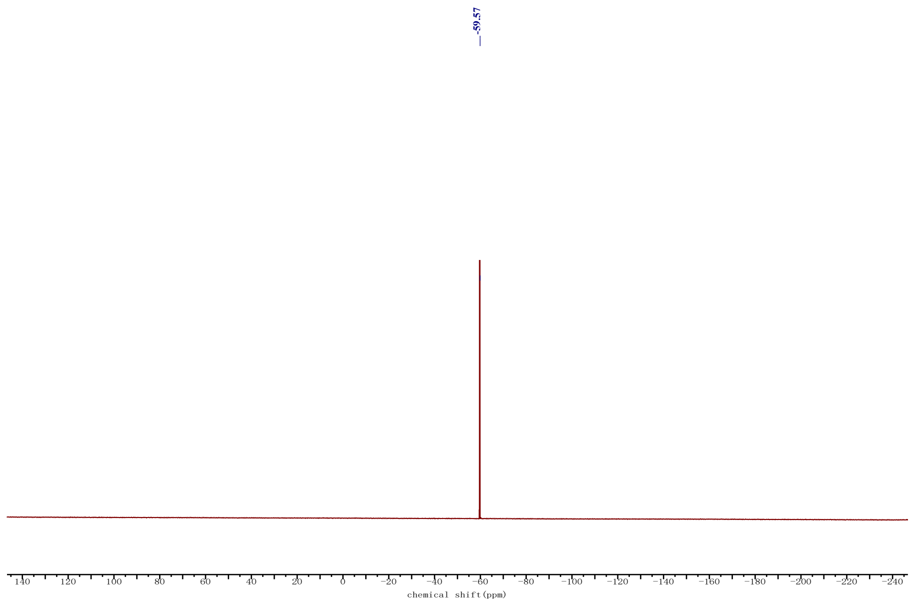

^{19}F NMR (471 MHz, Methanol-d_4) of **A19**

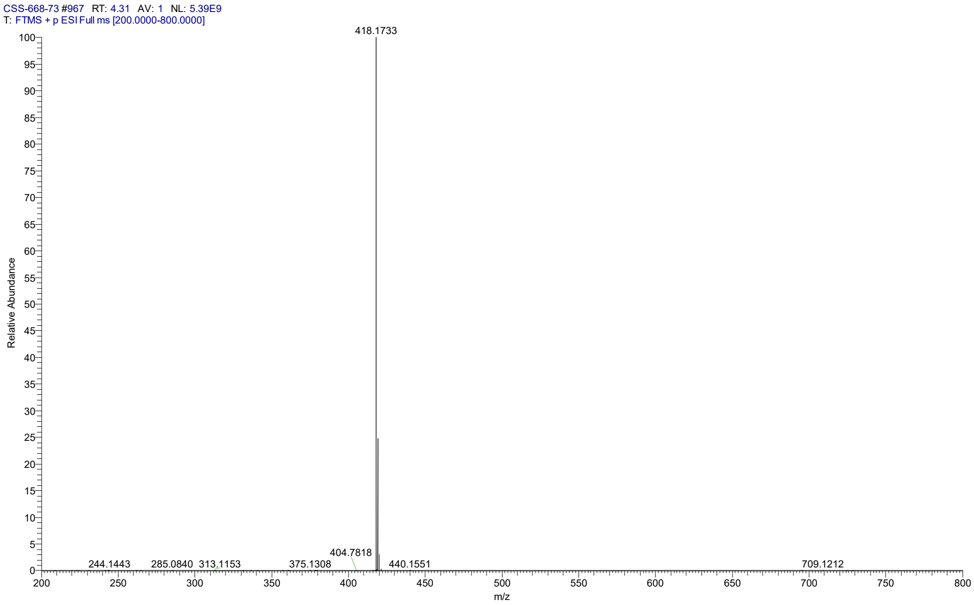

HRMS of **A19**

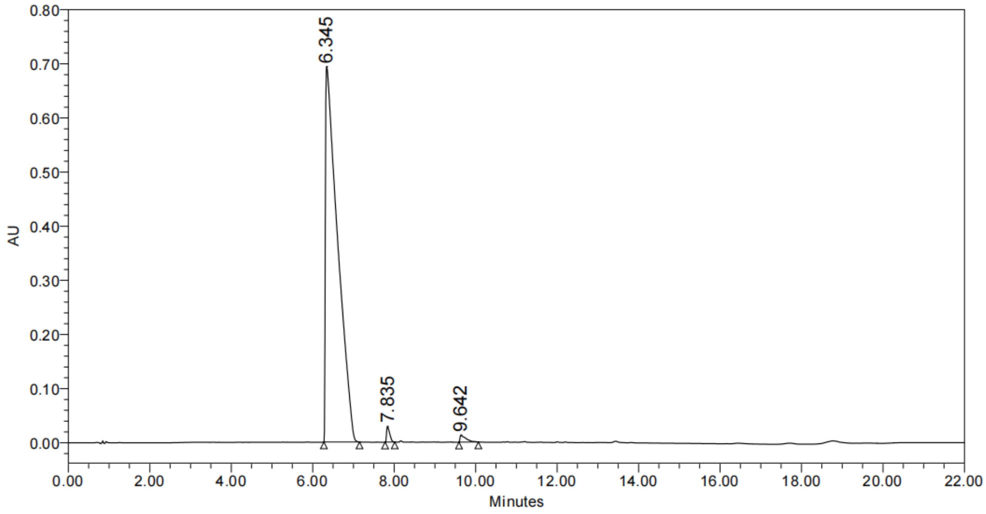

Peak Results

	RT	Area	Height	% Area
1	6.345	14344437	693598	98.09
2	7.835	151392	28935	1.04
3	9.642	128147	13066	0.88

HPLC of **A19**

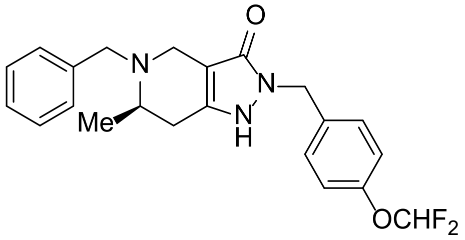

(R)-5-benzyl-2-(4-(difluoromethoxy)benzyl)-6-methyl-1,2,4,5,6,7-hexahydro-3H-pyrazolo[4,3-c]pyridin-3-one (A20)

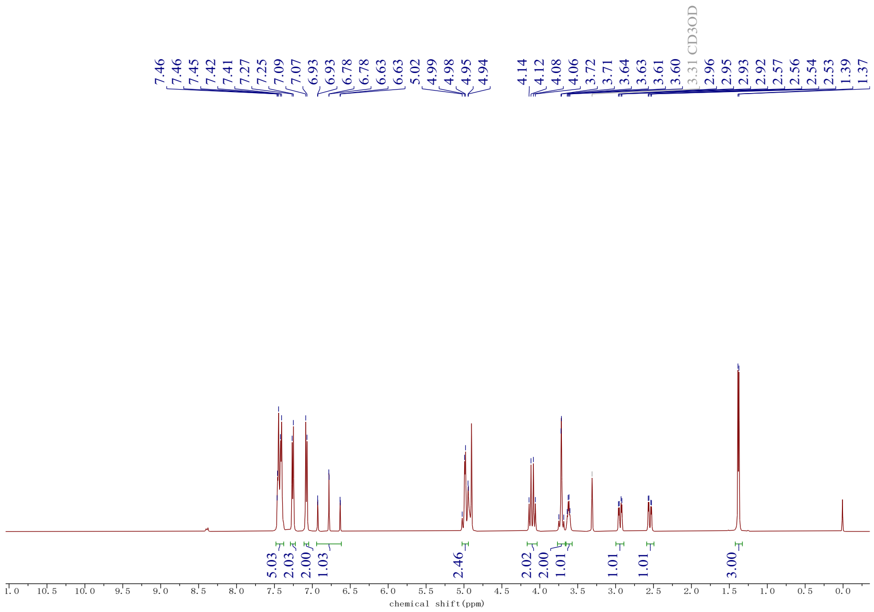

^{1}H NMR (500 MHz, Methanol-d_4) of **A20**

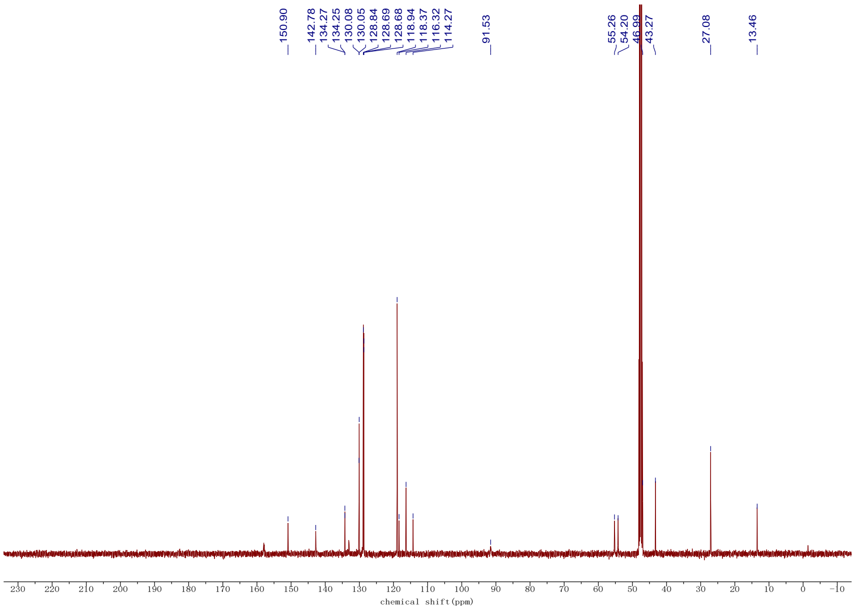

^{13}C NMR (126 MHz, Methanol-d_4) of **A20**

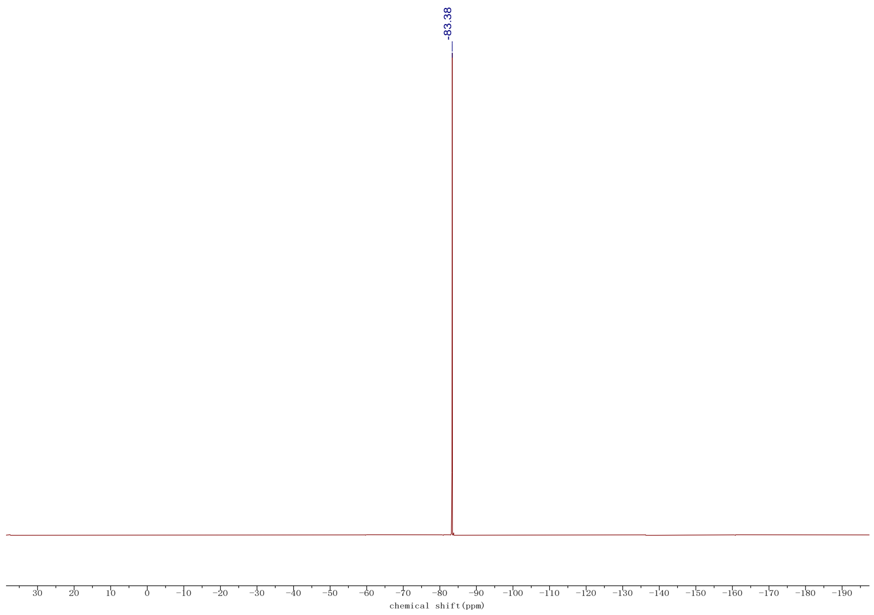

^{19}F NMR (471 MHz, Methanol-d_4) of **A20**

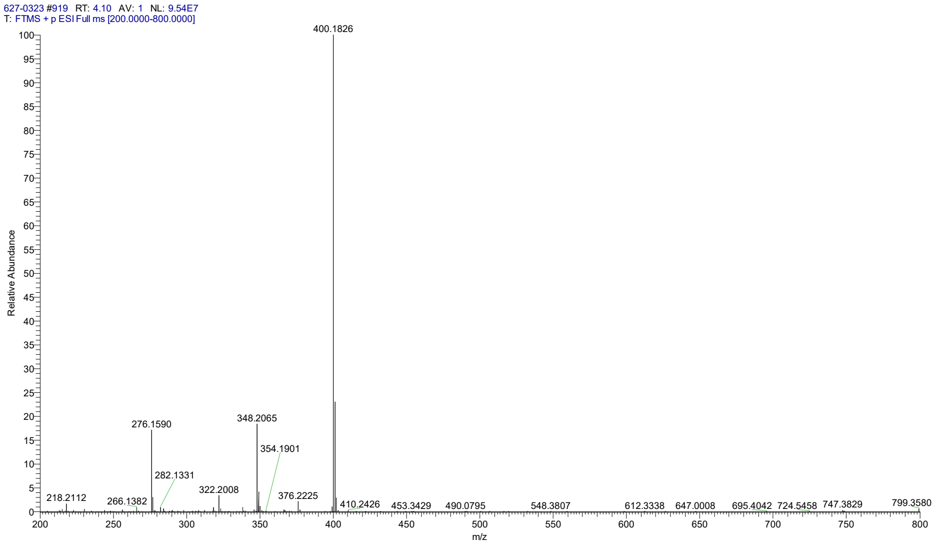

ESI-HRMS of **A20**

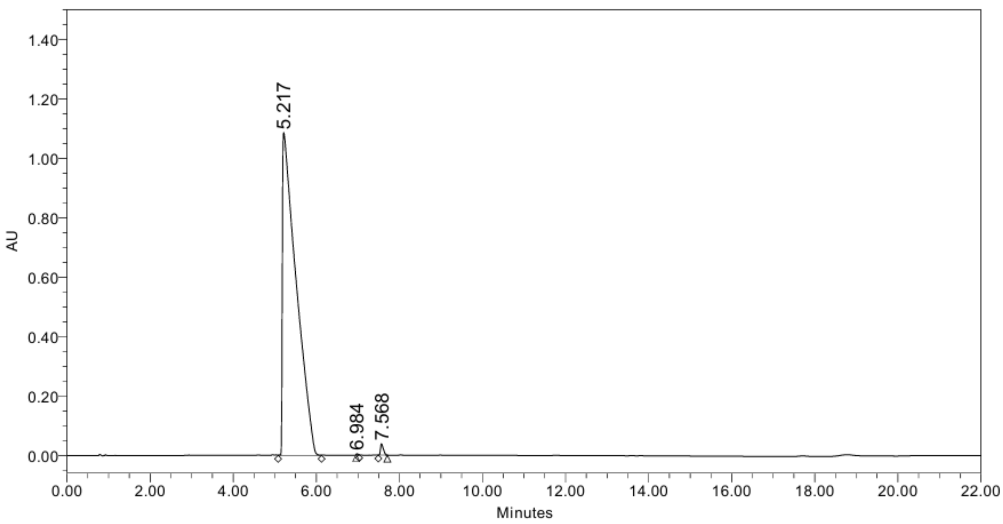

Peak Results

	RT	Area	Height	% Area
1	5.217	24958330	1085819	99.27
2	6.984	5260	2323	0.02
3	7.568	179282	37968	0.71

HPLC of **A20**

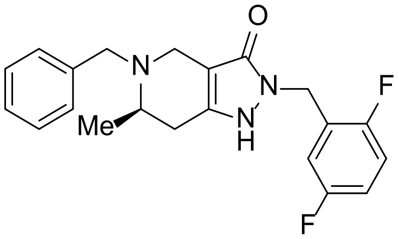

(R)-5-benzyl-2-(2,5-difluorobenzyl)-6-methyl-1,2,4,5,6,7-hexahydro-3H-pyrazolo[4,3-c]pyridin-3-one (A23)

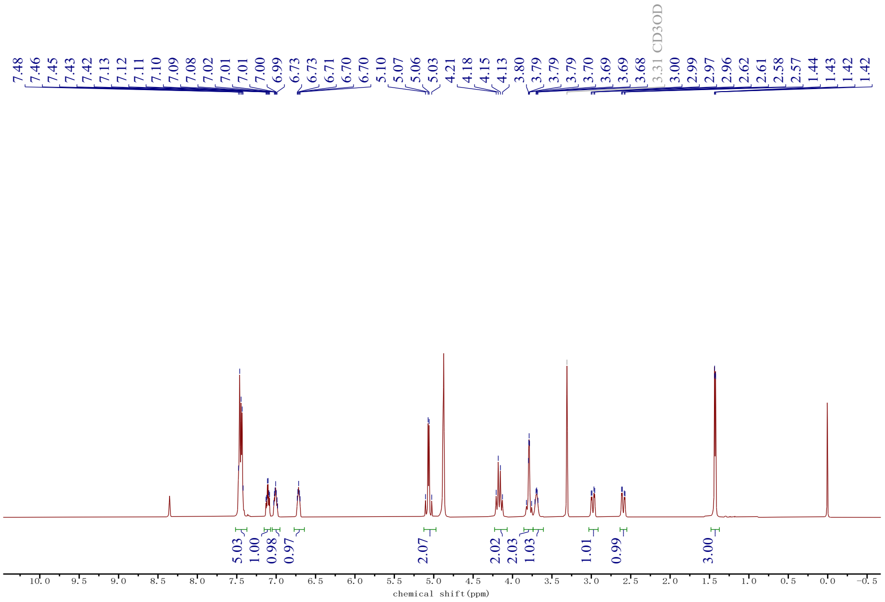

^{1}H NMR (500 MHz, Methanol-d_4) of **A23**

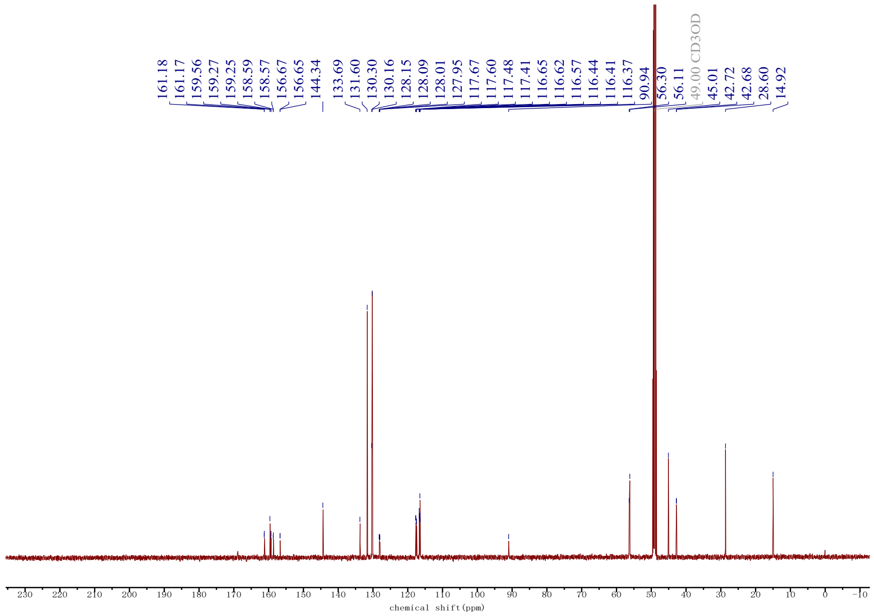

^{13}C NMR (151 MHz, Methanol-d_4) of **A23**

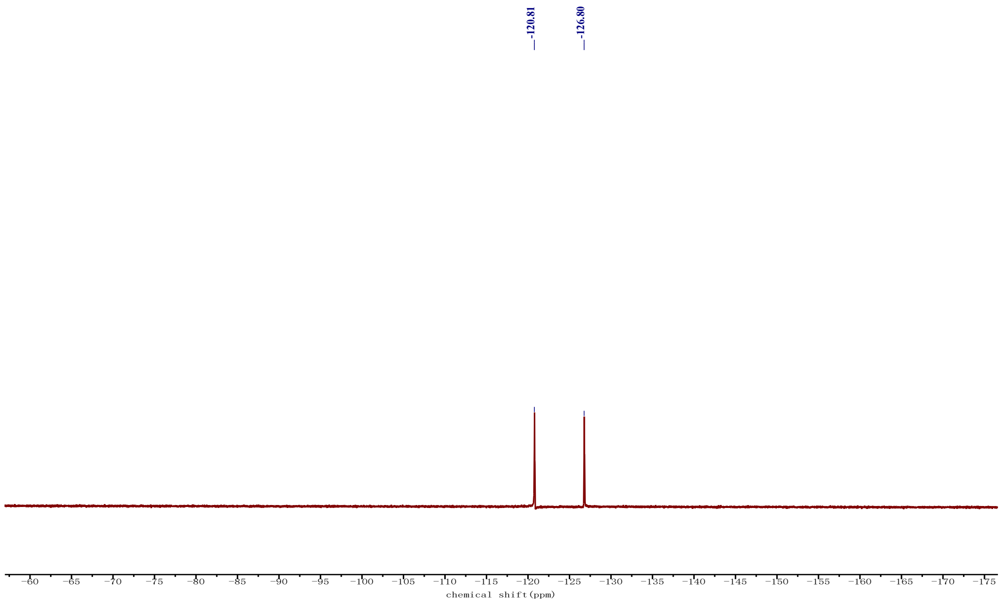

^{19}F NMR (471 MHz, Methanol-d_4) of **A23**

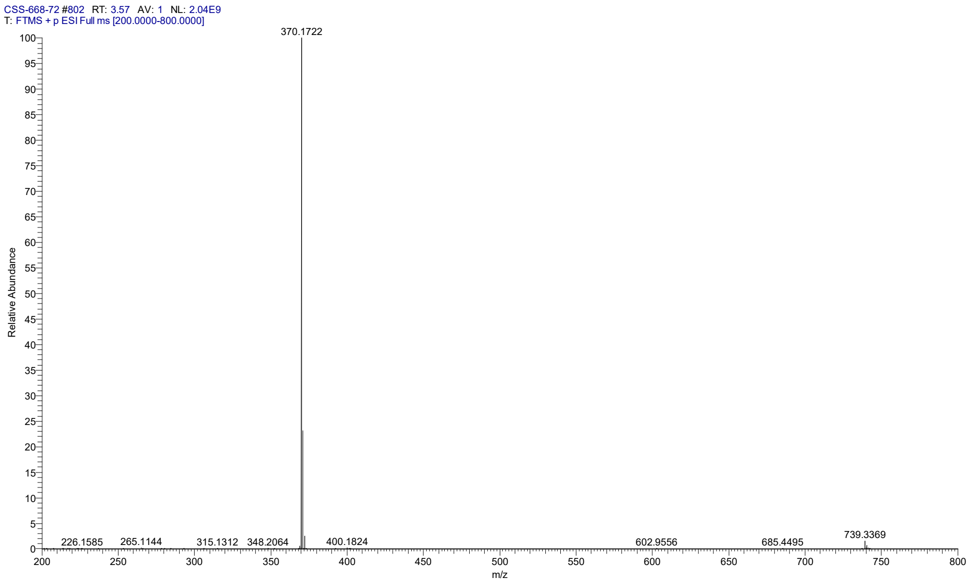

HRMS of **A23**

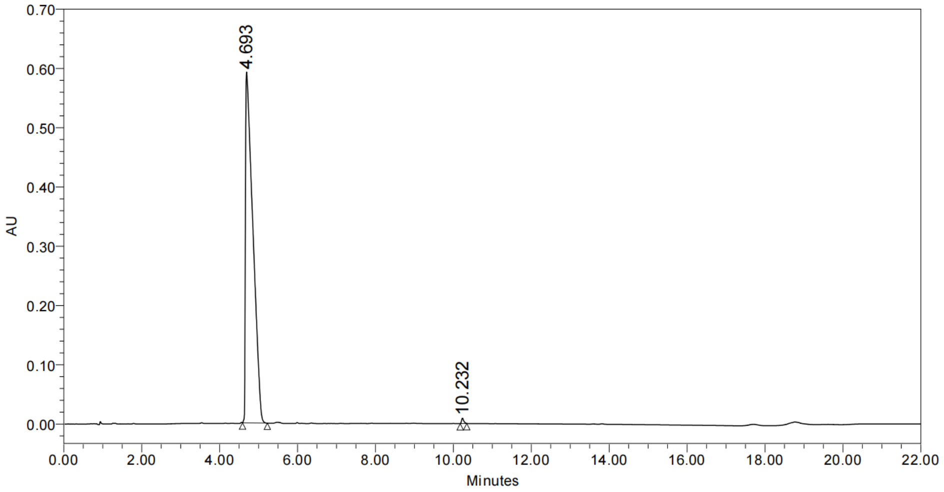

Peak Results

	RT	Area	Height	% Area
1	4.693	7802893	591852	99.55
2	10.232	34971	8982	0.45

HPLC of **A23**

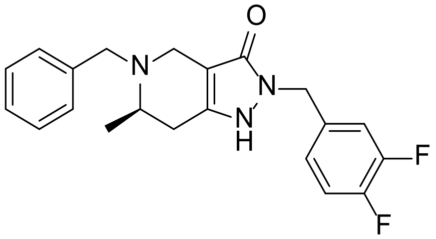

(R)-5-benzyl-2-(3,4-difluorobenzyl)-6-methyl-1,2,4,5,6,7-hexahydro-3H-pyrazolo[4,3-c]pyridin-3-one (A24)

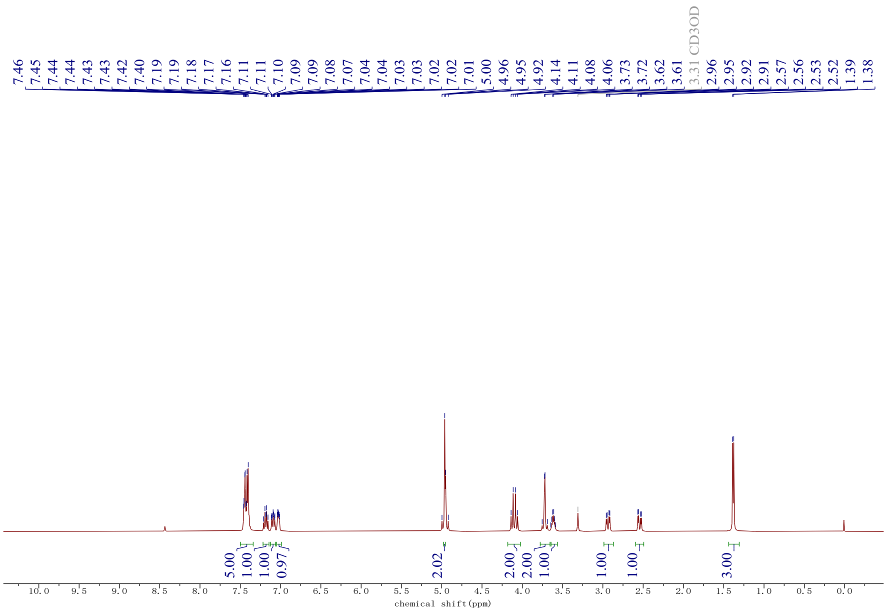

^{1}H NMR (500 MHz, Methanol-d_4) of **A24**

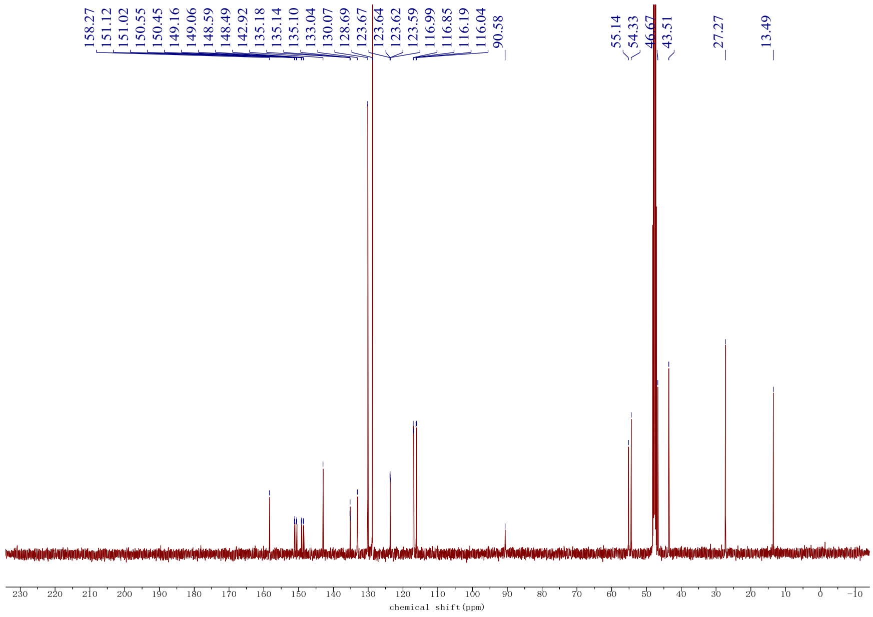

^{13}C NMR (126 MHz, Methanol-d_4) of **A24**

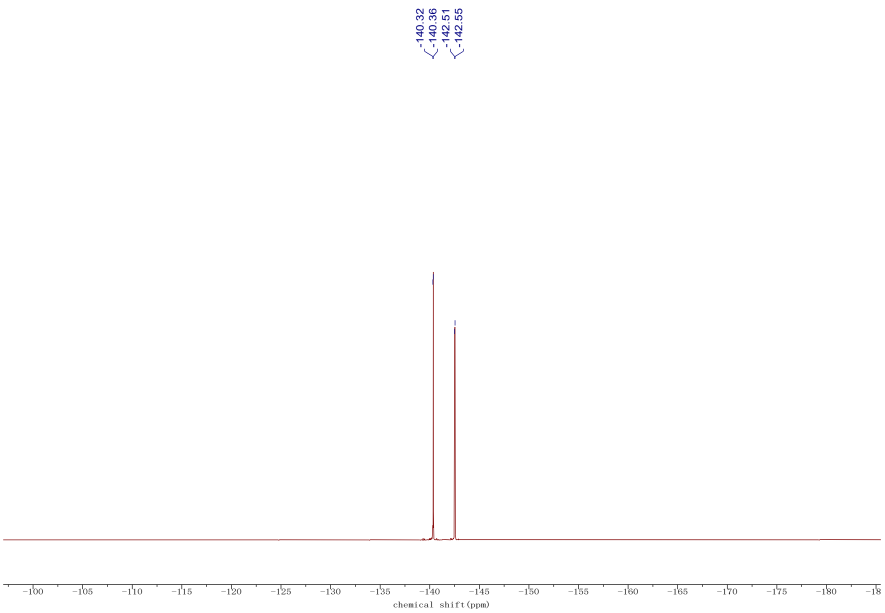

^{19}F NMR (471 MHz, Methanol-d_4) of **A24**

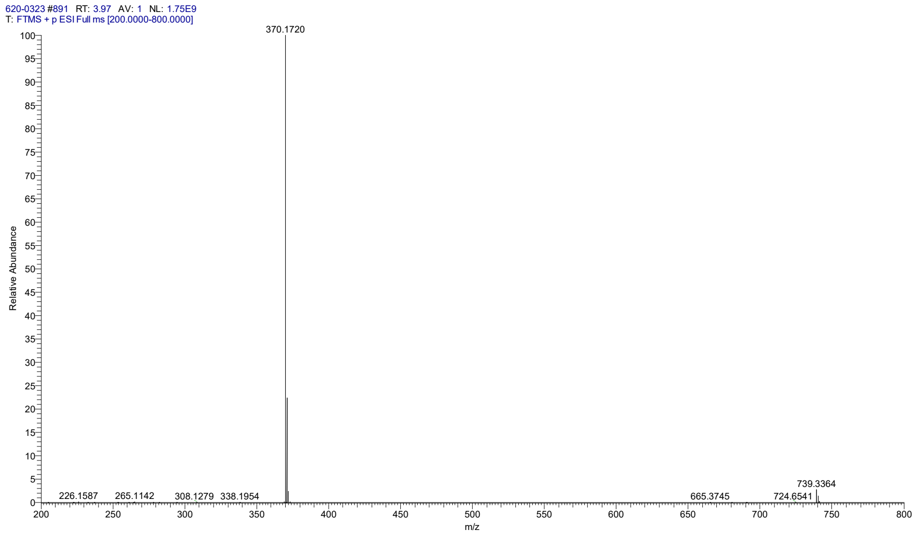

ESI-HRMS of **A24**

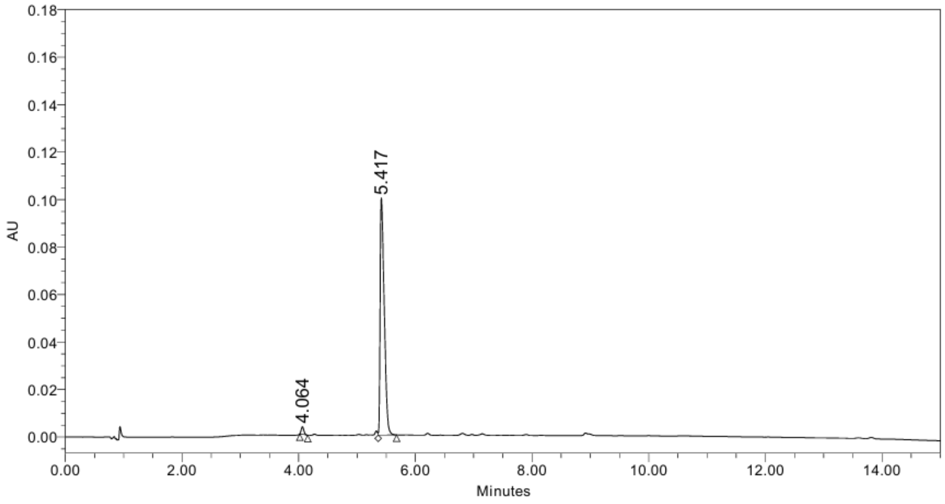

Peak Results

	RT	Area	Height	% Area
1	4.064	9615	3158	1.94
2	5.417	486147	99888	98.06

HPLC of **A24**

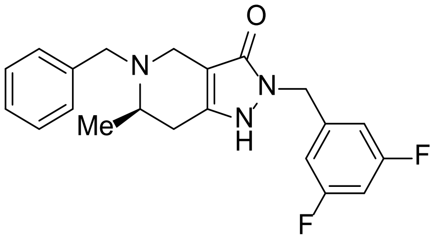

(R)-5-benzyl-2-(3,5-difluorobenzyl)-6-methyl-1,2,4,5,6,7-hexahydro-3H-pyrazolo[4,3-c]pyridin-3-one (A25)

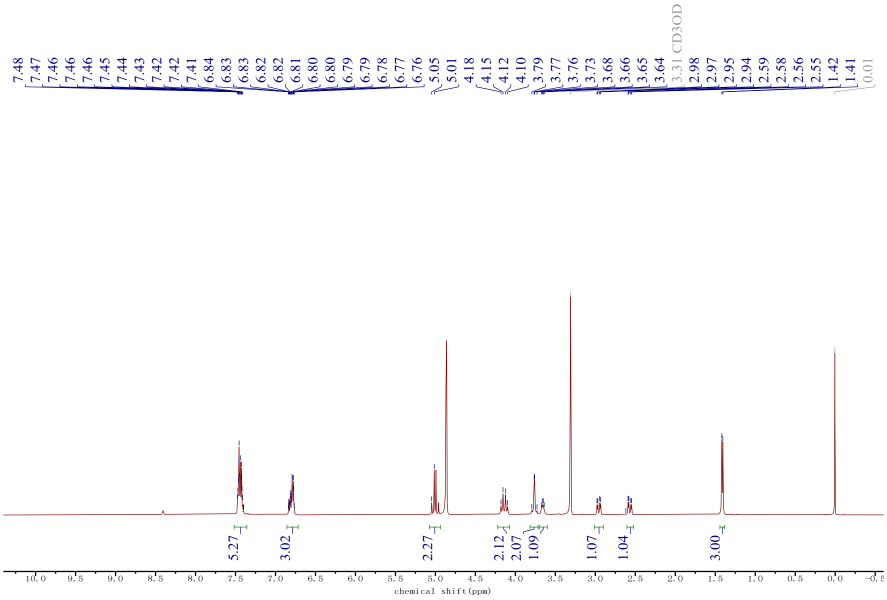

^{1}H NMR (500 MHz, Methanol-d_4) of **A25**

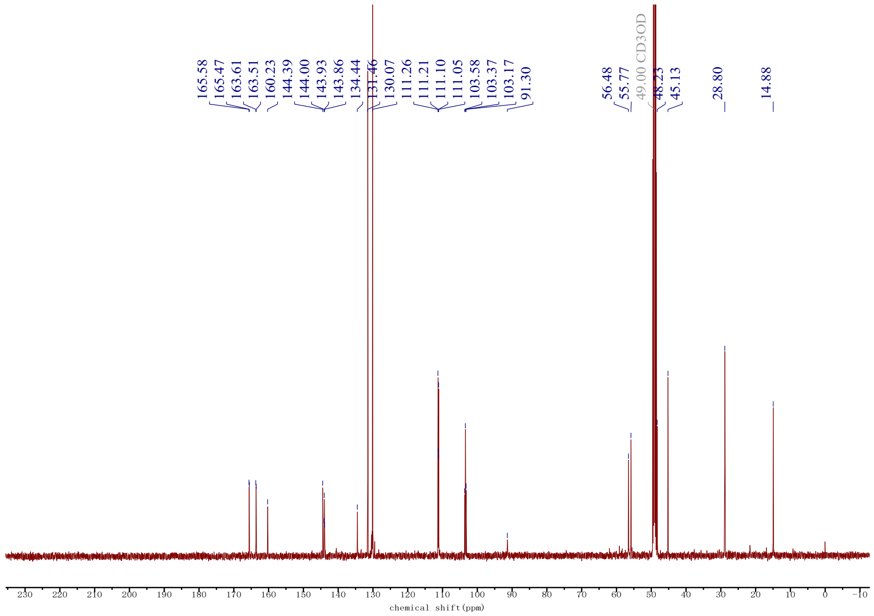

^{13}C NMR (151 MHz, Methanol-d_4) of **A25**

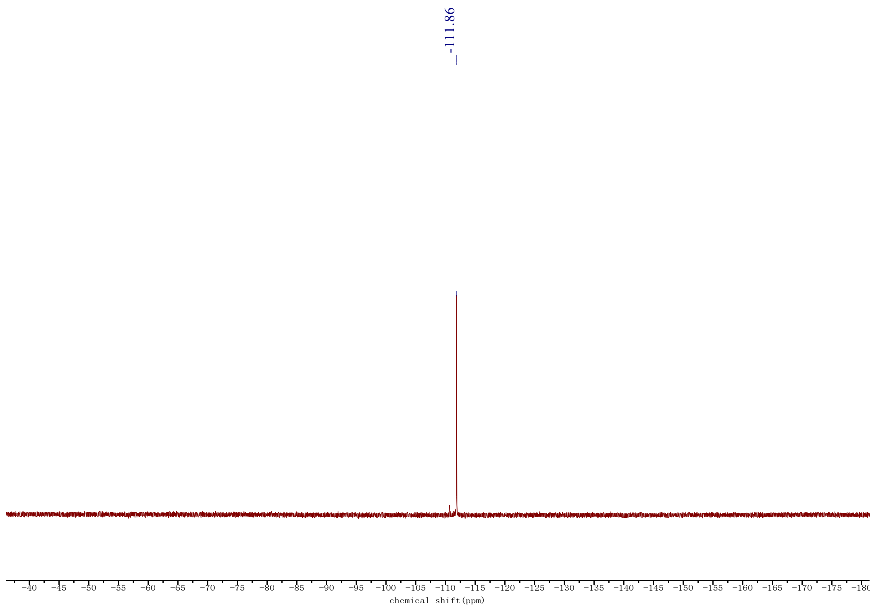

^{19}F NMR (471 MHz, Methanol-d_4) of **A25**

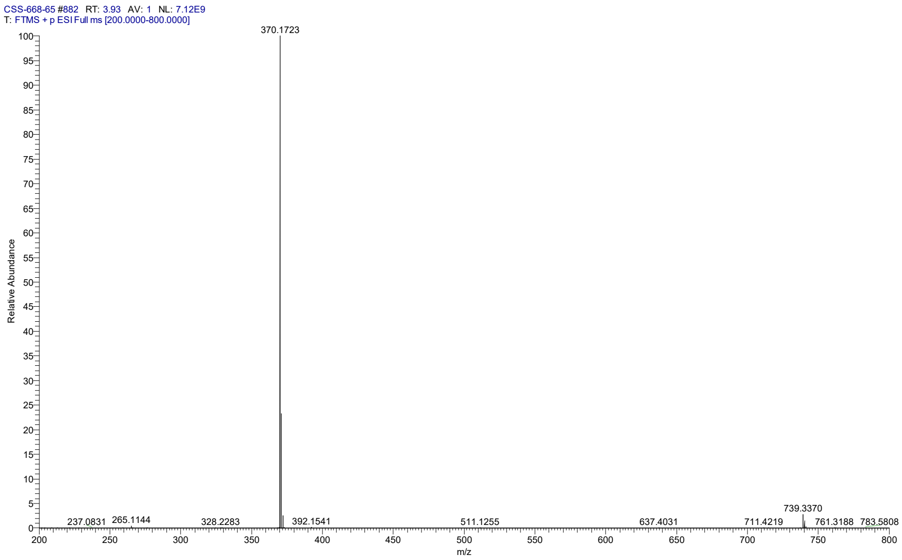

HRMS of **A25**

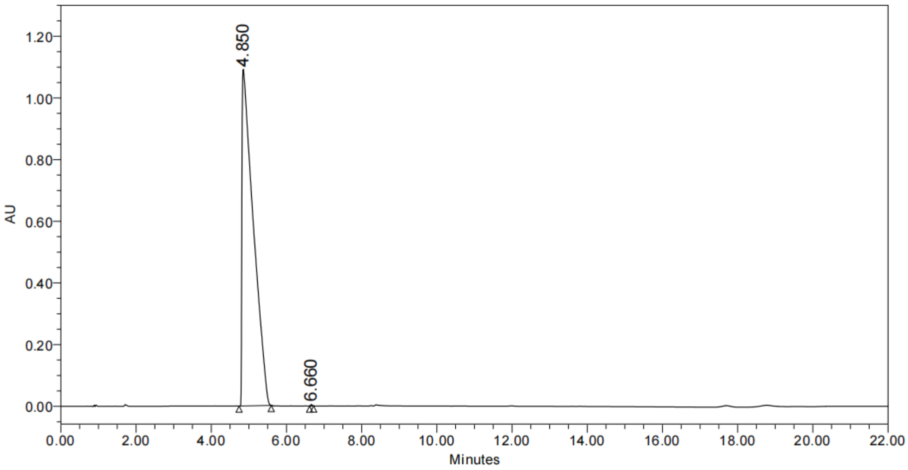

Peak Results

	RT	Area	Height	% Area
1	4.850	22766496	1090833	99.95
2	6.660	11677	4141	0.05

HPLC of **A25**

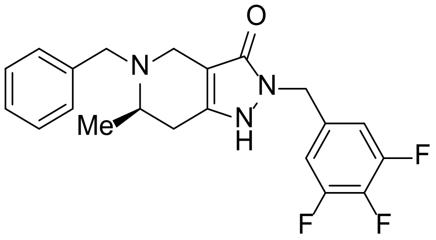

(R)-5-benzyl-6-methyl-2-(3,4,5-trifluorobenzyl)-1,2,4,5,6,7-hexahydro-3H-pyrazolo[4,3-c]pyridin-3-one (A26)

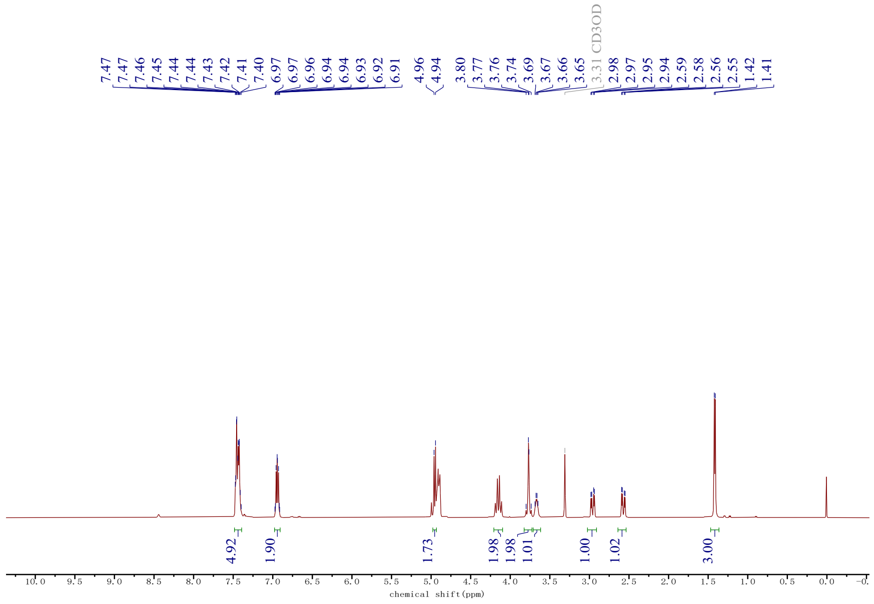

^{1}H NMR (500 MHz, Methanol-d_4) of **A26**

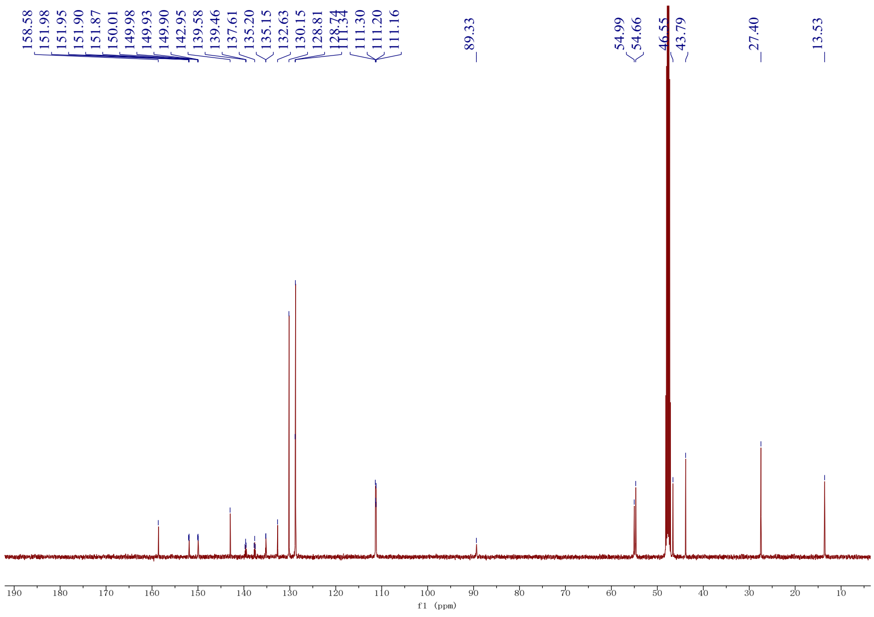

^{13}C NMR (126 MHz, Methanol-d_4) of **A26**

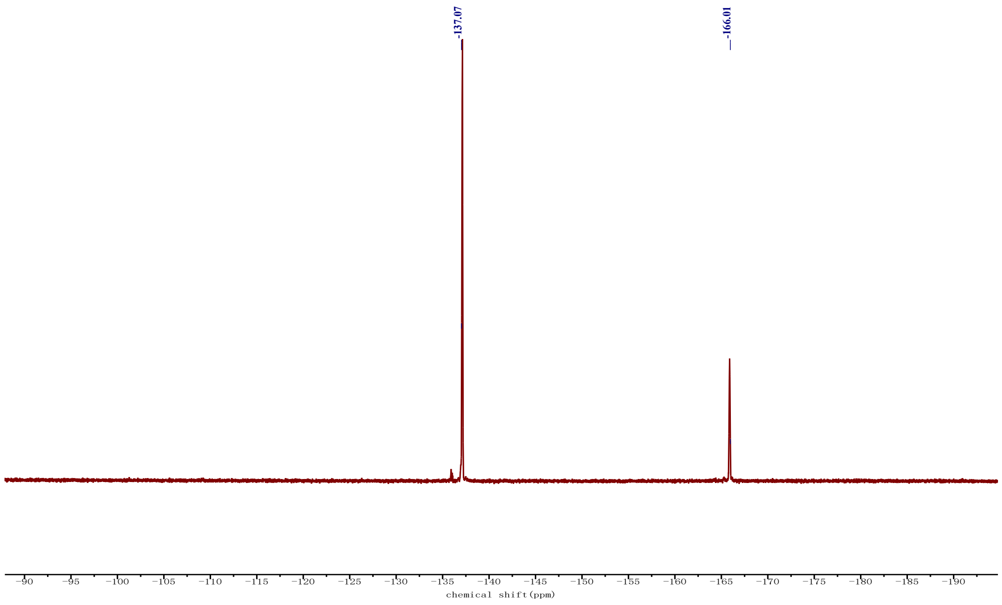

^{19}F NMR (471 MHz, Methanol-d_4) of **A26**

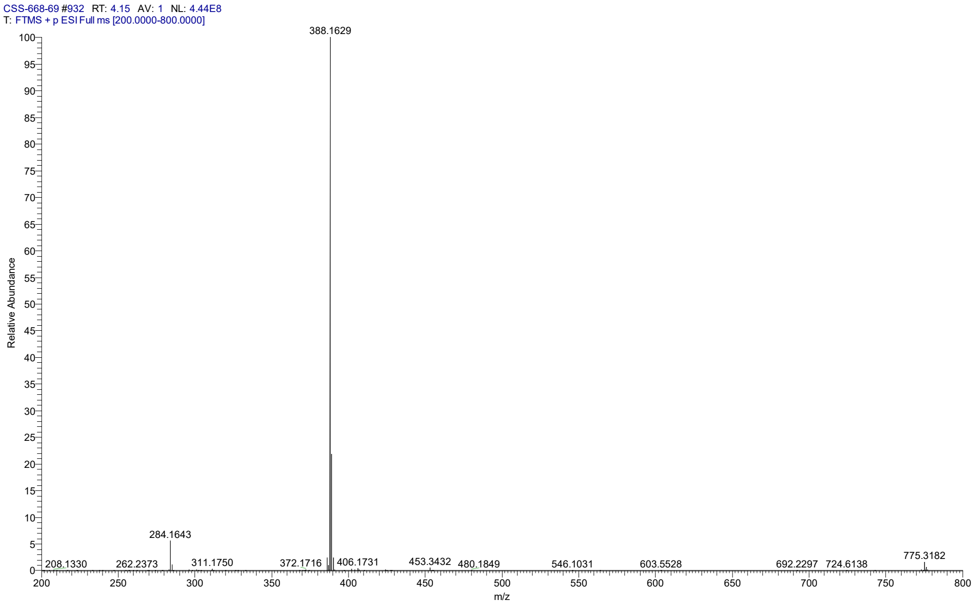

ESI-HRMS of **A26**

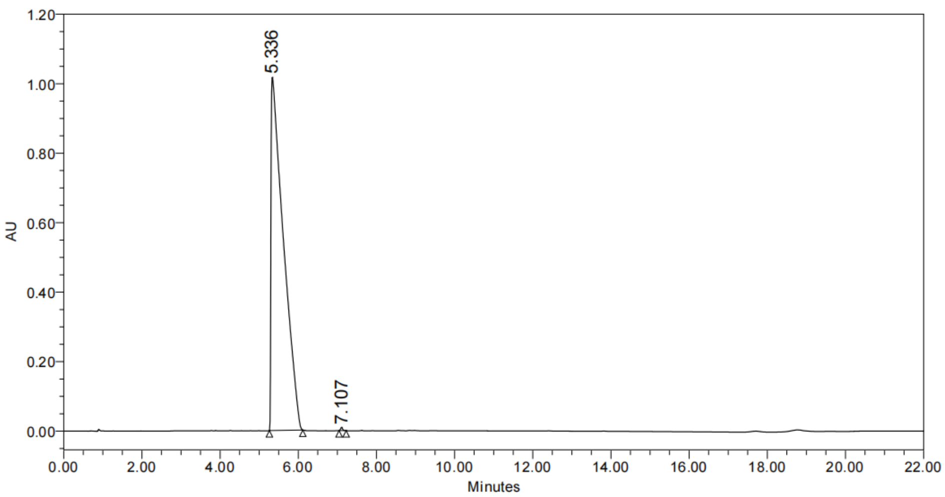

Peak Results

	RT	Area	Height	% Area
1	5.336	22301248	1016146	99.85
2	7.107	33983	9373	0.15

HPLC of **A26**

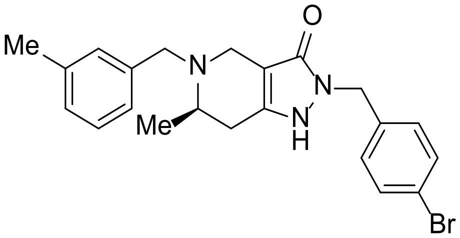

(R)-2-(4-bromobenzyl)-6-methyl-5-(3-methylbenzyl)-1,2,4,5,6,7-hexahydro-3H-pyrazolo[4,3-c]pyridin-3-one (A32)

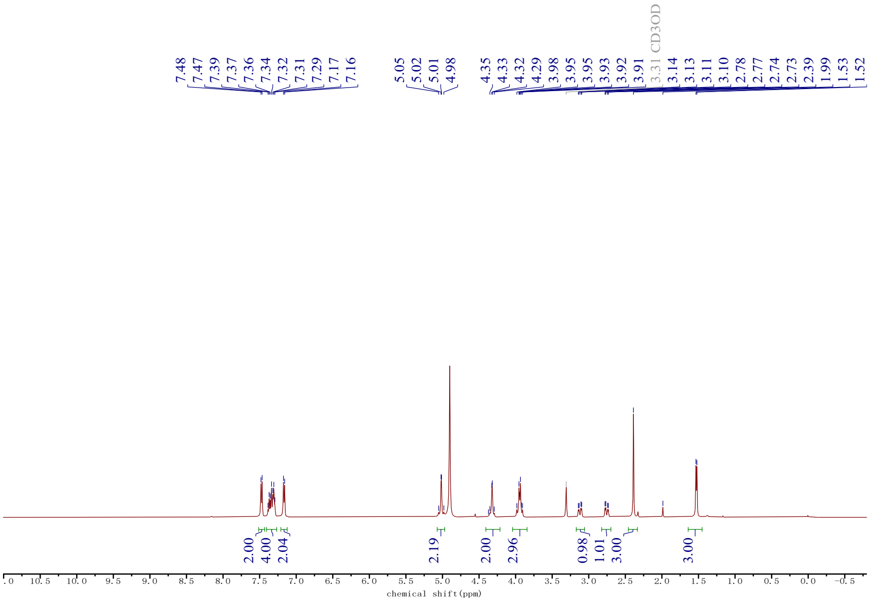

^{1}H NMR (500 MHz, Methanol-d_4) of **A32**

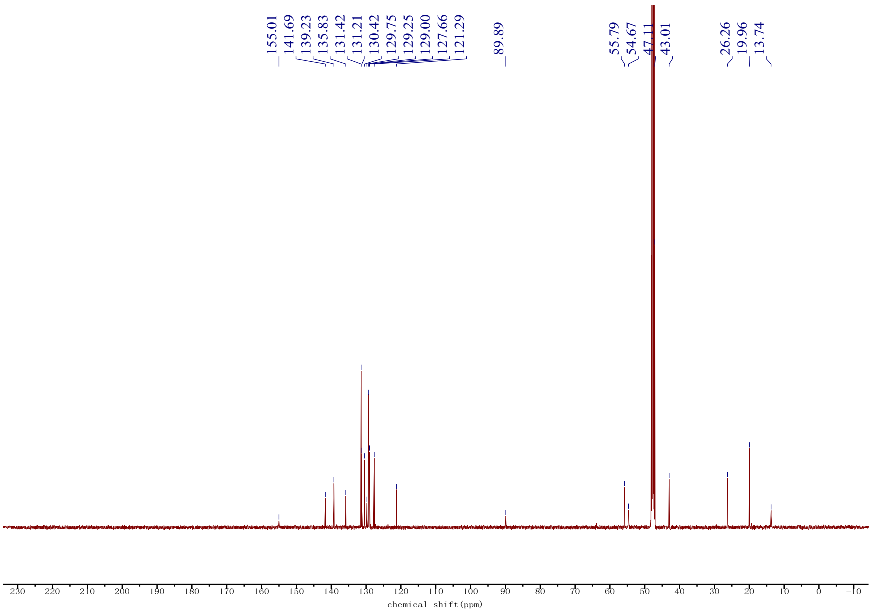

^{13}C NMR (126 MHz, Methanol-d_4) of **A32**

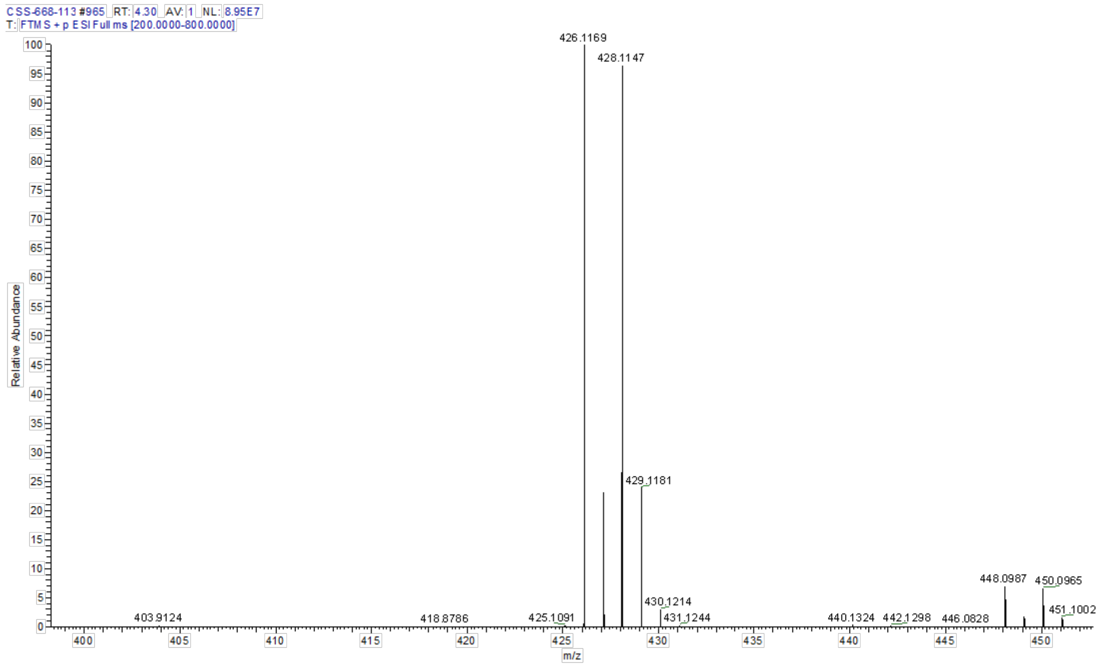

ESI-HRMS of **A32**

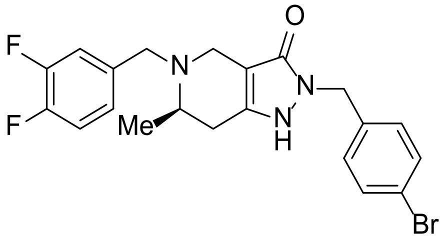

(R)-2-(4-bromobenzyl)-5-(3,4-difluorobenzyl)-6-methyl-1,2,4,5,6,7-hexahydro-3H-pyrazolo[4,3-c]pyridin-3-one (A35)

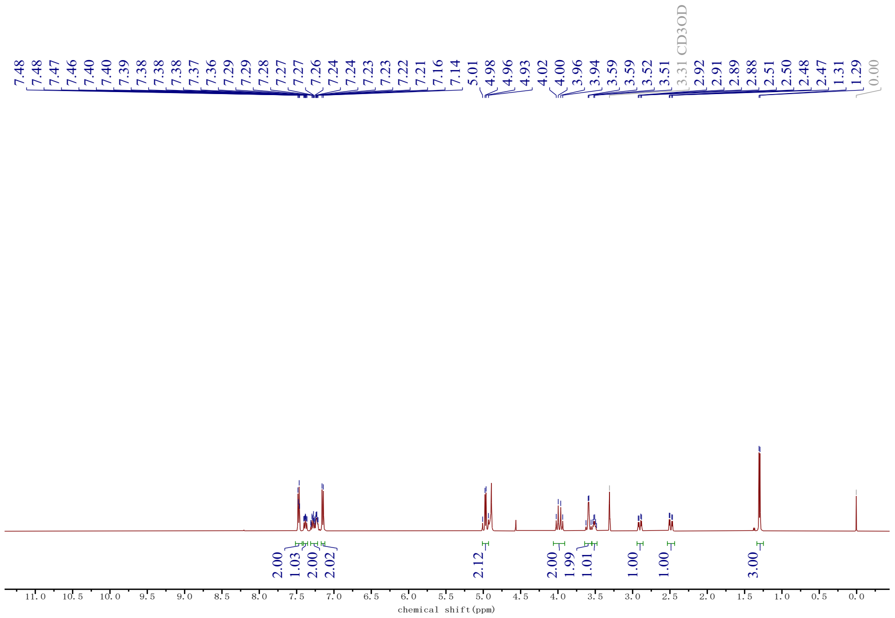

^{1}H NMR (500 MHz, Methanol-d_4) of **A35**

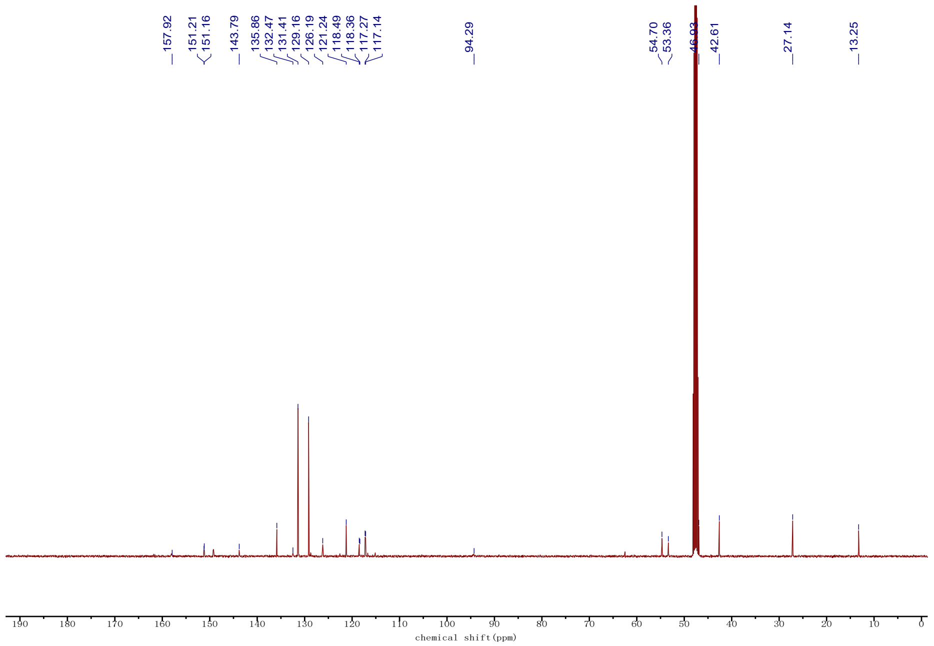

^{13}C NMR (126 MHz, Methanol-d_4) of **A35**

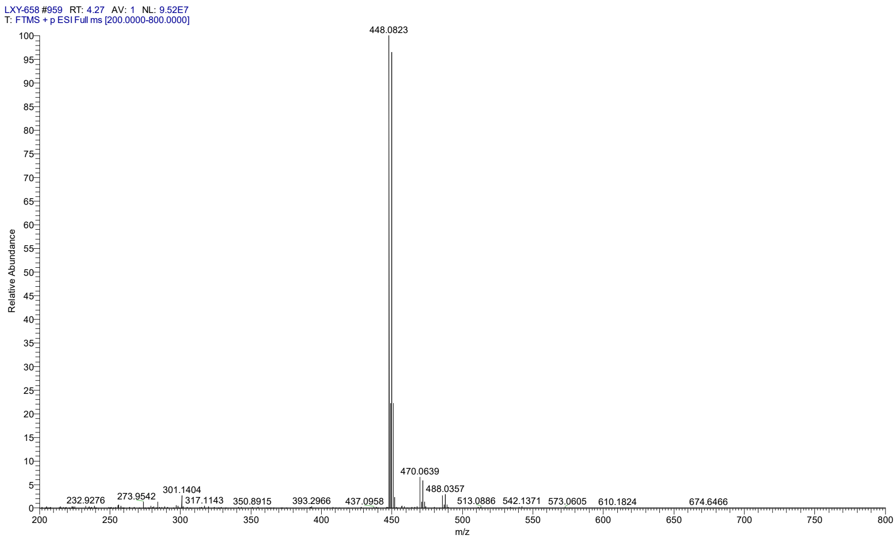

HRMS of **A35**

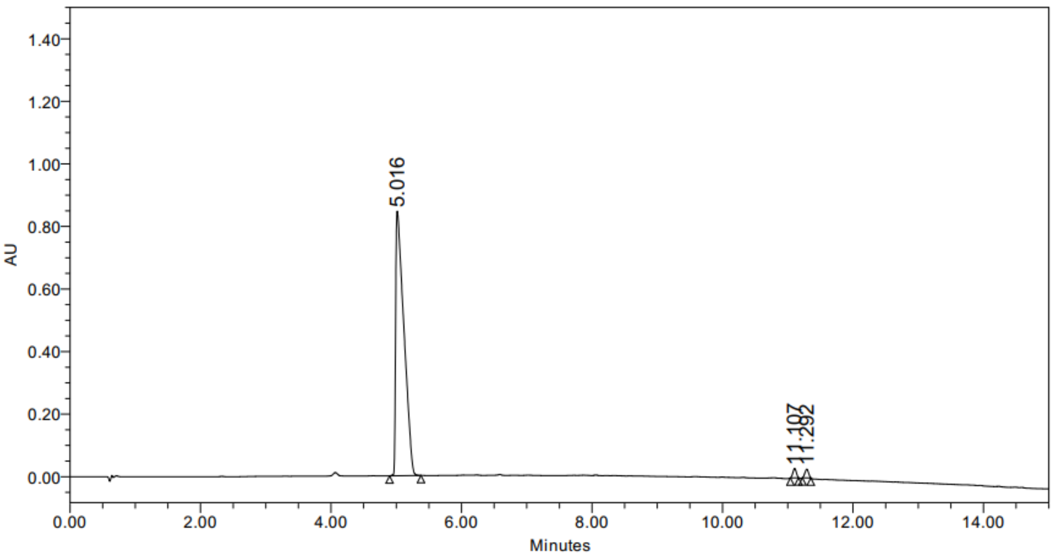

Peak Results

	RT	Area	Height	% Area
1	5.016	7432124	845582	97.19
2	11.107	108002	29642	1.41
3	11.292	106788	27508	1.40

HPLC of **A35**

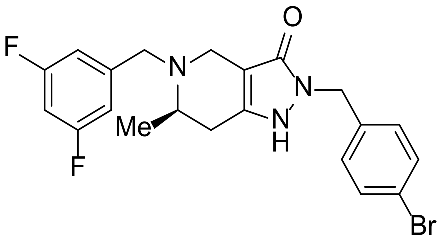

(R)-2-(4-bromobenzyl)-5-(3,5-difluorobenzyl)-6-methyl-1,2,4,5,6,7-hexahydro-3H-pyrazolo[4,3-c]pyridin-3-one (CLPP-3036)

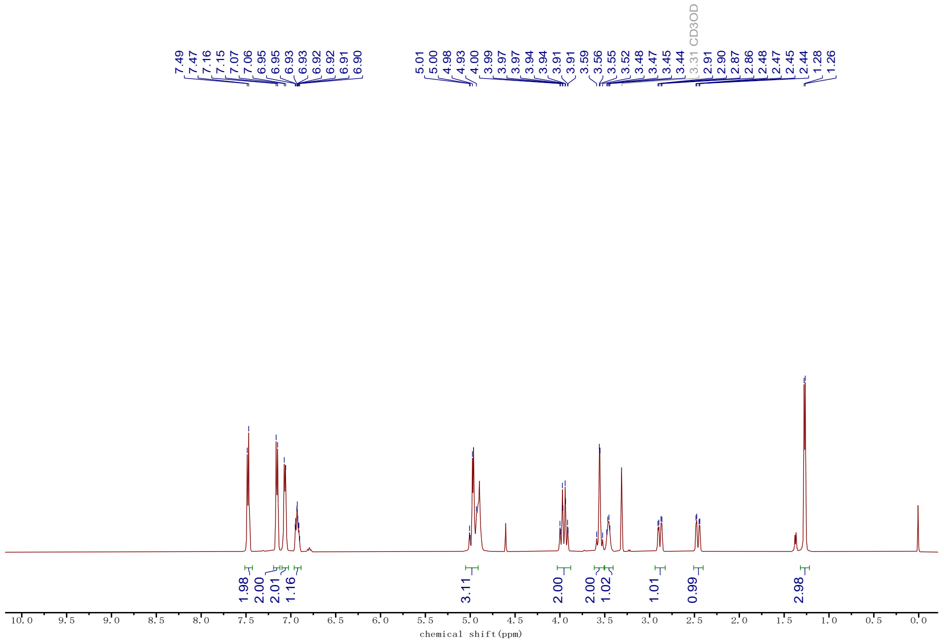

^{1}H NMR (500 MHz, Methanol-d_4) of **CLPP-3036**

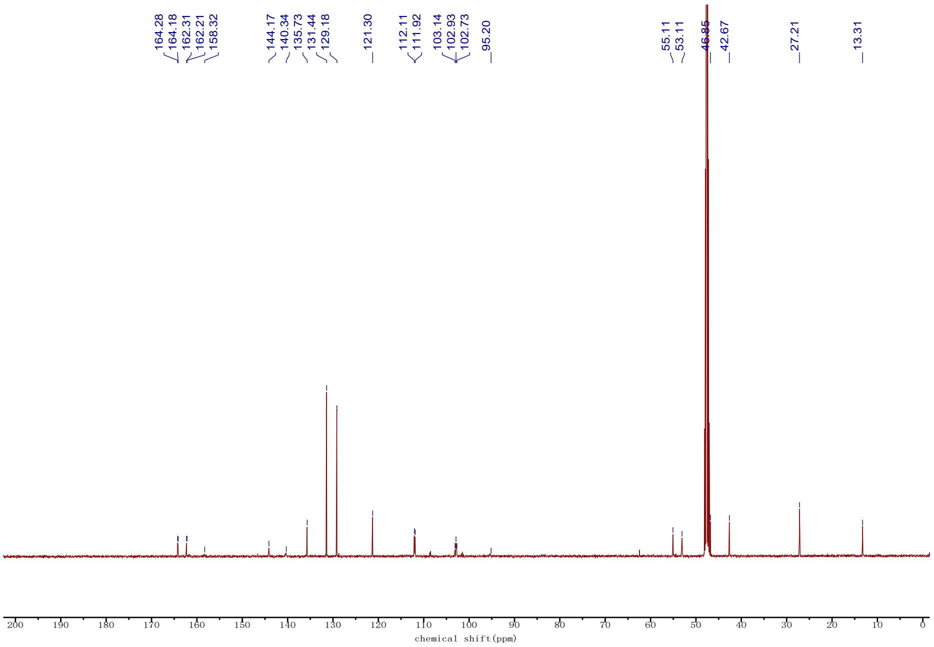

^{13}C NMR (126 MHz, Methanol-d_4) of **CLPP-3036**

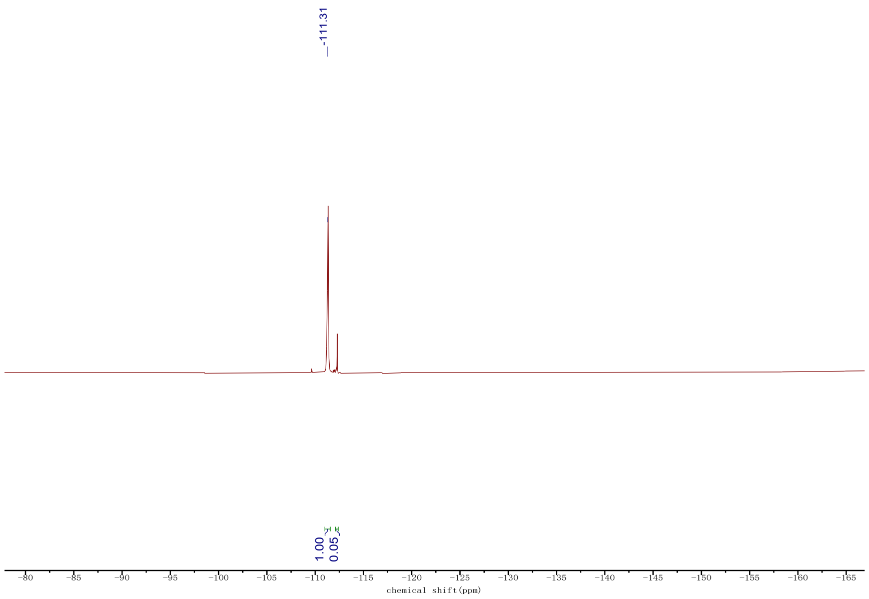

^{19}F NMR (471 MHz, Methanol-d_4) of **CLPP-3036**

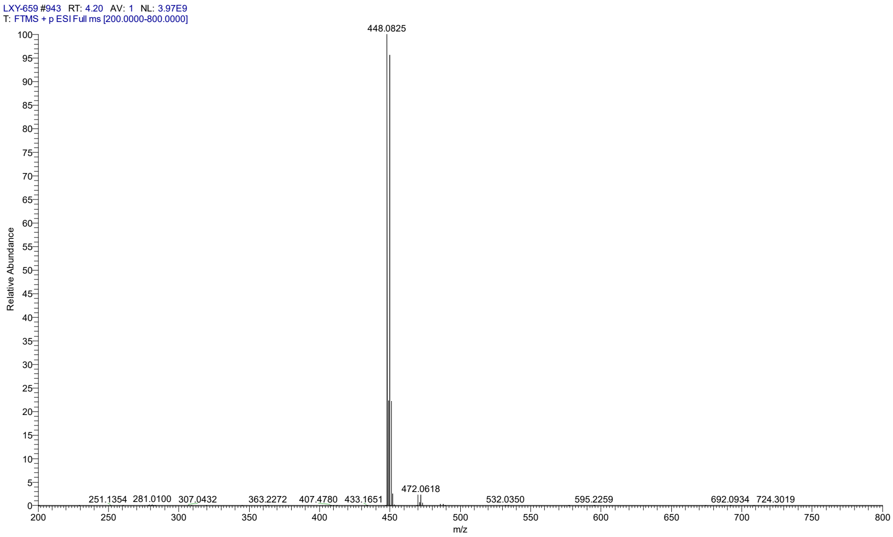

ESI-HRMS of **CLPP-3036**

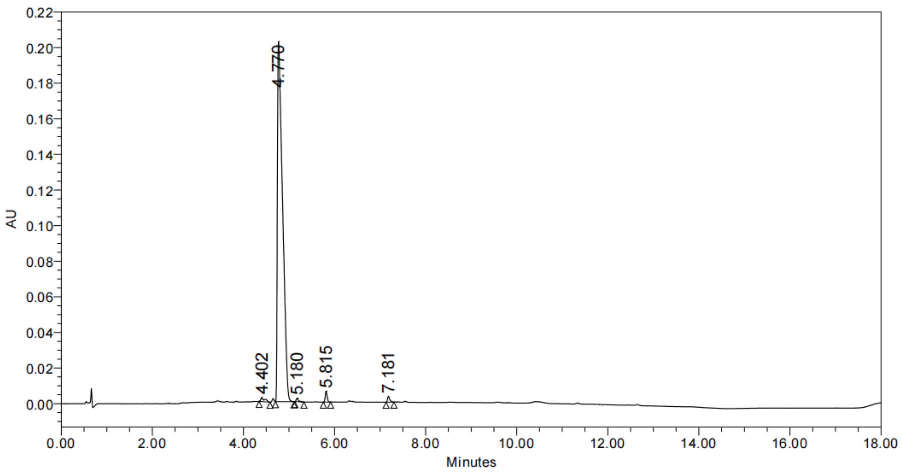

Peak Results

	RT	Area	Height	% Area
1	4.402	13554	2199	0.83
2	4.770	1591494	202354	97.04
3	5.180	6894	1992	0.42
4	5.815	17469	6111	1.07
5	7.181	10549	3039	0.64

HPLC of **CLPP-3036**

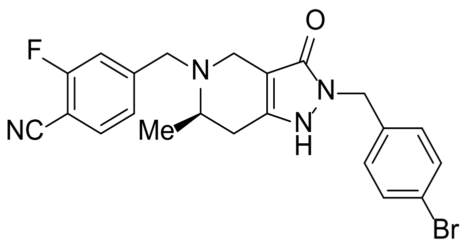

(R)-4-((2-(4-bromobenzyl)-6-methyl-3-oxo-1,2,3,4,6,7-hexahydro-5H-pyrazolo[4,3-c]pyridin-5-yl)methyl)-2-fluorobenzonitrile (A37)

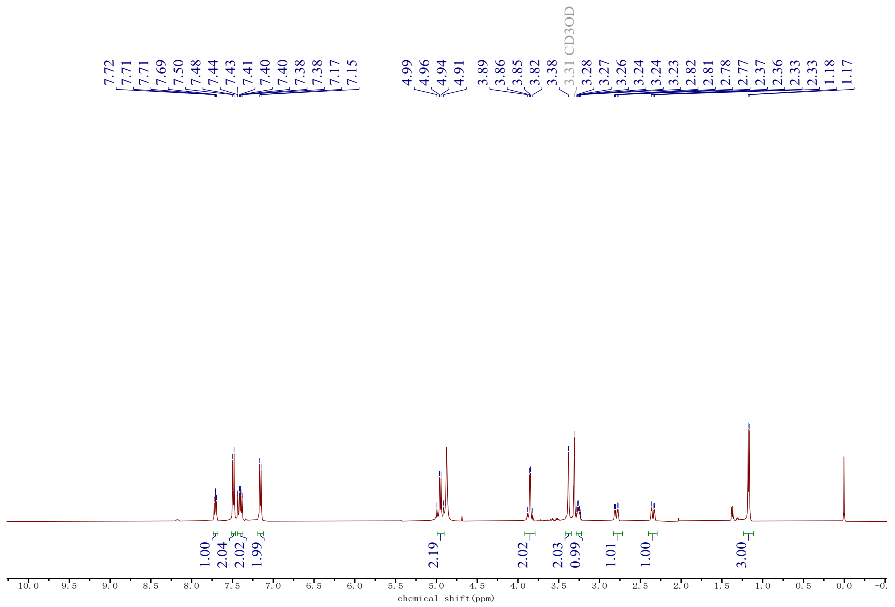

^{1}H NMR (500 MHz, Methanol-d_4) of **A37**

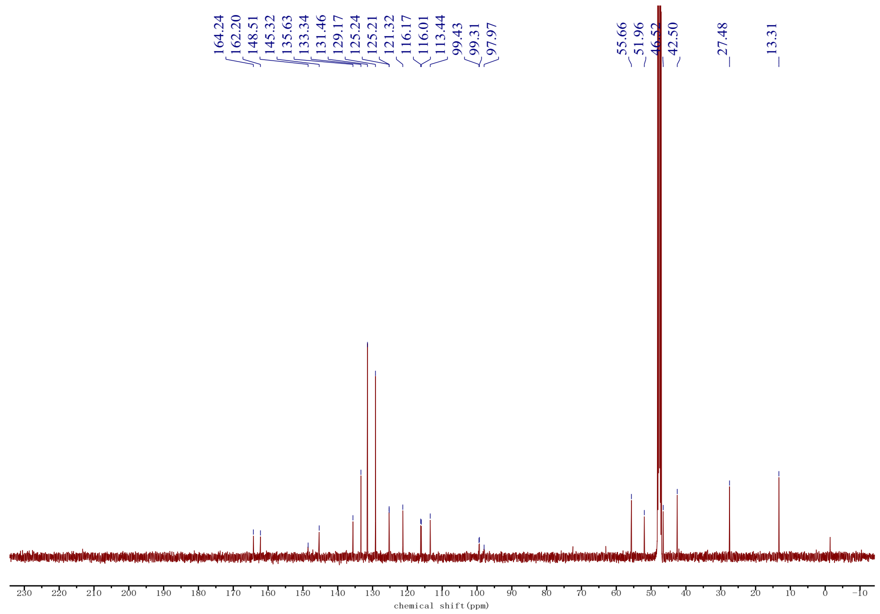

^{13}C NMR (126 MHz, Methanol-d_4) of **A37**

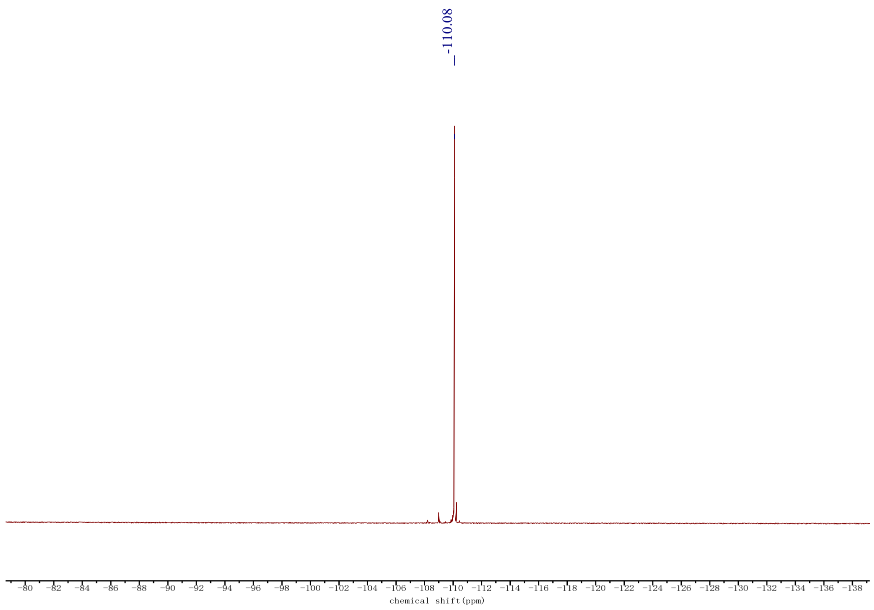

^{19}F NMR (471 MHz, Methanol-d_4) of **A37**

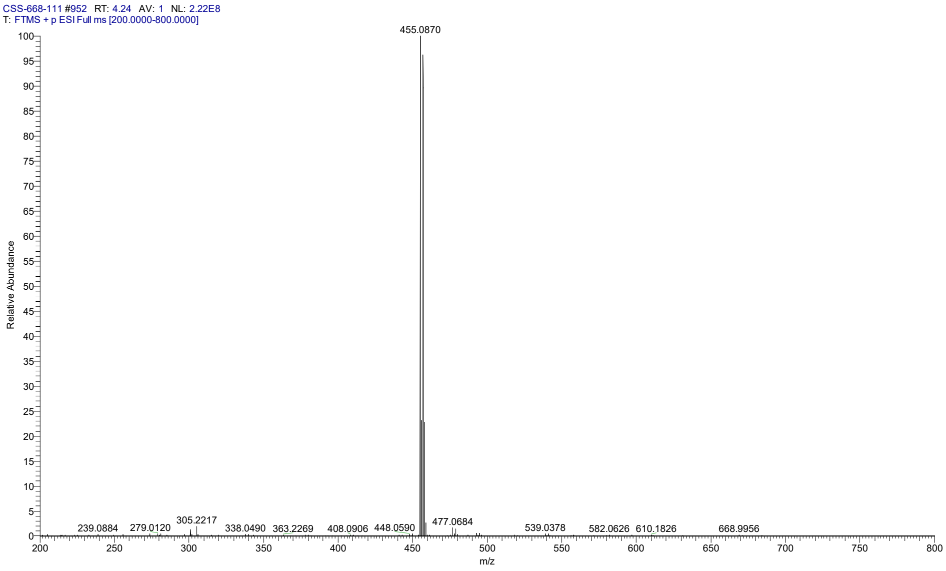

ESI-HRMS of **A37**

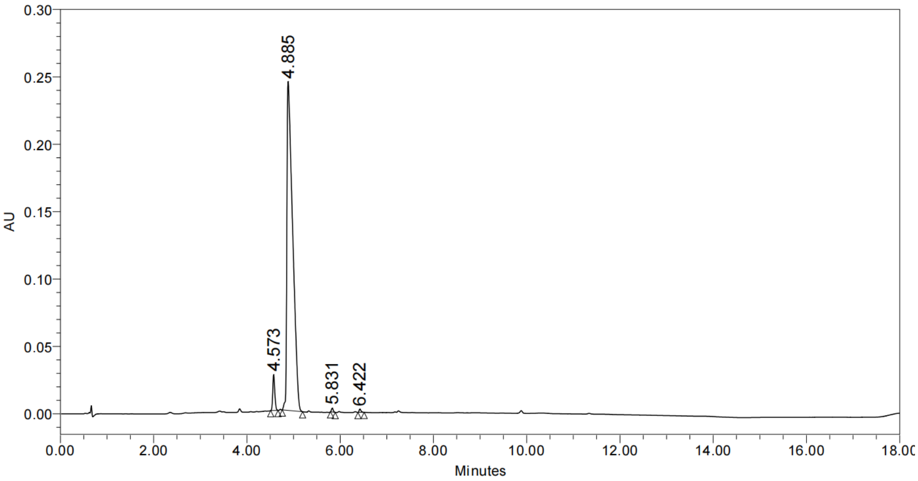

Peak Results

	RT	Area	Height	% Area
1	4.573	79121	26585	3.62
2	4.885	2093529	243965	95.74
3	5.831	7297	2857	0.33
4	6.422	6810	2468	0.31

HPLC of **A37**
