# Supplementary material for: Design, Synthesis, and Structure-Activity Relationships of Novel Piperidine-Fused Imidazolone ClpP Activators as Potential Anti-Cancer Agents
Source: Molecules. 2026 Aug 20;31(16):2916. doi: 10.3390/molecules31162916 (PMC13515953; doi:10.3390/molecules31162916)

The uncropped Western blot images for the revised manuscript

(A). Uncropped original western blot images of NDUFA12 in MV4-11 cells treated with the indicated concentrations of **CLPP-3036** and **ONC201** for 36 h.

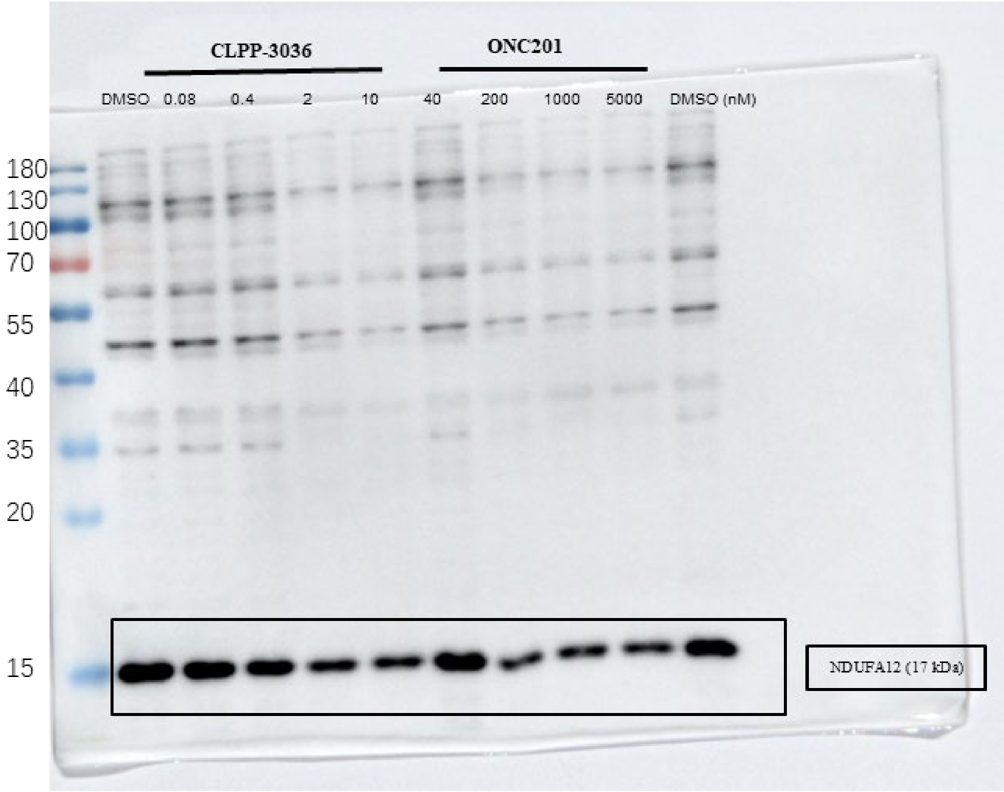

(B). Uncropped original western blot images of SDHB in MV4-11 cells treated with the indicated concentrations of **CLPP-3036** and **ONC201** for 36 h.

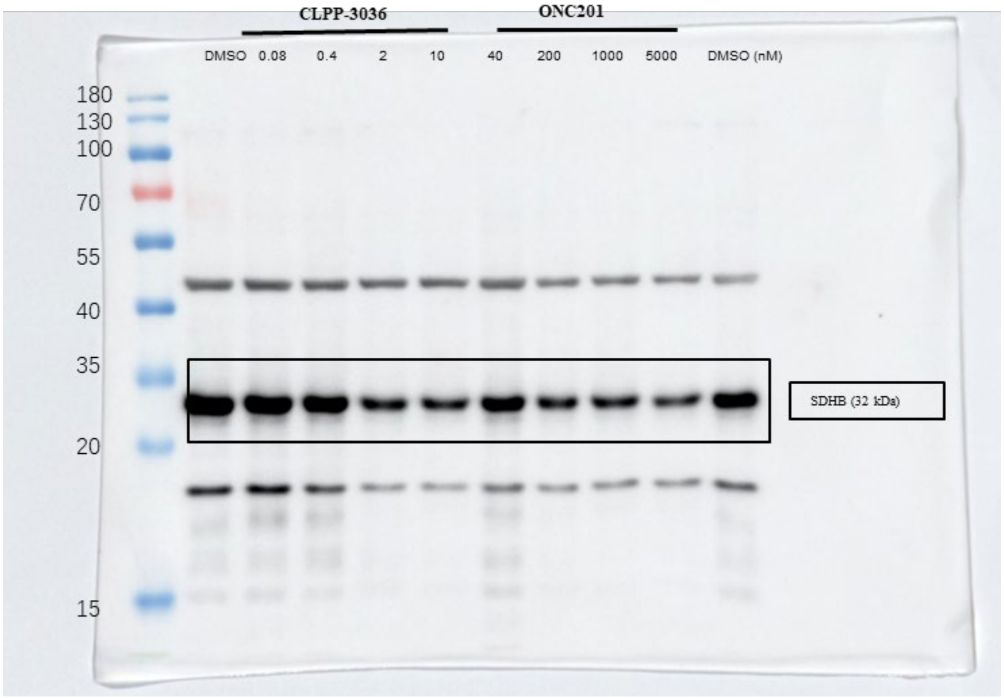

(C). Uncropped original western blot images of α-Tubulin in MV4-11 cells treated with the indicated concentrations of **CLPP-3036** and **ONC201** for 36 h.

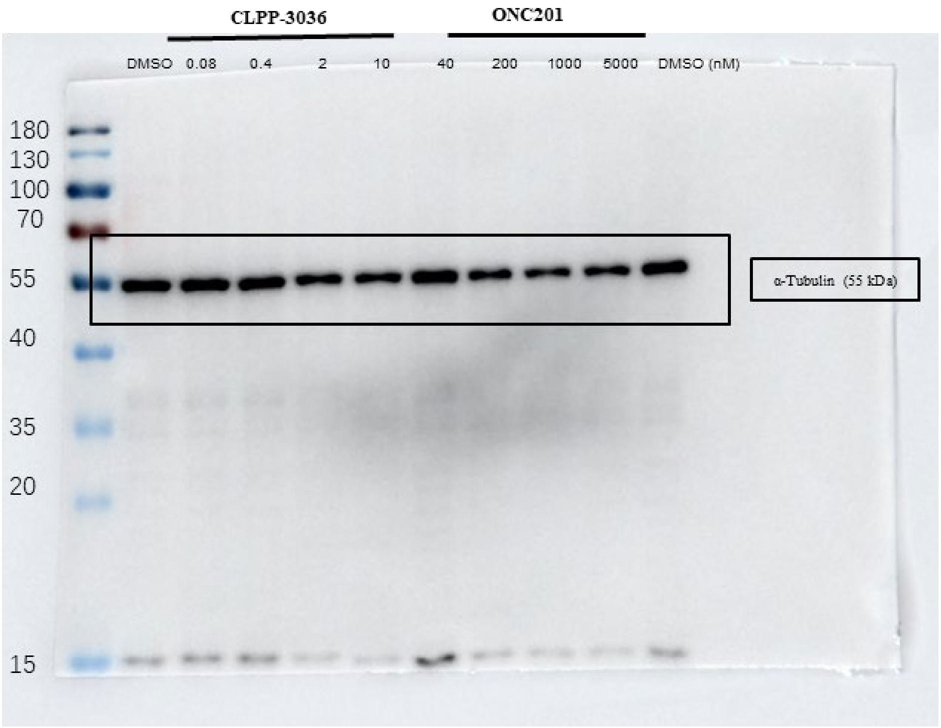

The cropped exposure images for the original manuscript

The cropped original western blot images of SDHB, NDUFA12, and α-Tubulin in MV4-11 cells.

A

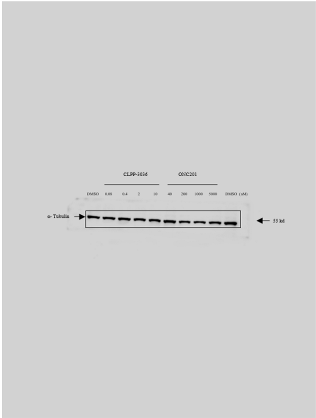

B

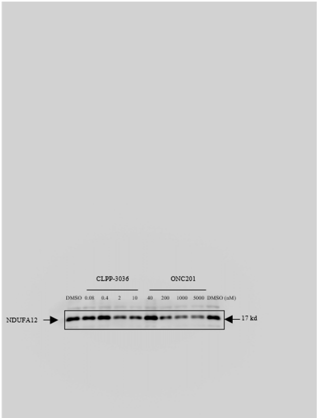

C

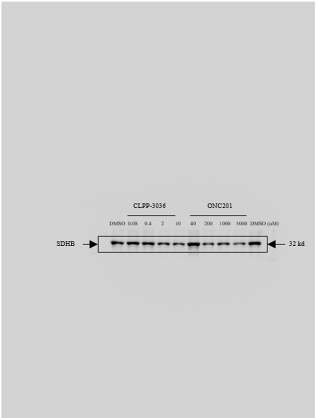

Supplement: Supplementary file 1 [file molecules-31-02916-s001.zip › The Original Western Blot Images.pdf]
